# Impact of the program life in traffic and new zero-tolerance drinking and driving law on the prevalence of driving after alcohol abuse in Brazilian capitals: An interrupted time series analysis

**DOI:** 10.1371/journal.pone.0288288

**Published:** 2023-10-20

**Authors:** Rafael Alves Guimarães, Otaliba Libânio de Morais Neto, Taciana Mirella Batista dos Santos, Polyana Maria Pimenta Mandacarú, Elaine Leandro Machado, Waleska Teixeira Caiaffa, Paulo Roberto Prezotti Filho, Érika Carvalho de Aquino, Valdério Anselmo Reisen

**Affiliations:** 1 Instituto de Patologia Tropical e Saúde Pública, Universidade Federal de Goiás, Goiânia, Goiás, Brasil; 2 Faculdade de Enfermagem, Universidade Federal de Goiás, Goiânia, Goiás, Brasil; 3 Faculdade de Medicina, Universidade Federal de Minas Gerais, Belo Horizonte, Minas Gerais, Brasil; 4 Universidade Federal do Espírito Santo, Vitória, Espírito Santo, Brasil; UFSJ: Universidade Federal de Sao Joao del-Rei, BRAZIL

## Abstract

**Introduction:**

Driving under the influence of alcohol is one of the main factors for morbidity and mortality from traffic accidents. In 2010 and 2013, the Program Life in Traffic was implemented in Brazil, including the international initiative “*Road Safety in Ten Countries*”, which established actions to reduce one of the main risk factors for road traffic injuries, the driving under the influence of alcohol. In 2012, a new zero-tolerance drinking and driving law (new dry law) was implemented, establishing a zero-tolerance limit for the drivers’ blood alcohol concentration, and increasing punitive measures. This study aimed at analyzing the impact of these measures on the prevalence of driving under the influence of alcohol abuse in Brazilian capitals.

**Methods:**

An interrupted time series study was conducted using the models of autoregressive integrated moving average or seasonal autoregressive integrated moving average. The main outcome was the prevalence of driving after alcohol abuse in the adult population (≥ 18 years). The model’s predictors were the interventions “Program Life in Traffic” and “New Dry Law”. The former was implemented in the first quarter of 2011, initially in five capitals: Belo Horizonte, Campo Grande, Palmas, Teresina, and Curitiba, being expanded to the other capitals in the first quarter of 2013. The latter was implemented in the country on the first quarter of 2013. Data source for the study was the records of the surveillance system for risk and protection factors of chronic diseases through telephone survey (Vigitel) from 2007 to 2016.

**Results:**

The time intervals considered in the study were the quarters. Thirty-eight units were considered in the analysis, corresponding to time series points. It was found that after the implementation of the Program Life in Traffic, in the first quarter of 2011, there was a reduction in the prevalence in Belo Horizonte and Curitiba. Because the introduction of the New Dry Law and the Program Life in Traffic took place in similar periods in the other cities, there was a significant reduction in the outcome prevalence in the cities of Aracaju, Belo Horizonte, Boa Vista, Fortaleza, João Pessoa, Maceió, Manaus, Palmas, Porto Alegre, Recife, Teresina, Rio Branco, and Vitória following the law application.

**Conclusion:**

The present study identified an immediate impact of the Program Life in Traffic in two capitals (Belo Horizonte and Curitiba) and a joint impact of the New Dry Law in 13 capitals. The results of this study have implications for strengthening interventions aimed at reducing the burden of morbidity and mortality from traffic accidents in Brazil.

## Introduction

In Brazil, road traffic injuries (RTI) represent a serious public health problem. These conditions are associated with high rates of morbidity and mortality, reduced life expectancy, loss of productivity, and costs for the health system [1]. The country ranks third in the number of deaths from RTI in the world, second only to India and the United States of America. In 2017, the Global Burden of Disease study estimated an occurrence of 52,326 deaths, a mortality rate of 24.8 deaths per 100,000 inhabitants, and 1,116.0 disability-adjusted life years resulting from RTI in Brazil [2], confirming the vulnerability. In addition, estimates from the Institute of Applied Economic Research are that RTI on roads and highways cost 50 billion of Brazilian Real (BRL) to the public coffers in 2014, with the highest cost related to loss of productivity, followed by hospital costs [3].

Alcohol consumption is associated with multiple social and health consequences [4]. Studies have shown that driving under the influence of alcohol corresponds to one of the main risk factors, injuries, and mortality from RTI [4, 5]. In this context, several developed and developing countries have adopted measures to reduce this risk factor, such as the prohibition of alcoholic beverages for adolescents, improvements in road infrastructure, and determination of blood alcohol concentration (BAC) limits for drivers, among other policies aimed at reducing RTI morbidity and mortality by reducing the prevalence of driving under the influence of alcohol [1, 6].

In January 2010, the international initiative “*Road Safety in Ten Countries*” (RS-10) was implemented in 10 developing countries (Russia, China, Turkey, Egypt, Vietnam, Cambodia, India, Kenya, Mexico, and Brazil). These countries were chosen because they account for almost half (48.0%) of global deaths from RTI [7]. The RS-10 initiative is funded by the Bloomberg Foundation and the participating countries’ own resources, and is coordinated globally by the World Health Organization and its regional agencies. The objective of RS-10 was to support the governments of low- and middle-income countries to incorporate road safety measures into their national policies, thus contributing to reducing the magnitude of RTI [7–10].

Despite the differences among the countries, RS-10 activities include the safety of drivers, motorcyclists, cyclists, and pedestrians, through legislation change and enforcement; health education actions in traffic; training of police and/or public health officials to implement and monitor the impact of the initiative; improvement of databases related to RTI and its risk factors; identification of determinants for RTI and deaths; carrying out periodic inspections of alcohol and driving and excessive and/or inappropriate speed; holding selected public events and media strategies to disseminate the dangers of alcohol, incentives in the purchase of blood alcohol and over speed monitoring equipment, among others. In general, the actions are mainly focused on the factors driving at excessive/inadequate speed and under the influence of alcohol [7–9].

In Brazil, the RS-10 initiative was called Project Life in Traffic (in Portuguese, *Projeto Vida no Trânsito*—PLT), being coordinated by the Ministry of Health in partnership with the Pan-American Health Organization [9, 11]. The PLT was inserted in a global context to achieve the objective of the Decade of Road Safety 2011–2020, as established by the United Nations [2, 9, 11]. It was initially implemented in the first half of 2011, in the capital cities of Palmas, Teresina, Campo Grande, Belo Horizonte and Curitiba, representing, respectively, the North, Northeast, Central-West, Southeast, and South Brazilian macro-regions [9, 11].

In the first half of 2013, the program was expanded to all Brazilian capitals and cities with more than one million inhabitants, except for the city of Rio de Janeiro (State of Rio de Janeiro, Southeast Region). The PLT aims to reduce two risk factors: driving under the influence of alcohol and at excessive and/or inappropriate speed, classified as intermediate indicators of traffic safety [9, 11]. These factors directly influence the final indicators represented by the RTI hospitalization and mortality rate. In Brazil, PLT follows the same activities recommended by RS-10, although the adhering municipalities have the autonomy to establish key actions depending on the local reality [9, 11].

Brazil also has extensive legislation to control drinking and driving. In 1989, the country introduced a legal BAC limit of 0.8 g/L, reducing this value to 0.6 g/l in January 1998 through the Brazilian Traffic Code [12]. However, the inadequate application of the law in force led the government to implement new legislation. On June 19, 2008, the federal government passed a new legislation called Dry Law (in Portuguese, *Lei Seca*). This law established a zero-tolerance blood alcohol limit for drivers [13]. The identification of BAC imposed important penalties on the driver, such as a fine, suspension of the right to drive for 12 months, and vehicle impoundment [13, 14]. When a BAC greater than 0.6 g/L is identified, the law also includes imprisonment [13]. The legislation also prohibited the sale of alcoholic beverages on federal highways, aiming to reduce access to alcohol. On December 20, 2012, this law was reformulated, being entitled new zero-tolerance drinking and driving law (new dry law—NDL), with number 12.760 [15], idealized in the context of the RS-10. The new legislation enhanced punishment for driving under the impact of any amount of alcohol, such as increasing the fine by 10 times, in addition to maintaining the suspension of the right to drive with loss of license and vehicle impoundment [15]. NDL actions include sobriety checkpoints and shall integrate the PLT plan of the municipalities. In 2016, the punishment became more severe with Law no. 13.281, which established that the refusal to carry out the breath test constitutes a very serious infraction, with an increase in the fine value, collection of the license document, vehicle impoundment, and the possibility of suspension of the right to drive [16].

There is a gap in the literature on the impacts of PLT and NDL on the prevalence of driving under the influence of alcohol. International studies evaluating the PLT focused on the analysis of final indicators, such as the mortality rate and hospitalization for RTI, or other intermediate indicators, such as seat belt use and over speed driving [10, 17]. In Brazil, only one study was identified that analyzed the impacts of PLT in five Brazilian cities, showing a reduction in the mortality rate indicators in three and in the prevalence of drivers with positive BAC in two [11]. Previous investigations into the impact of drinking and driving restrictions conducted have also analyzed the impact, predominantly, on RTI mortality and/or hospitalization rates [18–22]. In Brazil, only two studies analyzed the impact of the first dry law on the prevalence of driving under the influence of alcohol abuse [23, 24]. However, assessments were limited to comparing prevalence before and after this legislation, without controlling for temporal trends and potential seasonality. In this context, the purpose of the present study was to analyze the impact of PLT and NDL on the safety indicator “driving under the influence of alcohol abuse” in Brazilian capitals.

## Materials and methods

### Study design and locations

This is an interrupted time series analysis (ITS) to assess the impact of PLT and RTI on the prevalence of driving under the influence of alcohol abuse in Brazilian capitals. The study was developed considering as units of analysis the set of 27 Brazilian capitals, distributed in 26 units of the federation and the Federal District of the country of all five macro-regions: North, Northeast, Central-West, South, and Southeast.

In this study, the impact of the PLT was analyzed in 25 capitals out of the 27, excluding the city of Rio de Janeiro that did not join the program. The NDL is national and mandatory, but the program is implemented upon adherence by the municipality. Thus, for the analysis of the impact of the NDL, all 27 capitals were included, as shown in Table 1.

**Table 1:** Periods of implementation of the program life in traffic and the new dry law in Brazilian capitals.

Capitals	Start of PLT actions	Start of NDL application
Belo Horizonte, Campo Grande, Curitiba, Teresina, and Palmas	1st quarter of 2011 (2011q1)	1st quarter of 2013 (2013q1)
São Paulo, Vitória, Porto Alegre, Florianópolis, Cuiabá, Goiânia, Brasília, Macapá, Belém, Manaus, Rio Branco, Porto Velho, Boa Vista, Palmas, Maceió, Salvador, Ceará, São Luís, João Pessoa, Recife, Teresina, Sergipe, and Natal	1st quarter of 2013 (2013q1)	1st quarter of 2013 (2013q1)
Rio de Janeiro	*	1st quarter of 2013 (2013q1)

**Abbreviations**: NDL = New Dry Law; PLT = Project Life in Traffic; q = quarter.

*PLT was not implemented in this capital.

The impact of PLT and NDL were analyzed separately in the cities of Belo Horizonte, Campo Grande, Curitiba, Palmas, and Teresina, since these cities implemented the program two years before the application of the NDL (from the 1st quarter of 2011). For the other capitals, the impact of the PLT and NDL were analyzed together since the program and the law were implemented in similar periods of time (starting on the 1st semester of 2013). It should be noted that the main component of the NDL are the inspections at sobriety checkpoints, which are also included within the PLT actions in the municipalities, with a residual effect in both interventions. Finally, for Rio de Janeiro, only the impact of NDL was evaluated (Table 1).

### Data source

In this study, data from the Surveillance System of Risk and Protection Factors for Chronic Diseases through Telephone Survey (Vigitel) from 2007 to 2016 were used [25–27].

Vigitel was implemented in 2006 to monitor the main risk and protection factors for non-communicable diseases and conditions, through telephone interviews. This is a home study conducted in 26 Brazilian capitals and in the federal capital Brasília. This survey is one of the components of the Surveillance System for Risk Factors for Chronic Noncommunicable Diseases of the Brazilian Ministry of Health [25, 26]. This investigation used only data on the prevalence of driving under the influence of abusive use of alcohol from 2007 on, the year when the indicator started to be monitored by Vigitel [23].

Briefly, Vigitel is a cross-sectional study that aims to obtain, in each of the capitals, probabilistic samples of adults (≥ 18 years old) residing in households that have at least one landline. The telephone numbers used for sampling are provided free of charge by the main telephone companies in the country [25, 26]. The sample size is approximately 2,000 adults for each capital, to estimate a confidence interval of 95% (95%CI) and a maximum error of two percentage points for the prevalence of the main risk factors for chronic non-communicable diseases [26].

Initially, 5,000 landlines are drawn for each city. This procedure is systematic and stratified by postal code. These samples are then re-divided into 25 replicates with a size of 200 landlines in each replicate, using the same drawing process as the initial sample [25, 26].

In the second stage, one of the adult residents of the households selected from the selected landlines eligible for the study is drawn. Eligible lines are those active residential lines in each city. Lines belonging to companies, lines that no longer exist or are out of service, and lines that have not responded to six attempts of calls made on alternative days and times, including Saturdays, Sundays and at night, are considered ineligible [25, 26]. Finally, the selected resident is interviewed by trained professionals about sociodemographic data and main risk or protective factors for chronic non-communicable diseases. It should be noted that in Vigitel, in most years, the sample of each city is divided by month, allowing the estimation of indicators on a monthly or quarterly basis. Due to the small sample size in each month, in the present study the variable of interest in the quarter of each year was analyzed.

### Variables

The dependent variable “*driving under the influence of alcohol abuse*” was analyzed, calculated for each quarter. The numerator of this indicator consisted of all adults who reported driving a motor vehicle after alcohol abuse and who responded positively to the questions: "*In the last 30 days*, *have you drunk four or more doses of alcoholic beverage on a single occasion*?” for women and “*On this day (or any of these days)*, *did you drive right after drinking*? (women) or "*In the last 30 days*, *have you drunk five or more doses of alcoholic beverage on a single occasion*? and "*On this day (or any of these days)*, *did you drive right after drinking*? (men). In Vigitel, a dose of alcoholic beverage is a dose of distilled beverage, a can of beer, or a glass of wine [23]. The denominator consisted of the total number of adults interviewed in each quarter [28]. Prevalence was calculated by dividing the numerator by the denominator, multiplying by 100.

Also, two dummy binary variables were constructed and used as independent variables to represent the interventions analyzed in different cities, as can be seen in Table 2.

**Table 2:** Periods of implementation of the program life in traffic and the new dry law in Brazilian capitals.

Dummy variables	Pre-intervention period	Post-intervention period
Belo Horizonte, Campo Grande, Curitiba, Palmas, and Teresina		
PLT	*0*-3rd quarter of 2007 (2007q3) to 4th quarter of 2010 (2010q4)	*1-* 1st quarter of 2011 (2011q1) to 4th quarter of 2016 (2016q4)
NDL	*0*-3rd quarter of 2007 (2007q3) to 4th quarter of 2010 (2010q4)	*1-*1st quarter of 2011 (2011q1) to 4th quarter of 2016 (2016q4)
São Paulo, Vitória, Porto Alegre, Florianópolis, Cuiabá, Goiânia, Brasília, Macapá, Belém, Manaus, Rio Branco, Porto Velho, Boa Vista, Palmas, Maceió, Salvador, Ceará, São Luís, João Pessoa, Recife, Teresina, Sergipe, and Natal		
PLT	*0*-3rd quarter of 2007 (2007q3) to 4th quarter of 2012 (2012q4)	*0*-1st quarter of 2013 (2013q1) to 4th quarter of 2016 (2016q4)
Rio de Janeiro		
NDL	*	1st quarter of 2013 (2013q1) to 4th quarter of 2016 (2016q4)

**Abbreviations**: NDL = New Dry Law; PLT = Project Life in Traffic; q = quarter.

*PLT was not implemented in this capital.

This approach, using two dummy variables, allowed the analysis of the impacts of the PLT and NDL on the outcome in the five capitals that implemented the PLT in 2011: Belo Horizonte, Campo Grande, Curitiba, Palmas, and Teresina (Table 2).

A third binary dummy variable was used to analyze the impact of interventions in other cities. This approach allowed the analysis of the impact of the PLT together with the NDL, which was necessary since both interventions were implemented in the other capitals in similar periods of time (2013q1) (Table 2).

### Statistical analysis

Assessments of the intervention impacts were conducted for each capital under study. The analyses were performed in the following steps: (i) descriptive statistics; (ii) model identification; (iii) estimation of model parameters and (iv) model verification. The analyses were conducted based on the adaptation of Box & Jenkins methodology [29].

Initially, descriptive statistics of the time series were performed. The time intervals considered in the study were the quarters. Each series analyzed had three missing values (2008q1, 2012q2 and 2015q1). Thus, the missing values were imputed by the average of the respective series [30], resulting in a total of 38 points analyzed. The descriptive analysis included the construction of scatter plots of outcome prevalence and descriptive statistics (mean, 95% CI of mean, standard deviation [SD], variance, median, minimum and maximum) [31, 32].

An ITS analysis was used in this study, using Autoregressive Integrated Moving Average (ARIMA) or Seasonal Autoregressive Integrated Moving Average (SARIMA) models. The ARIMA model (p,d,q) corresponds to an extension of an autoregressive model (AR), moving average model (MA), and Autoregressive Moving Average (ARMA) model [29, 33]. The parameters of the ARIMA model are: *p* = autoregression order; *d* = order of differing; and *q* = order of the moving average.

The ARIMA model is used when the time series does not show seasonality. In case of seasonality or seasonal patterns, the use of the model SARIMA is required, which has the following structure: SARIMA (p,d,q) (P,D,Q) [S] [29, 33]. This model parameters are: *p* = autoregression order; *d* = degree of difference; *q* = moving average order; *P* = seasonal autoregression; *D* = seasonal integration, *Q* = seasonal moving average, and *S* = duration of the seasonal period.

Modeling was developed in three stages: (i) identification, (ii) estimation, and (iii) verification.

The identification stage consisted of selecting the seasonal (p,q,d) and/or non-seasonal (P, Q, D) orders models. Initially, time series were decomposed into their classical components: trend-cycle, seasonality, and irregularity [34]. The decomposition procedure allowed a better understanding of the series behavior and patterns, helping in the following stages of the modeling [34]. Then, the test of *Mann-Kendall* (MK) was performed to detect the presence of a deterministic trend in the time series [35], which has greater statistical power when compared to other tests for trend, such as *Cox-Stuart* [36] test. In case of presence of trend, one or more orders *d* were applied in the models to eliminate the trend and achieve stationarity around the mean. Moreover, the test of *Dickey-Fuller* (ADF) was used as an additional test to verify the presence of time series stationarity [37]. The presence of seasonality patterns was initially analyzed by visual inspection of the data and the decomposed series [38]. Furthermore, tests of Kruskal-Wallis and one-way ANOVA were used to test for the presence of stable seasonality and the two-way ANOVA (F test) for detecting moving seasonality after trend removal if present. In case of seasonality, the series seasonal adjustment was performed [34].

Following trend removal and/or seasonal adjustment, the autocorrelation (ACF) and partial autocorrelation (PACF) functions graphs were analyzed for the detection of the models’ *p* and *q* orders [29]. Finally, the seasonal component was included using SARIMA models in case the previous analysis indicated evidence of seasonality [39].

To find the best model, multiple modelings were performed with the respective diagnostic validation [39]. The quality of the models was analyzed through the Akaike information criterion (AIC) [40] as well as the verification of Mean Error (ME); root mean squared error (RMSE); mean absolute error (MAE); mean percentage error (MPE); Mean absolute percentage error (MAPE); Mean absolute scaled error (MASE). Finally, analysis of the model’s residuals was carried out through the ACF graphics of the residuals and the Ljung-Box test with a lag of 10 to verify the absence or presence of residual autocorrelation. The tests of Kolmogorov-Smirnov (KS), Anderson-Darling (AD), and Shapiro-Wilk (SW) were used to verify the models’ residuals normality [41, 42]. In case of significant autocorrelation in the models and/or absence of residuals normality, the initial step was resumed for a new modeling to be carried out. The model selected was the one presenting the lowest AIC and BIC (N’Gattia et al., 2016), provided that the inclusion of trend and seasonality terms was respected if their presence was detected and/or did not present autocorrelation or deviations from residuals normality.

In all tests and analyses, *p*-values < 0.05 were considered statistically significant.

Data were analyzed using the statistical software STATA, version 16.0, and the R software was used for time series modeling.

### Ethical aspects

The Vigitel study was approved by the National Human Research Ethics Committee (CONEP) of the Brazilian Ministry of Health in all years of the study. The survey did not require written consent from the participant, and verbal consent was used. Verbal consent was obtained from all participants. This specific study used the anonymized Vigitel’s database.

## Results

### Descriptive analysis

Table 3 presents the descriptive analysis of the time series. The five highest mean prevalences were observed in the following capitals: Palmas (State of Tocantins/3.19%), Teresina (State of Piauí/3.10%), Boa Vista (State of Roraima/2.67%), Cuiabá (State of Mato Grosso/2.65%), and Goiânia (State of Goiás/2.50%). On the other hand, the lowest mean frequencies were found in São Paulo (State of São Paulo/1.00%), Rio de Janeiro (State of Rio de Janeiro/1.08%), Vitória (State of Espírito Santo/1.18%), Manaus (State of Amazonas/1.20%), Belém (State of Pará/1.21%), and Salvador (State of Bahia/1.21%).

**Table 3:** Descriptive analysis of the prevalence of driving after alcohol abuse in Brazilian capitals, VIGITEL Study, 2007q3 to 2016q4.

City	Mean	95.0% CI	SD	Variance	Median	Min-Max
Aracaju	2.28	1.98–2.78	1.17	1.36	2.41	0.1–4.8
Belém	1.21	0.99–1.43	0.65	0.43	1.24	0.0–2.7
Belo Horizonte	1.64	1.32–1.97	0.93	0.88	1.53	0.0–3.9
Boa Vista	2.67	2.30–3.05	1.08	1.18	2.58	0.6–5.5
Brasília	2.28	1.89–2.69	1.16	1.34	2.04	0.7–5.9
Campo Grande	2.08	1.76–3.40	0.93	2.01	2.01	0.2–3.8
Cuiabá	2.65	2.30–2.98	0.98	0.96	2.65	0.0–4.3
Curitiba	1.43	1.19–1.69	0.71	0.51	1.33	0.2–3.1
Florianópolis	2.37	1.97–2.77	1.16	1.34	2.26	0.7–6.0
Fortaleza	1.67	1.33–2.00	0.97	0.94	1.64	0.2–3.8
Goiânia	2.50	2.07–2.94	1.27	1.60	2.44	0.0–6.8
João Pessoa	1.71	1.31–2.10	1.16	1.33	1.33	0.0–3.8
Macapá	2.03	1.54–2.52	1.43	2.04	1.75	0.1–7.1
Maceió	1.26	0.94–1.58	0.92	0.85	1.08	0.0–3.3
Manaus	1.20	0.94–1.46	0.76	0.57	1.13	0.0–3.6
Natal	1.71	1.37–2.06	0.99	0.99	0.99	0.0–4.7
Palmas	3.19	2.82–3.56	1.07	1.14	3.25	1.1–5.1
Porto Alegre	1.26	0.76–1.75	1.43	2.07	0.87	0.1–8.1
Porto Velho	2.07	1.79–2.36	0.82	0.68	2.24	0.4–3.7
Recife	1.26	0.97–1.56	0.86	0.73	1.23	0.0–2.8
Rio Branco	1.92	1.36–2.49	1.64	2.71	1.66	0.0–9.3
Rio de Janeiro	1.08	0.82–1.33	0.74	0.55	0.94	0.0–3.2
Salvador	1.21	0.95–1.48	0.78	0.60	0.88	0.0–3.5
São Luís	2.19	1.84–2.55	1.04	1.08	2.09	0.7–5.5
São Paulo	1.00	0.81–1.20	0.57	0.33	1.04	0.1–2.2
Teresina	3.10	2.60–3.61	1.47	2.17	2.79	0.8–8.4
Vitória	1.18	0.91–1.44	0.78	1.03	1.03	0.0–3.1

**Abbreviations**: 95.0% CI: 95.0% Confidence Interval; SD: Standard deviation; Min: Minimum; Max: Maximum.

**Fig 1:**
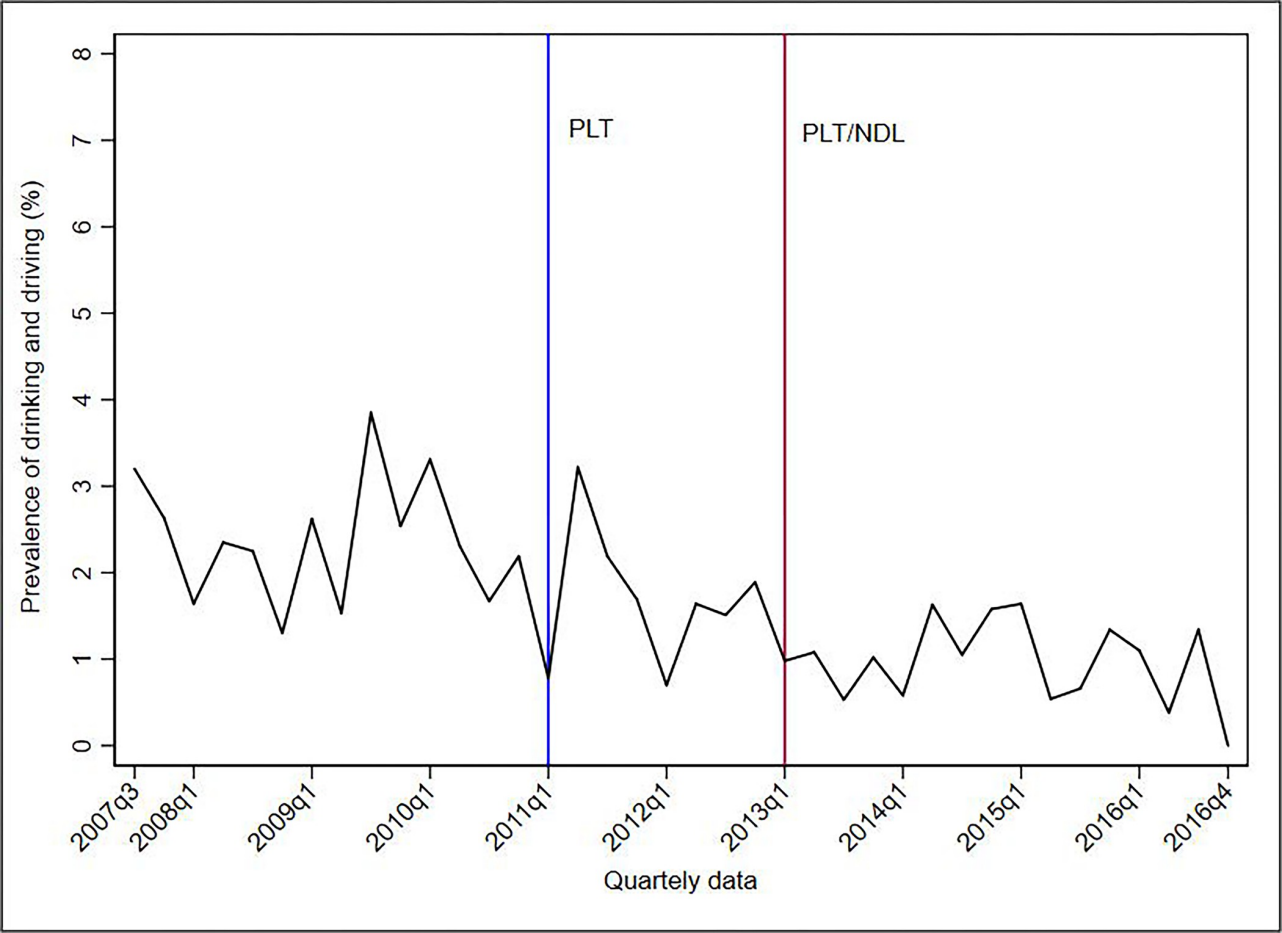
Descriptive analysis of the quarterly prevalence of driving under the influence of alcohol abuse after the implementation of the PLT and NDL in Belo Horizonte, 2007–2016. Abbreviations: NDL = New Dry Law; PLT = Project Life in Traffic.

**Fig 2:**
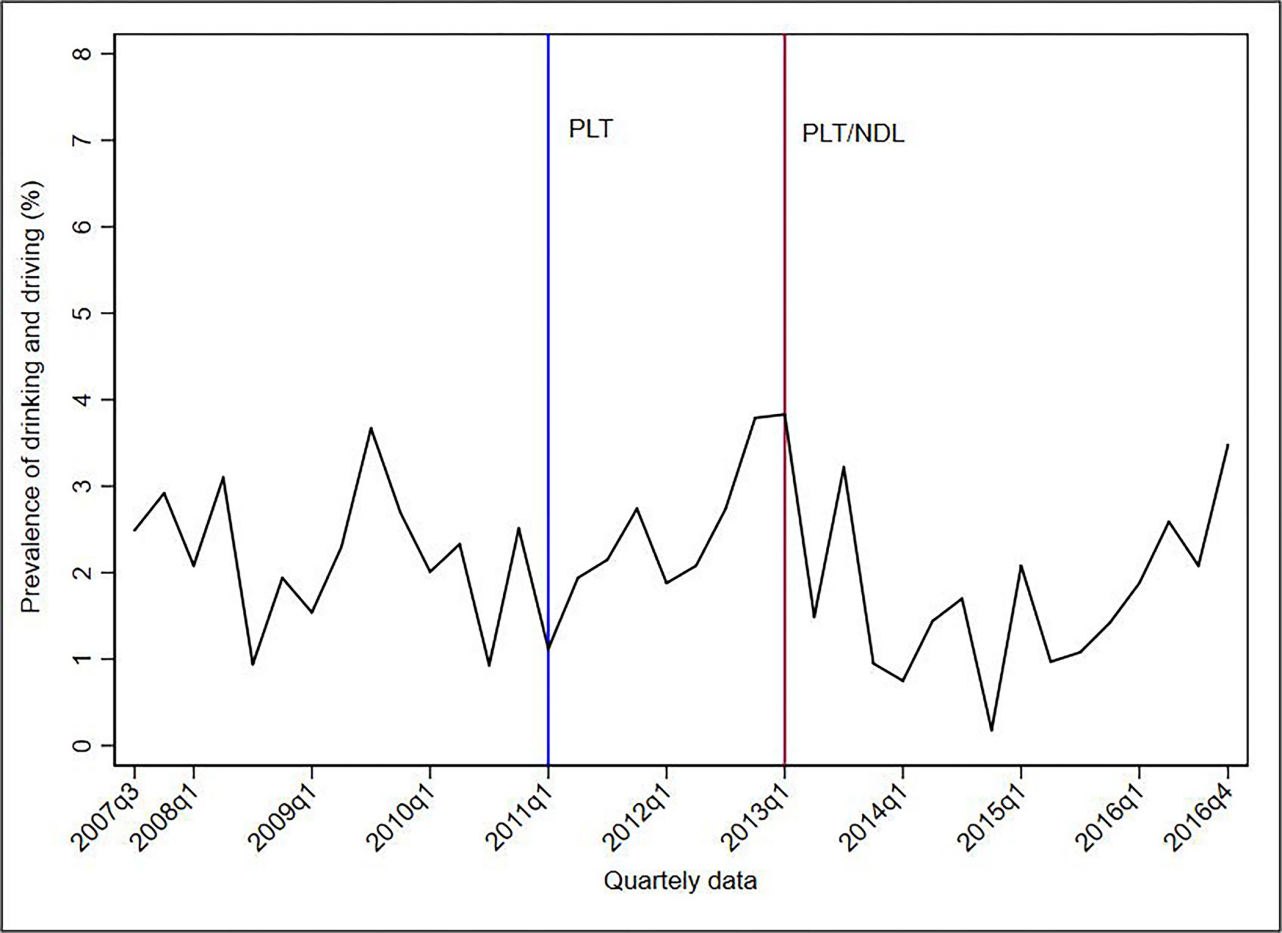
Descriptive analysis of the quarterly prevalence of driving under the influence of alcohol abuse after the implementation of the PLT and NDL in Campo Grande, 2007–2016. **Abbreviations**: NDL = New Dry Law; PLT = Project Life in Traffic.

**Fig 3:**
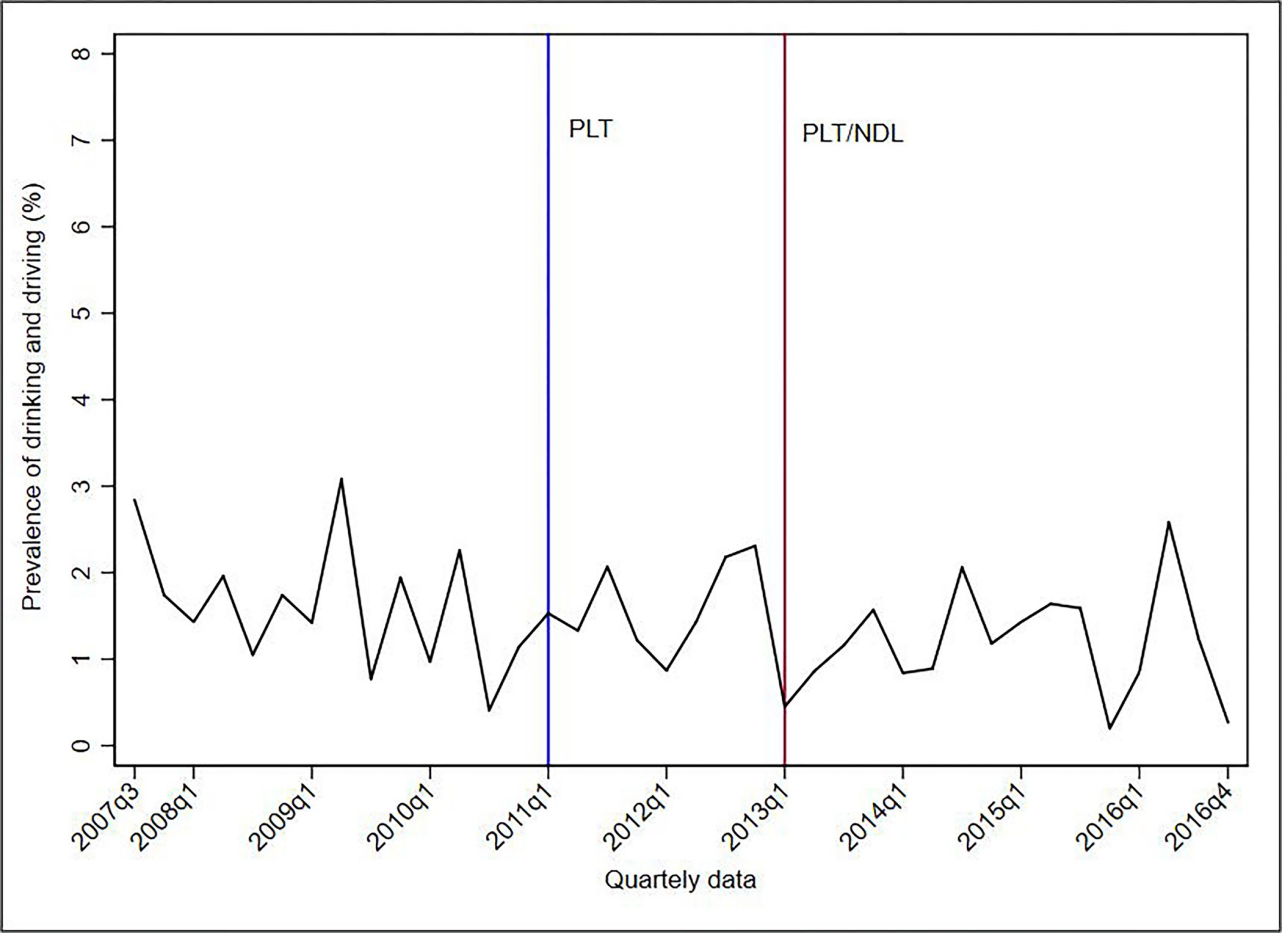
Descriptive analysis of the quarterly prevalence of driving under the influence of alcohol abuse after the implementation of the PLT and NDL in Curitiba, 2007–2016. **Abbreviations**: NDL = New Dry Law; PLT = Project Life in Traffic.

**Fig 4:**
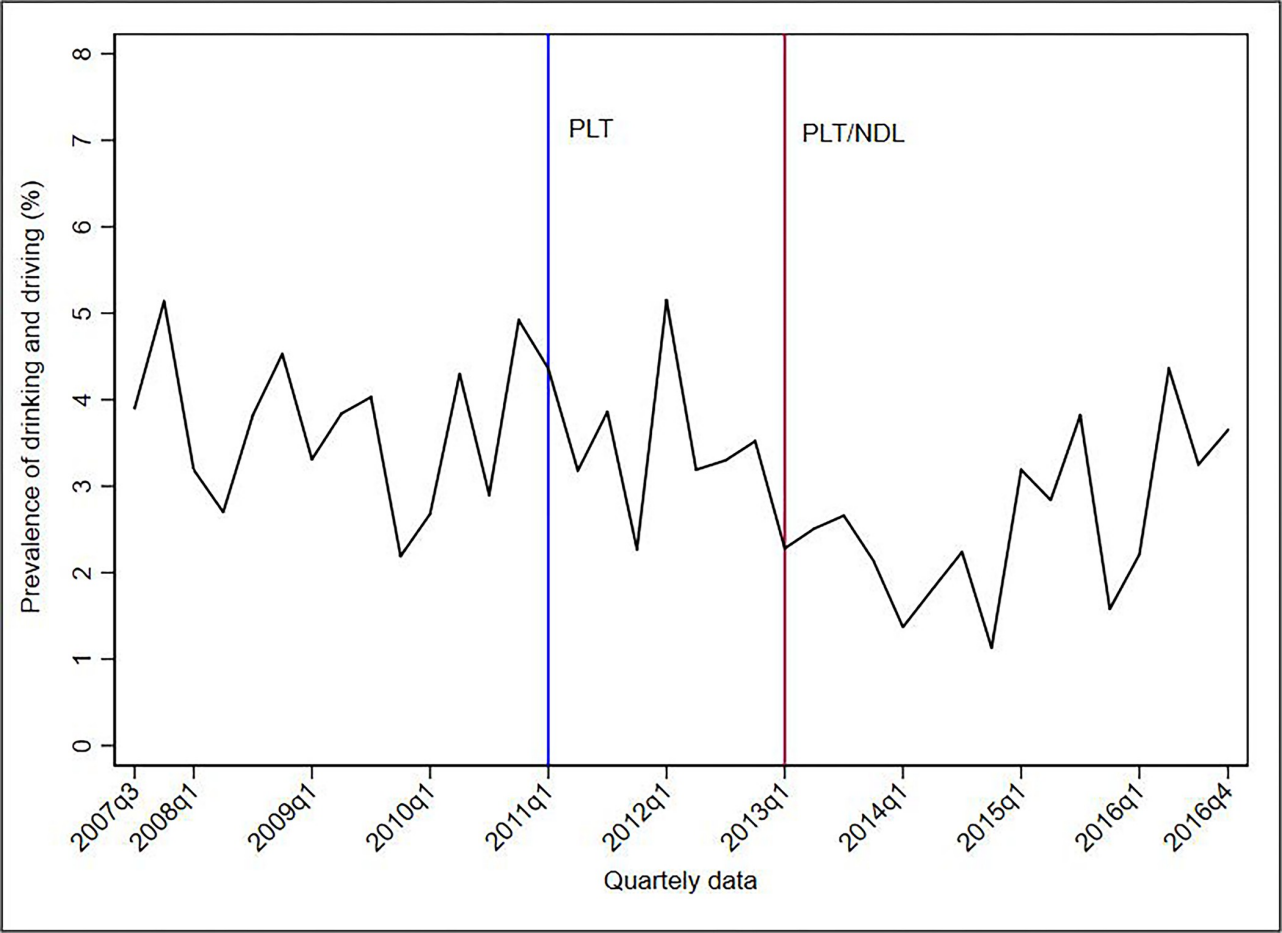
Descriptive analysis of the quarterly prevalence of driving under the influence of alcohol abuse after the implementation of the PLT and NDL in Palmas, 2007–2016. **Abbreviations**: NDL = New Dry Law; PLT = Project Life in Traffic.

**Fig 5:**
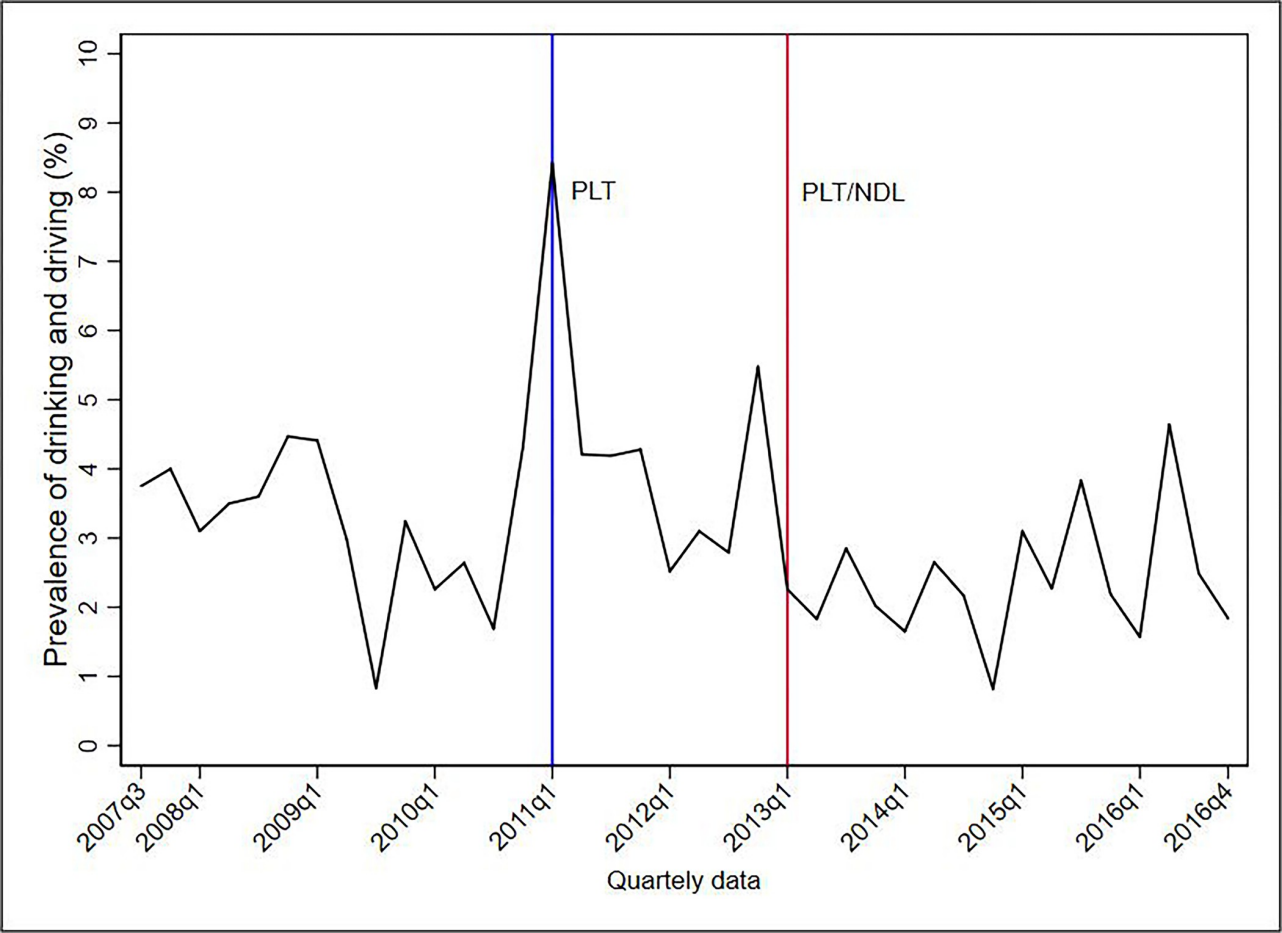
Descriptive analysis of the quarterly prevalence of driving under the influence of alcohol abuse after the implementation of the PLT and NDL in Teresina, 2007–2016. **Abbreviations**: NDL = New Dry Law; PLT = Project Life in Traffic.

Figs 1–5 show the descriptive analysis of outcome prevalence in the first five capitals of the PLT implementation in the first quarter of 2011 (2011q1), followed by the application of the NDL in the first quarter of 2013 (2013q1).

The descriptive analysis of the quarterly prevalence of driving under the influence of alcohol abuse in the capitals with the implementation of the PLT together with the beginning of the application of the NDL in the first quarter of 2013 (2013q1) is presented in S1 File.

### Impact of interventions

Before conducting the analyses, the presence of outliers was observed and, in case of their presence, the automatic replacement for the respective mean in the R software was performed. S2 File shows the decomposition analysis of the time series; S3 File presents the ACF and PACF in the model identification stage and S1 Table shows the analysis of the trend, stationarity, and seasonality tests. These analyses were carried out to identify the orders of the time series models.

Table 4 shows the impact of PLT and NDL on the prevalence of drink-driving in the first five capitals that implemented the PLT, still in the first quarter of 2011. The results showed that the prevalence significantly reduced, on average, 0.740% and 0.217% after the implementation of the PLT in Belo Horizonte (β: -0.740; p-value< 0.001). In Curitiba, this reduction was, on average, 0.217% (β: -0.217; p-value< 0.001). No significant impact was observed on this outcome soon after the implementation of the PLT in Palmas (β: -0.07; p-value = 0.856), Campo Grande (β: -0.014; p-value = 0.975), and Teresina (β: 0.571; p-value = 0.166). Therefore, the impact of PVT was observed in Belo Horizonte and Curitiba.

**Table 4:** Effect of PLT and NDL on driving prevalence after alcohol abuse, Vigitel Study, 2007q3 to 2016q4*.

City	Interventions	Model	Intervention effect			
			β	95.0% CI	SE	*p*-value
Belo Horizonte		ARIMA (4,1,2)				
	PLT		-0.740	-0.892; -0.589	0.077	**< 0.001**
	NDL		-0.649	-0.789; -0.510	0.071	**< 0.001**
Campo Grande		ARIMA (0,0,2)				
	PLT		-0.014	-0.918; 0.889	0.460	0.975
	NDL		-0.494	-1.381; 0.393	0.453	0.275
Curitiba		ARIMA (1,0,1)				
	PLT		-0.217	-0.395; -0.039	0,090	**0,017**
	NDL		-0.110	-0.280; 0,059	0,086	0.203
Palmas						
	PLT	ARIMA (1,1,1)	-0.07	-0.80; 0.67	0.37	0.856
	NDL		-1.04	-1.76; -0.32	0.37	**0.007**
Teresina		ARIMA (1,1,1)				
	PLT		0.571	-0.238; 1.381	0.413	0.166
	NDL		-1.377	-2.170; -0.584	0.405	**0.007**

**Abbreviations:** PLT: Program Life in Traffic; NDL: New Dry Law; 95% CI: Confidence Interval of 95%; SE: Standard Error; Time series analysis based on ARIMA models (p,d,q).

*Considering only the capitals with the beginning of the PLT in 2011q1.

After the implementation of the NDL, there was a significant reduction in the prevalence of the outcome in Belo Horizonte by 0.649% (β: -0.649; p-value<0.001), in Palmas by 1.04% (β: -1.040; p-value<0.007), in Teresina by 1.377% (β: -1.377; p-value<0.001), and without significant impact in Campo Grande (β: -0.494; p-value = 0.275) and Curitiba (β: -0.110; p-value = 203). Therefore, the impact of the NDL was observed in Belo Horizonte, Palmas, and Teresina (Table 4).

Table 5 shows the results of the PLT/NDL impact models on the quarterly prevalence of driving under the influence of alcohol abuse in other Brazilian capitals. A significant reduction was observed in the following capitals: Aracaju, Boa Vista, Fortaleza, João Pessoa, Maceió, Manaus, Porto Alegre, Recife, Rio Branco, and Vitória (*p*-value< 0.05). For instance, the interpretation of regression coefficients showed that the prevalence of driving under the influence of alcohol abuse reduced, on average, 1.883% every quarter in Rio Branco (β: -1.883; *p*-value<0.001) and 0.223% in Boa Vista (β: -0.223; *p*-value = 0.024).

**Table 5:** Effect of PLT and NDL on driving prevalence after alcohol abuse, Vigitel Study, 2007q3 to 2016q4*.

City	Model	Interventions	Intervention effect			
			β	IC 95%	SE	p-value
Aracaju	(0,1,1)	PLT/NDL	-1.179	-1.791; -0.566	0.312	**0.001**
Belém	(1,0,1)	PLT/NDL	-0.104	-0.254; 0,047	0.076	0.176
Boa Vista	(1,0,1)	PLT/NDL	-0.223	-0.418; -0.030	0.098	**0.024**
Cuiabá	(1,0,1)	PLT/NDL	-0.261	-0.680; 0.158	0.214	0.222
Florianópolis	(1,0,1)	PLT/NDL	-0.434	-1.125; 0.256	0.353	0.218
Fortaleza	(0,1,1)	PLT/NDL	-0.768	-1.312; -0.224	0.278	**0.006**
Goiânia	(0,0,0) (1,0,2) [4]	PLT/NDL	-0.271	-0.770; 0.227	0.254	0.286
João Pessoa	(4,1,2)	PLT/NDL	-1.343	-1.596; -1.090	0.129	**< 0.001**
Macapá	(1,1,0)	PLT/NDL	-1.490	-3.297; 0.316	0.922	0.106
Maceió	(3,1,3)	PLT/NDL	-0.421	-0.799; -0.027	0.197	**0.036**
Manaus	(2,1,2) (1,0,0) [4]	PLT/NDL	-0.503	-0.832; -0.174	0.168	**0.003**
Natal	(3,1,1)	PLT/NDL	1.029	-0.274; 2.334	0.665	0.121
Porto Alegre	(1,0,3)	PLT/NDL	-0.282	-0.492; -0.072	0.107	**0.008**
Porto Velho	(1,1,1) (0,1,1) [4]	PLT/NDL	0.435	-1.427; 0.556	0.506	0.389
Recife	(0,1,1)	PLT/NDL	-1.035	-1.445; -0.626	0.209	**< 0.001**
Rio Branco	(0,1,3) (3,1,1) [4]	PLT/NDL	-1.883	-2.816; -0.950	0.476	**< 0.001**
Rio de Janeiro	(3,1,0)	NDL	-0.284	-1.829 1.259	0.788	0.718
Salvador	(0,1,4)	PLT/NDL	-0.114	-0.631; 0.401	0.263	0.663
São Luís	(3,0,0)	PLT/NDL	-0.023	-0.483; 0.435	0.234	0.919
São Paulo	(2,0,0) (1,0,0) [4]	PLT/NDL	-0.403	-1.161; 0.355	0.389	0.297
Vitória	(0,1,1)	PLT/NDL	-1.117	-1.647; -0.587	0.270	**< 0.001**
Brasília	(0,0,0)	PLT/NDL	0.002	-0.597; 0.601	0.305	0.995

Abbreviations: PLT: Program Life in Traffic; NDL: New Dry Law; 95% CI: Confidence Interval of 95%; SE: Standard Error; Time series analysis based on ARIMA models (p,d,q) or SARIMA (P, Q, D) [S].

*Considering only the capitals with the beginning of the PLT in 2013q1.

S2 Table shows the parameters for evaluating the quality of the models. S3 Table presents the diagnostic tests for the estimated final models’ residuals. All models showed no significant autocorrelations (*p*-value in the Ljung-Box test>0.05) and residuals normality verified in at least one normality test (S4 File).

## Discussion

To our knowledge, this investigation represents the first study to assess the impact of traffic safety intervention programs on this indicator in Brazil, using ITS models. The results of this study showed a significant immediate reduction in the prevalence of driving under alcohol abuse after the implementation of PLT in Belo Horizonte (-52.29% every quarter) and Curitiba (19.51% every semester). Since the introduction of the PLT and NDL in similar periods in the other cities (2013q1), there was a significant reduction in the prevalence of the outcome in the cities of Aracaju, Belo Horizonte, Boa Vista, Fortaleza, João Pessoa, Maceió, Manaus, Palmas, Porto Alegre, Recife, Teresina, Rio Branco, and Vitória after law application.

The development and effective implementation of national programs allow raising awareness in the field of road safety in the countries. The programs prioritized should be those with effective interventions for preventing deaths and RTI or reducing risk factors and, in addition, presenting the best cost-benefits [43, 44]. The main road safety problems faced by countries correspond to the drivers’ risky behaviors, especially for not using seat belts, driving at excessive and/or inappropriate speeds, and driving under the influence of alcohol and/or illicit drugs [43, 45, 46]. Key interventions to address these problems reported in the literature include health education, publicity, massive enforcement of legislation, and periodic inspection [43, 47].

In Brazil, prevention actions predominantly include the application of zero-tolerance laws for alcohol consumption and increased law austerities. There is little evidence on the impacts of PLT on road safety indicators. A single study evaluated the PLT in five Brazilian capitals and found that there was a significant reduction in the mortality rate from TA in Belo Horizonte, Palmas, and Teresina, stability in Curitiba, and a small increase in Campo Grande after the program implementation [11]. In addition, there was a reduction in the prevalence of drivers with positive BAC at sobriety checkpoints in Palmas and Belo Horizonte after the program was implemented, but a percentage increase in Teresina [11]. The present study adds evidence to the literature of the isolated impact of the PLT in two Brazilian capitals, and the potential impact together with the application of NDL in several capitals.

This investigation showed a reduction in the outcome after application of NDL as for the first quarter of 2013. Studies analyzing the impacts of laws to reduce or limit blood alcohol concentration in other countries are limited to the assessment of the occurrence of accidents, injuries, and deaths, not analyzing intermediate indicators such as the prevalence of driving after alcohol consumption. For example, in Japan, the reduction of BAC from 0.05 mg/mL to 0.03 mg/mL and increase in penalties in 2002 led to the reduction of all final safety indicators, such as serious injuries and deaths in the traffic, serious injuries and deaths caused by alcohol use, and alcohol-related death rate [18]. In the United States of America, the zero-tolerance law for alcohol in young people under 21 years of age has been associated with a significant reduction in fatal driving-related crashes after drinking alcohol [48]. In Chile, the reduction of the legal limit of drivers’ blood alcohol content from 0.05 to 0.03 g/dL showed a significant reduction of 32% of alcohol-related car accidents and 31% of RTI [20]. A study in Taiwan found that the prevalence of drinking and driving significantly decreased from 10.99% to 6.64% after BAC limit lowering in 2013 [49].

In Brazil, previous studies predominantly analyzed the impact of the first phase of Prohibition in July 2008. An ITS analysis in the cities of Belo Horizonte, Rio de Janeiro, and São Paulo (Southeast Region of Brazil) found no significant reduction in the RTI mortality rate after the implementation of the first dry law [19]. In São Paulo, a study that analyzed the impact of the first prohibition using ARIMA modeling showed that this legislation was responsible for a significant reduction in the traffic accident and mortality rates. The new legislation reduced the average monthly mortality rate in the state by 7.2% and traffic injury rates by 1.8% [50]. In Belo Horizonte (Southeast Region), an *ex-ante* and *ex-post* investigation using data from cross-sectional studies of the methodology *sobriety checkpoints* found a 50% reduction in the prevalence of drinking and driving among drivers after the first dry law [51]. In São Paulo (Southeast Region), Campos et al. [52] found a 45% reduction in the prevalence of drivers with a positive breath test after the implementation of the first new dry law through the sobriety checkpoints methodology. Moura et al. [24], using data from Vigitel from 2007 to 2009, found that the frequency of driving after alcohol abuse dropped immediately after the first two months of the application of the first dry law (July and August), increasing again in September and October, reaching its peak levels in May 2009. These data suggest that Prohibition was not very effective in its first phase. Malta et al. [23], analyzing Vigitel data from 2007 to 2013, found that the prevalence of driving after alcohol abuse reduced immediately in the first year of application of first dry law in 2008 (Annual Percentage Change [APC]: -0.5%) and first year of application of the NDL in 2013 (APC: -0.5%). Measuring the impact of the BAC limits legislation allows observing the effectiveness/enforcement of the law, being essential for reducing the burden of injuries and deaths in traffic [51].

Although there was a reduction in the indicator in some capitals, there was no significant impact in many others. Even though the strategies and actions are well known, the legislation may not have been implemented as vigorously as is desirable in these capitals [53], contributing to this result. Therefore, the enhancement of inspection and enforcement of the law in all capitals is required, especially in those without evidence of policy impact.

This study has some limitations. Vigitel’s samples are exclusively sourced from the register of landlines in each capital [26]. Thus, the generalization of the results is limited, since the study only applies to the adult population residing in households covered by the landlines service [26]. The telephone network is not universal and may have low coverage in less developed cities and within families with lower economic status (BRAZIL, 2017), which may have underestimated the outcome prevalence being studied.

This investigation analyzed the impact of interventions only on the indicator “driving under the influence of alcohol abuse”. Although the increase in blood alcohol concentration increases the risk of RTI, the impact on the prevalence of driving after consumption of any amount of alcohol has not been analyzed [54], which also has significant impact on RTI morbidity and mortality. The indicator of the driving under the influence of any amount of alcohol was only collected by Vigitel as for 2011 [23]. Thus, there are not enough historical series points before the interventions to allow the monitoring and impact of the PLT and NDL on this indicator [23]. Other studies on the impacts of the programs should carry out the analysis of this indicator.

The outcome data analyzed were self-reported, a characteristic inherent to telephone surveys [55], being susceptible to memory and response bias. Memory biases, and especially response biases, can lead to underestimation of prevalence indicators, study outcomes. Thus, we do not rule out possible distortions in associations due to underreporting caused by memory and response bias of the dependent variable. Analyses of interventions in a stratified manner, in groups, according to sex or age group, could not be carried out since the outcome prevalence was too low for sensitivity analyses to be performed. Also, the present evaluation did not present a control group for the PLT (which did not undergo any intervention), since the Vigitel study is implemented exclusively in capital cities [26].

The number of time series points was 38, including three points with imputed data. Some studies suggest the need for at least 50 points for ITS analysis. Despite the limitations, this study represents the first one on the impact of PLT and NDL on the intermediate indicator of driving after alcohol abuse in Brazil, data that can support the strengthening of PLT legislation and activities.

## Conclusion

The present study identified an immediate impact of the PLT in two capitals (Belo Horizonte and Curitiba) and a joint impact of the NDL in 13 capitals. The results have implications for strengthening interventions and policies aimed at reducing the burden of morbidity and mortality from RTI in Brazil. The capitals, especially those with no significant impact, must intensify PLT actions.

Based on the results of this study, the systematic intensification of PLT actions, such as health education actions for drivers and society in general, inspection at sobriety checkpoints, media campaigns on the risks of drinking and driving, and implementation of data collection and periodic assessments of drinking and driving indicators in the capitals, is recommended. The increase in inspection and application of the NDL with periodic sobriety checkpoints is also recommended, especially in places with a high concentration of bars and nightclubs. Finally, other cross-sectional interventions, such as restriction of beverage sales hours, control of alcoholic beverage advertising, and prohibition of sales to minors, among others, can have an impact on reducing the prevalence of driving under the influence of alcohol and, consequently, on decreasing the RTI mortality rate.

Future investigations shall carry out quantitative and qualitative assessments of the PLT, including data on the degree of implementation of the program, details of the actions and the integrated plan and indicators, such as the number of sobriety checkpoints carried out. This information can support the understanding of the etiology of the program’s impacts on intermediate indicators, such as driving after alcohol consumption. The application of the NDL shall also be analyzed, including a systematic evaluation of indicators of the number of sobriety checkpoints and the number of drivers with a positive breath test. These indicators can support the in-depth assessment of laws in capital cities and explain the differences in their impacts across cities and regions in Brazil.

## Supporting information

S1 Table Analysis of the trend, stationarity, and seasonality of time series. (DOCX)

S2 Table Quality parameters of ARIMA or SARIMA models adjustment. (DOCX)

S3 Table Diagnostic tests of series autocorrelation and residuals normality of the final ARIMA or SARIMA models. (DOCX)

S1 File Descriptive analysis. (DOCX)

S2 File Decomposition of temporal series. (DOCX)

S3 File Temporal series ACF and PACF. (DOCX)

S4 File Residuals analysis. (DOCX)

## References

[1] World Health Organization. Global status report on road safety 2018. Geneva: WHO; 2018 [cited 24 Apr 2022]. Available: http://www.who.int/violence_injury_prevention/road_safety_status/2018/en/

[2] Ladeira RM, Malta DC, Neto OL de M, Montenegro M de MS, Filho AMS, Vasconcelos CH, et al. Road traffic accidents: Global Burden of Disease study, Brazil and federated units, 1990 and 2015. Rev Bras Epidemiol. 2017;20: 157–170. doi: 10.1590/1980-5497201700050013 28658380

[3] Instituto de Pesquisa Econômica Aplicada. Estimativa dos Custos dos Acidentes de Trânsito no Brasil com Base na Atualização Simplificada das Pesquisas Anteriores do Ipea: Relatório de Pesquisa. 2015 [cited 17 Sep 2019] pp. 1–20. Available: http://repositorio.ipea.gov.br/bitstream/11058/7456/1/RP_Estimativa_2015.pdf

[4] World Health Organization. Global Status Report on Alcohol and Health 2018. 2018 [cited 29 Jun 2022]. /entity/substance_abuse/publications/global_alcohol_report/en/index.html

[5] Cremonte M, Cherpitel CJ. Alcohol intake and risk of injury. Med (B Aires). 2014;74: 287–292. 25188654 PMC4161955

[6] Staton C, Vissoci J, Gong E, Toomey N, Wafula R, Abdelgadir J, et al. Road traffic injury prevention initiatives: A systematic review and metasummary of effectiveness in low and middle income countries. PLoS One. 2016;11: e0144971. doi: 10.1371/journal.pone.0144971 26735918 PMC4703343

[7] Hyder AA, Bishai D. Road Safety in 10 Countries: A Global Opportunity. Traffic Inj Prev. 2012;13: 1–2. doi: 10.1080/15389588.2011.650023 22414120

[8] World Health Organization. Road Safety in Ten Countries. 2014. Available: https://www.grsproadsafety.org/wp-content/uploads/RS-10-factsheet-V4-web.pdf

[9] Silva MMA, Morais Neto OL de, Lima CM de, Malta DC, Silva JB da Jr. Projeto Vida no Trânsito—2010 a 2012: uma contribuição para a década de ações para a segurança no trânsito 2011–2020 no Brasil. Epidemiol e Serviços Saúde. 2013;22: 531–536. doi: 10.5123/S1679-49742013000300019

[10] Gupta S, Paichadze N, Gritsenko E, Klyavin V, Yurasova E, Hyder AA. Evaluation of the five-year Bloomberg Philanthropies Global Road Safety Program in the Russian Federation. Public Health. 2017;144: S5–S14. doi: 10.1016/j.puhe.2016.12.030 28288732

[11] Morais Neto OL de, Silva MMA, Lima CM de, Malta DC, Silva JB da Jr. Projeto Vida no Trânsito: avaliação das ações em cinco capitais brasileiras, 2011–2012. Epidemiol e Serviços Saúde. 2013;22: 373–382. doi: 10.5123/S1679-49742013000300002

[12] Presidência da República, Casa Civil. Lei n° 9.503, de 23 de setembro de 1997. 1997 [cited 12 Nov 2019]. Available: http://www.planalto.gov.br/CCivil_03/leis/L9503.htm

[13] Presidência da República, Casa Civil. Lei 11.705, de 19 de Junho de 2008. 2008 [cited 10 Nov 2019]. Available: http://www.planalto.gov.br/ccivil_03/_ato2007-2010/2008/lei/l11705.htm

[14] Malta DC, Silva MMA da, Lima CM de, Soares Filho AM, Montenegro M de MS, Mascarenhas MDM, et al. Impacto da Legislação Restritiva do Álcool na Morbimortalidade por Acidentes de Transporte Terrestre—Brasil, 2008. Epidemiol Serviços Saúde. 2010;19: 77–78. doi: 10.5123/s1679-49742010000100009

[15] Presidência da República, Casa Civil. Lei n° 12.760, de 20 de dezembro de 2012. 2012. Available: https://www.planalto.gov.br/ccivil_03/_ato2011-2014/2012/lei/l12760.htm

[16] Presidência da República, Casa Civil. Lei n° 13.281, de 4 de maio de 2016. 2016 [cited 10 Nov 2019]. Available: http://www.planalto.gov.br/ccivil_03/_ato2015-2018/2016/lei/l13281.htm

[17] Chandran A, Pérez-Núñez R, Bachani AM, Híjar M, Salinas-Rodríguez A, Hyder AA. Early impact of a national multi-faceted road safety intervention program in Mexico: Results of a time-series analysis. PLoS One. 2014;9. doi: 10.1371/journal.pone.0087482 24498114 PMC3909119

[18] Nagata T, Setoguchi S, Hemenway D, Perry MJ. Effectiveness of a law to reduce alcohol-impaired driving in Japan. Inj Prev. 2008;14: 19–23. doi: 10.1136/ip.2007.015719 18245310

[19] Volpe FM, Ladeira RM, Fantoni R. Evaluating the Brazilian zero-tolerance drinking and driving law: Time series analyses of traffic-related mortality in three major cities. Traffic Inj Prev. 2017;18: 337–343. doi: 10.1080/15389588.2016.1214869 27588457

[20] Otero S, Rau T. The effects of drinking and driving laws on car crashes, injuries, and deaths: Evidence from Chile. Accid Anal Prev. 2017;106: 262–274. doi: 10.1016/j.aap.2017.05.031 28651146

[21] Guimarães AG, da Silva AR. Impact of regulations to control alcohol consumption by drivers: An assessment of reduction in fatal traffic accident numbers in the Federal District, Brazil. Accid Anal Prev. 2019;127: 110–117. doi: 10.1016/j.aap.2019.01.017 30851562

[22] Jomar RT, Ramos D de O, Fonseca VA de O, Junger WL. Effect of the zero-tolerance drinking and driving law on mortality due to road traffic accidents according to the type of victim, sex, and age in Rio de Janeiro, Brazil: An interrupted time series study. Traffic Inj Prev. 2019;20: 227–232. doi: 10.1080/15389588.2019.1576035 30985221

[23] Malta DC, Bernal RTI, da Silva MMA, Claro RM, da Silva Júnior JB, dos Reis AAC. Consumption of alcoholic beverages, driving vehicles, a balance of dry law, Brazil 2007–2013. Rev Saude Publica. 2014;48: 692–696. doi: 10.1590/s0034-8910.2014048005633 25210828 PMC4181103

[24] Moura EC, Malta DC, Morais Neto OL, Penna GO, Temporão JG. Direção de veículos motorizados após consumo abusivo de bebidas alcoólicas, Brasil, 2006 a 2009. Rev Saude Publica. 2009;43: 891–894. doi: 10.1590/S0034-89102009005000062 19784459

[25] Bernal RTI, Malta DC, Claro RM, Monteiro CA. Effect of the inclusion of mobile phone interviews to Vigitel. Rev Saude Publica. 2017;51: 15s. doi: 10.1590/S1518-8787.2017051000171 28591355 PMC5676392

[26] BRASIL. VIGITEL Brasil 2016 Vigilância de fatores de risco e proteção para doenças crônicas por inquérito telefônico. 2017. Available: http://portalarquivos.saude.gov.br/images/pdf/2017/junho/07/vigitel_2016_jun17.pdf 10.5123/S1679-49742017000400003 29211136

[27] Bernal RTI, Iser BPM, Malta DC, Claro RM. Surveillance System for Risk and Protective Factors for Chronic Diseases by Telephone Survey (Vigitel): changes in weighting methodology. Epidemiol e Serviços Saúde. 2017;26: 701–712. doi: 10.5123/s1679-49742017000400003 29211136

[28] Malta DC, Bernal RTI, Mascarenhas MDM, Silva MMA da, Szwarcwald CL, Morais Neto OL de. Alcohol consumption and driving in Brazilian capitals and Federal District according to two national health surveys. Rev Bras Epidemiol. 2015;18: 214–223. doi: 10.1590/1980-5497201500060019 27008616

[29] Box GEP, Jenkins GM. Time series analysis forecasting and control. San Francisco: Holden-Day; 1976.

[30] Sterne JAC, White IR, Carlin JB, Spratt M, Royston P, Kenward MG, et al. Multiple imputation for missing data in epidemiological and clinical research: potential and pitfalls. BMJ. 2009. doi: 10.1136/bmj.b2393 19564179 PMC2714692

[31] Bernal JL, Cummins S, Gasparrini A. Interrupted time series regression for the evaluation of public health interventions: a tutorial. Int J Epidemiol. 2017;46: 348–355. doi: 10.1093/ije/dyw098 27283160 PMC5407170

[32] Bhaskaran K, Gasparrini A, Hajat S, Smeeth L, Armstrong B. Time series regression studies in environmental epidemiology. Int J Epidemiol. 2013;42: 1187–1995. doi: 10.1093/ije/dyt092 23760528 PMC3780998

[33] Song X, Xiao J, Deng J, Kang Q, Zhang Y, Xu J. Time series analysis of influenza incidence in Chinese provinces from 2004 to 2011. Med. 2016;95: e3929. doi: 10.1097/MD.0000000000003929 27367989 PMC4937903

[34] Jebb AT, Tay L, Wang W, Huang Q. Time series analysis for psychological research: Examining and forecasting change. Front Psychol. 2015;6: 1–24. doi: 10.3389/fpsyg.2015.00727 26106341 PMC4460302

[35] Mann HB. Nonparametric Tests Against Trend. Econometrica. 1945;13: 245. doi: 10.2307/1907187

[36] Rutkowska A. Properties of the Cox–Stuart Test for Trend in Application to Hydrological Series: The Simulation Study. Commun Stat—Simul Comput. 2015;44: 565–579. doi: 10.1080/03610918.2013.784988

[37] Dickey DA, Hasza DP, Fuller WA. Testing for Unit Roots in Seasonal Time Series. J Am Stat Assoc. 1984;79: 355–367. doi: 10.2307/2288276

[38] Martelli F, Giacomozzi C, Fadda A, Frazzoli C. Understanding Seasonal Changes to Improve Good Practices in Livestock Management. Front Public Heal. 2018;6: 1–10. doi: 10.3389/fpubh.2018.00175 29963544 PMC6013551

[39] Cortes F, Turchi Martelli CM, Arraes de Alencar Ximenes R, Montarroyos UR, Siqueira Junior JB, Gonçalves Cruz O, et al. Time series analysis of dengue surveillance data in two Brazilian cities. Acta Trop. 2018;182: 190–197. doi: 10.1016/j.actatropica.2018.03.006 29545150

[40] Akaike H. A new look at the statistical model identification. IEEE Trans Autom Contr. 1974;19: 716–723.

[41] Razali NM, Wah YB. Power comparisons of Shapiro-Wilk, Kolmogorov-Smirnov, Lilliefors and Anderson-Darling tests. J Stat Model Anal. 2011;2: 21–33.

[42] N’Gattia AK, Coulibaly D, Nzussouo NT, Kadjo HA, Chérif D, Traoré Y, et al. Effects of climatological parameters in modeling and forecasting seasonal influenza transmission in Abidjan, Cote D’Ivoire. BMC Public Health. 2016;16: 1–7. doi: 10.1186/s12889-016-3503-1 27624302 PMC5022141

[43] Gitelman V, Hendel L, Carmel R, Bekhor S. An examination of the national road-safety programs in the ten world’s leading countries in road safety. Eur Transp Res Rev. 2012;4: 175–188. doi: 10.1007/s12544-012-0081-x

[44] Hughes BP, Anund A, Falkmer T. A comprehensive conceptual framework for road safety strategies. Accid Anal Prev. 2016;90: 13–28. doi: 10.1016/j.aap.2016.01.017 26890077

[45] Morais Neto OL, Andrade AL, Guimarães RA, Mandacarú PMP, Tobias GC. Regional disparities in road traffic injuries and their determinants in Brazil, 2013. Int J Equity Health. 2016;15: 142. doi: 10.1186/s12939-016-0433-6 27852263 PMC5112733

[46] Guimarães RA, Neto OLM. Prevalence and factors associated with driving under the influence of alcohol in Brazil: an analysis by macroregion. Int J Environ Res Public Health. 2020;17: 767. doi: 10.3390/ijerph17030767 31991757 PMC7037342

[47] Dellinger AM, Sleet DA. Preventing Traffi Injuries: Strategies That Work. Inj Prev. 2010;4: 82–89. doi: 10.1177/1559827609348694

[48] Voas RB, Tippetts AS, Fell JC. Assessing the effectiveness of minimum legal drinking age and zero tolerance laws in the United States. Accid Anal Prev. 2003;35: 579–587. doi: 10.1016/s0001-4575(02)00038-6 12729821

[49] Tsai Y, Wu S, Huang J, Kuo SCH, Rau C, Chien P, et al. The effect of lowering the legal blood alcohol concentration limit on driving under the influence (DUI) in southern Taiwan: a cross-sectional retrospective analysis. BMJ Open. 2019;9: e026481. doi: 10.1136/bmjopen-2018-026481 31005931 PMC6528014

[50] Andreuccetti G, Carvalho HB, Cherpitel CJ, Ye Y, Ponce JC, Kahn T, et al. Reducing the legal blood alcohol concentration limit for driving in developing countries: a time for change? Results and implications derived from a time–series analysis (2001–10) conducted in Brazil. Addiction. 2011;106: 2124–2131. doi: 10.1111/j.1360-0443.2011.03521.x 21631625 PMC3184361

[51] Salgado R de S, Campos VR, Duailibi S, Laranjeira RR. O impacto da “Lei Seca” sobre o beber e dirigir em Belo Horizonte/MG. Cien Saude Colet. 2012;17: 971–976. doi: 10.1590/s1413-81232012000400019 22534851

[52] Campos VR, Silva RDSE, Duailibi S, Santos JF Dos, Laranjeira R, Pinsky I. The effect of the new traffic law on drinking and driving in São Paulo, Brazil. Accid Anal Prev. 2013;50: 622–627. doi: 10.1016/j.aap.2012.06.011 22818353

[53] Sweedler BM, Stewart K. Worldwide trends in alcohol and drug impaired driving. 2009. 10.1007/978-3-7643-9923-8_2 15276918

[54] Macinko J, Mullachery P, Silver D, Jimenez G, Morais Neto OL. Patterns of alcohol consumption and related behaviors in Brazil: evidence from the 2013 National Health Survey (PNS 2013). PLoS One. 2015;10: e0134153. doi: 10.1371/journal.pone.0134153 26230389 PMC4521809

[55] Matozinhos FP, Felisbino-Mendes MS, Gomes CS, Jansen AK, Machado ÍE, Lana FCF, et al. Cardiovascular health in Brazilian state capitals. Rev Lat Am Enfermagem. 2017;25: e2971. 29069270 10.1590/1518-8345.1327.2843 PMC5656337

