# Supplementary material for: Impact of the program life in traffic and new zero-tolerance drinking and driving law on the prevalence of driving after alcohol abuse in Brazilian capitals: An interrupted time series analysis
Source: PLoS One. 2023 Oct 20;18(10):e0288288. doi: 10.1371/journal.pone.0288288 (PMC10588900; doi:10.1371/journal.pone.0288288)
Supplement: S1 Table — (DOCX) [file pone.0288288.s001.docx]

**Table S1**. Analysis of the trend, stationarity, and seasonality of time series

| **City** | **Trend** | **Stationary** | **Stable seasonality** | | | | **Mobile seasonality** | |
| --- | --- | --- | --- | --- | --- | --- | --- | --- |
|  | **Mann-Kendall** | **Dickey-Fuller test*** | **Kruskall-Wallis test** | | **1-way ANOVA** | | **2-way ANOVA** | |
|  | **Z (p-valor)** | **t (p-value) [lag]** | **χ^2^** | **p-value** | ***F*** | **p-value** | ***F*** | **p-value** |
| Aracaju | **-2.679 (0.007)** | -2.292 (0.438) [3] | 2.236 | 0.525 | 0.657 | 0.584 | 0.295 | 0.969 |
| Belém | -0.604 (0.546) | -7.143 (<0.001) [1] | 1.900 | 0.593 | 0.606 | 0.616 | 0.630 | 0.761 |
| Belo Horizonte | **-4.566 (< 0.001)** | -3.480 (0.042) [2] | 0.626 | 0.891 | 0.417 | 0.742 | 0.137 | 0.998 |
| Boa Vista | **-2.037 (0.041)** | -6.685 (<0.001) [0] | 2.037 | 0.565 | 0.794 | 0.506 | 0.191 | 0.993 |
| Campo Grande | -1.067 (0.284) | -4.654 (<0.001) [0] | 1.278 | 0.734 | 0.245 | 0.864 | 1.516 | 0.196 |
| Cuiabá | -0.956 (0.339) | -5.054 (<0.001) [0] | 2.530 | 0.470 | 0.821 | 0.491 | 0.825 | 0.599 |
| Curitiba | -1.559 (0.119) | -3.160 (0.022) [3] | 4.716 | 0.194 | 1.807 | 0.164 | 0.987 | 0.475 |
| Florianópolis | -1.648 (0.099) | -5.430 (<0.001) [0] | 3.329 | 0.344 | 0.938 | 0.433 | 1.293 | 0.289 |
| Fortaleza | -2.265 (0.023) | -3.435 (0.047) [1] | 2.142 | 0.544 | 0.789 | 0.509 | 0.155 | 0.997 |
| Goiânia | -0.893 (0.372) | -6.727 (<0.001) [0] | 14.278 | **0.003** | 6.856 | **0.001** | 0.952 | 0.500 |
| João Pessoa | **-4.101 (< 0.001)** | -3.096 (0.027) [1] | 3.732 | 0.292 | 1.291 | 0.294 | 0.299 | 0.987 |
| Macapá | -1.799 (0.072) | -2.954 (0.003) [1] | 5.889 | 0.117 | 2.784 | 0.056 | 0.261 | 0.979 |
| Maceió | **-2.799 (0.001)** | -1.850 (0.356) [3] | 6.185 | 0.103 | 2.393 | 0.086 | 0.111 | 0.999 |
| Manaus | **-2.327 (0.019)** | -2.661 (0.081) [3] | 12.388 | **0.006** | 5.664 | **0.003** | 0.203 | 0.991 |
| Natal | **-3.233 (0.001)** | -1.690 (0.436) [2] | 1.529 | 0.676 | 0.563 | 0.643 | 0.166 | 0.996 |
| Palmas | **-2.377 (0.017)** | -4.706 (<0.001) [0] | 0.548 | 0.908 | 0.189 | 0.903 | 0.540 | 0.831 |
| Porto Alegre | -1.811 (0.070) | -6.027 (<0.001) [0] | 2.516 | 0.472 | 1.200 | 0.325 | 1.299 | 0.286 |
| Porto Velho | **-3.433 (0.005)** | -3.058 (0.030) [1] | 6.602 | 0.086 | 2.939 | **0.048** | 0.592 | 0.791 |
| Recife | **-3.423 (0.006)** | -1.676 (0.444) [3] | 3.236 | 0.357 | 1.028 | 0.393 | 0.887 | 0.551 |
| Rio Branco | **-2.364 (0.018)** | -1.714 (0.424) [3] | 10.818 | **0.013** | 3.186 | **0.036** | 0.118 | 0.999 |
| Rio de Janeiro | **-2.540 (0.011)** | -2.484 (0.119) [2] | 2.045 | 0.563 | 0.544 | 0.656 | 0.035 | 1.000 |
| Salvador | **-2.844 (0.004)** | -3.381 (0.054) [1] | 0.671 | 0.880 | 0.455 | 0.715 | 1.027 | 0.447 |
| São Luís | -1.054 (0.291) | -5.024 (<0.001) [0] | 0.920 | 0.821 | 0.370 | 0.775 | 1.632 | 0.160 |
| São Paulo | -1.119 (0.263) | -2.806 (0.057) [7]e | 6.840 | 0.077 | 2.941 | **0.047** | 1.470 | 0.213 |
| Teresina | **-2.440 (0.015)** | **-4.559 (<0.001) [0]** | 1.330 | 0.722 | 0.380 | 0.768 | 0.128 | 0.998 |
| Vitória | **-2.314 (0.020)** | -5.299 (<0.001) [0] | 2.544 | 0.467 | 1.152 | 0.343 | 0.754 | 0.658 |
| Brasília | 0.213 (0.830) | -6.382 (<0.001) [0] | 2.176 | 0.537 | 0.568 | 0.640 | 1.792 | 0.120 |

**Abbreviations:** 95.0% CI: 95.0% Confidence Interval; *Number of lag chosen by the AIC criterion.
