# Supplementary material for: Impact of the program life in traffic and new zero-tolerance drinking and driving law on the prevalence of driving after alcohol abuse in Brazilian capitals: An interrupted time series analysis
Source: PLoS One. 2023 Oct 20;18(10):e0288288. doi: 10.1371/journal.pone.0288288 (PMC10588900; doi:10.1371/journal.pone.0288288)
Supplement: S2 Table — (DOCX) [file pone.0288288.s002.docx]

**Table S2**. Quality parameters of ARIMA or SARIMA models adjustment

| **City** | **Model** | ***Log likelihood*** | **AIC** | **AICC** | **BIC** | **ME** | **RMSE** | **MAE** | **MPE** | **MAPE** | **MASE** |
| --- | --- | --- | --- | --- | --- | --- | --- | --- | --- | --- | --- |
| Aracaju | (0,1,1) | -52.470 | 110.949 | 111.302 | 115.782 | 0.042 | 0.939 | 0.704 | -40.909 | 62.096 | 0.631 |
| Belém | (1,0,1) | -32,750 | 75.507 | 76.720 | 83.695 | 0.041 | 0.556 | 0.436 | * | * | 0.546 |
| Belo Horizonte | (4,1,2) | -31.970 | 81.949 | 87.092 | 96.447 | 0.012 | 0.493 | 0.389 | * | * | 0.480 |
| Boa Vista | (1,0,1) | -47.370 | 104.739 | 112.927 | 105.951 | 0.000 | 0.816 | 0.604 | -13.433 | 29.887 | 0.640 |
| Campo Grande | (0,0,2) | -45.680 | 103.362 | 105.237 | 113.118 | -0.033 | 0.801 | 0.647 | -36.799 | 58.242 | 0.697 |
| Cuiabá | (0,0,1) | -49.200 | 106.042 | 106.748 | 112.592 | 0.009 | 0.878 | 0.642 | * | * | 0.529 |
| Curitiba | (1,0,1) | -31.840 | 75.686 | 77.561 | 85.511 | 0.059 | 0.537 | 0.444 | -20.349 | 44.221 | 0.543 |
| Florianópolis | (0,0,0) | -47.850 | 101.709 | 102.052 | 106.622 | 0.000 | 0.852 | 0.685 | -20.116 | 41.283 | 0.769 |
| Fortaleza | (0,1,1) | -48.100 | 102.193 | 102.546 | 107.026 | 0.037 | 0.834 | 0.642 | -39.573 | 64.920 | 0.679 |
| Goiânia | (1,0,1) (1,0,0) [4] | -51.590 | 115.187 | 117.061 | 125.012 | -0.051 | 0.895 | 0.710 | * | * | 0.507 |
| João Pessoa | (4,1,2) | -43.230 | 102.460 | 106.322 | 115.348 | -0.172 | 0.702 | 0.532 | * | * | 0.531 |
| Macapá | (1,1,0) | -54.960 | 115.923 | 116.279 | 120.759 | -0.021 | 1.047 | 0,808 | -37.876 | 67.414 | 0.720 |
| Maceió | (3,1,3) | -36.640 | 89.286 | 93.148 | 102.173 | -0.117 | 0.576 | 0.452 | * | * | 0.507 |
| Manaus | (2,1,2) (1,0,0) [4] | -36.750 | 87.491 | 90.291 | 98.767 | -0.062 | 0.607 | 0.459 | * | * | 0.540 |
| Natal | (1,1,3) | -46.080 | 104.156 | 106.091 | 113.822 | -0.046 | 0.747 | 0.577 | * | * | 0.645 |
| Palmas | (1,1,1) | -49.05 | 108.104 | 109.355 | 116.159 | -0.033 | 0.855 | 0.699 | -9.643 | 25.831 | 0.666 |
| Porto Alegre | (1,0,3) | -33.830 | 81.659 | 84.366 | 93.120 | 0.033 | 0.532 | 0.402 | -34.492 | 65.608 | 0.498 |
| Porto Velho | (1,1,1) (0,1,1) [4] | -37.000 | 83.996 | 85.424 | 91.478 | 0.025 | 0.599 | 0.444 | -14.161 | 33.119 | 0.757 |
| Recife | (0,1,1) | -37.580 | 81.153 | 81.506 | 85.986 | 0.003 | 0.628 | 0.541 | * | * | 0.739 |
| Rio Branco | (0,1,3) (3,1,1) [4] | -39.770 | 97.545 | 103.54 | 111.014 | 0.169 | 0.587 | 0.417 | -13.374 | 57.726 | 0.479 |
| Rio de Janeiro | (3,1,0) | -39.210 | 88.429 | 89.679 | 96.484 | -0.054 | 0.676 | 0.503 | * | * | 0.663 |
| Salvador | (0,1,4) | -42.500 | 96.997 | 98.933 | 106.663 | -0.140 | 0.715 | 0.547 | * | * | 0.783 |
| São Luís | (3,0,0) | -48.530 | 109.069 | 110.945 | 118.895 | 0.012 | 0.860 | 0.656 | -16.763 | 37.869 | 0.638 |
| São Paulo | (2,0,1) (1,0,0) [4] | -25.320 | 62.643 | 64.518 | 72.468 | 0.004 | 0.466 | 0.383 | -51.135 | 77.174 | 0.528 |
| Teresina | (1,1,1) | -52.590 | 115.184 | 116.434 | 123.239 | -0.117 | 0.941 | 0.705 | -20.059 | 34.269 | 0.624 |
| Vitória | (0,1,1) | -40.99 | 87.985 | 88.337 | 92.818 | -0.223 | 0.717 | 0.554 | * | * | 0.770 |
| Brasília | (0,0,0) | -51.150 | 108.299 | 108.642 | 113.212 | 0.000 | 0.929 | 0.730 | -20.536 | 41.580 | 0.796 |

**Abbreviations:** ME: mean error; RMSE: root mean squared error; MAE: mean absolute error; MPE: mean percentage error; MAPE: Mean absolute percentage error; MASE: Mean absolute scaled error; *Undefined due to the presence of zeros in the sample.
