# Supplementary material for: Impact of the program life in traffic and new zero-tolerance drinking and driving law on the prevalence of driving after alcohol abuse in Brazilian capitals: An interrupted time series analysis
Source: PLoS One. 2023 Oct 20;18(10):e0288288. doi: 10.1371/journal.pone.0288288 (PMC10588900; doi:10.1371/journal.pone.0288288)
Supplement: S3 Table — (DOCX) [file pone.0288288.s003.docx]

**Table S3**. Diagnostic tests of series autocorrelation and residuals normality of the final ARIMA or SARIMA models

| **City** | **Model** | **Ljung-Box test*** | | **K-S test**** | | **AD test**** | | **SW test**** | |
| --- | --- | --- | --- | --- | --- | --- | --- | --- | --- |
|  |  | **Q (lag 10)** | **p-value** | **D** | **p-value** | **A** | **p-value** | **W** | **p-value** |
| Aracaju | (0,1,1) | 6.226 | 0.622 | 0.145 | 0.330 | 0.662 | 0.097 | 0.963 | 0.243 |
| Belém | (1,0,1) | 6.473 | 0.372 | 0.183 | 0.137 | 0.234 | 0.780 | 0.977 | 0.622 |
| Belo Horizonte | (4,1,2) | 4.414 | 0.936 | 0.212 | 0.056 | 0.156 | 0.950 | 0.989 | 0.971 |
| Boa Vista | (1,0,1) | 8.445 | 0.207 | 0.158 | 0.265 | 0.523 | 0.172 | 0.953 | 0.108 |
| Campo Grande | (0,0,2) | 4.944 | 0.423 | 0.090 | 0.887 | 0.983 | 0.829 | 0.158 | 0.946 |
| Cuiabá | (0,0,1) | 10.439 | 0.165 | 0.117 | 0.214 | 0.662 | 0.077 | 0.956 | 0.144 |
| Curitiba | (1,0,1) | 5.308 | 0.379 | 0.229 | **0.030** | 0.343 | 0.471 | 0.963 | 0.252 |
| Florianópolis | (0,0,0) | 8.284 | 0.406 | 0.084 | 0.947 | 0.192 | 0.889 | 0.983 | 0.833 |
| Fortaleza | (0,1,1) | 10.663 | 0.221 | 0.129 | 0.552 | 0.597 | 0.113 | 0.942 | **0.049** |
| Goiânia | (0,0,0) (1,0,2) [4] | 6.744 | 0.238 | 0.134 | 0.458 | 0.267 | 0.667 | 0.985 | 0.892 |
| João Pessoa | (4,1,2) | 5.878 | 0.118 | 0.222 | 0.040 | 0.211 | 0.517 | 0.978 | 0.652 |
| Macapá | (1,1,0) | 8.408 | 0.394 | 0.081 | 0.945 | 0.334 | 0.469 | 0.972 | 0.424 |
| Maceió | (3,1,3) | 4.711 | 0.194 | 0.215 | 0.050 | 0.208 | 0.856 | 0.984 | 0.850 |
| Manaus | (2,1,2) (1,0,0) [4] | 6.102 | 0.192 | 0.184 | 0.133 | 0.641 | 0.087 | 0.947 | 0.073 |
| Natal | (1,1,3) | 2.860 | 0.722 | 0.143 | 0.388 | 0.248 | 0.734 | 0.248 | 0.903 |
| Palmas | (1,1,1) | 4.771 | 0.573 | 0.099 | 0.813 | 0.257 | 0.701 | 0.977 | 0.602 |
| Porto Alegre | (1,0,3) | 7.017 | 0.135 | 0.201 | 0.080 | 0.239 | 0.764 | 0.986 | 0.807 |
| Porto Velho | (1,1,1) (0,1,1) [4] | 11.488 | 0.075 | 0.184 | 0.132 | 0.204 | 0.864 | 0.986 | 0.906 |
| Recife | (0,1,1) | 13.064 | 0.101 | 0.180 | 0.151 | 0.612 | 0.103 | 0.951 | 0.100 |
| Rio Branco | (0,1,3) (3,1,1) [4] | 4.904 | 0.086 | 0.273 | **0.003** | 0.599 | 0.111 | 0.948 | 0.074 |
| Rio de Janeiro | (3,1,0) | 3.425 | 0.754 | 0.171 | 0.194 | 0.307 | 0.547 | 0.970 | 0.401 |
| Salvador | (0,1,4) | 6.935 | 0.225 | 0.221 | **0.040** | 0.579 | 0.122 | 0.948 | 0.080 |
| São Luís | (3,0,0) | 5.845 | 0.321 | 0.134 | 0.548 | 0.457 | 0.251 | 0.954 | 0.126 |
| São Paulo | (2,0,0) (1,0,0) [4] | 6.468 | 0.262 | 0;236 | **0.024** | 0.947 | 0.069 | 0.507 | 0.189 |
| Teresina | (1,1,1) | 3.898 | 0.690 | 0.135 | 0.449 | 0.326 | 0.509 | 0.979 | 0.669 |
| Vitória | (0,1,1) | 6.868 | 0.551 | 0.222 | **0.039** | 0.334 | 0.468 | 0.955 | 0.125 |
| Brasília | (0,0,0) | 4.338 | 0.825 | 0.135 | 0.495 | 0.639 | 0.099 | 0.941 | **0.046** |

**Abbreviations:** K-S: Kolmogorov-Smirnov test; AD: Anderson-Darling test; S-W: Shapiro-Wilk test; *Autocorrelation analysis test of the models residuals; **Test for models residuals normality.
