# Supplementary material for: Impact of the program life in traffic and new zero-tolerance drinking and driving law on the prevalence of driving after alcohol abuse in Brazilian capitals: An interrupted time series analysis
Source: PLoS One. 2023 Oct 20;18(10):e0288288. doi: 10.1371/journal.pone.0288288 (PMC10588900; doi:10.1371/journal.pone.0288288)
Supplement: S1 File — (DOCX) [file pone.0288288.s004.docx]

**File S1**. Descriptive analysis

Figs 1 to 22 of the Appendix show the descriptive analysis of prevalence of driving after abusive use of alcohol in 22 city capitals.

**Southeast macro-region**


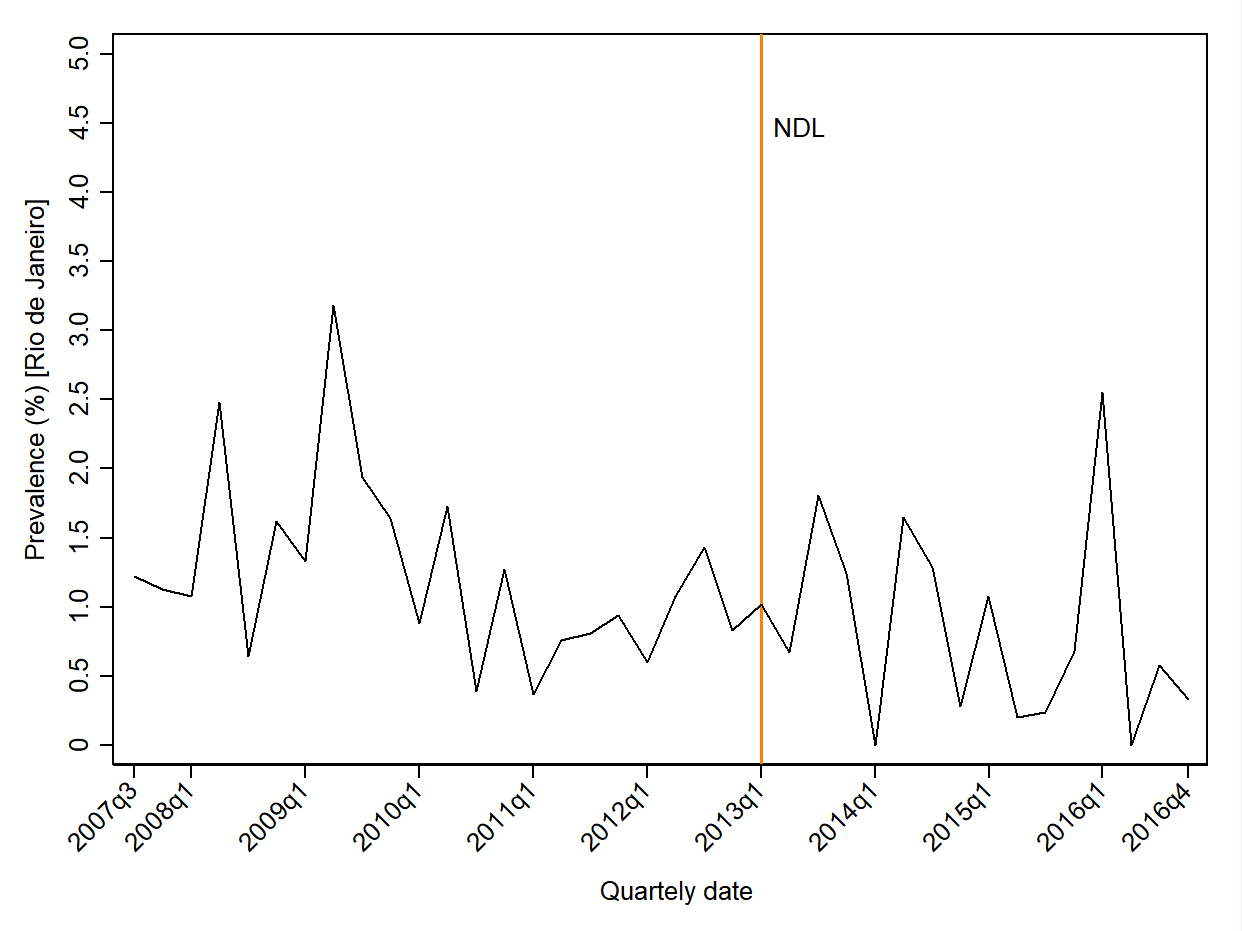


**Fig 1. Prevalence of drinking and driving in the city of Rio de Janeiro City (State of Rio de Janeiro), 2007q3 to 2016q4.** Abbreviations: NDL: New Dry Law


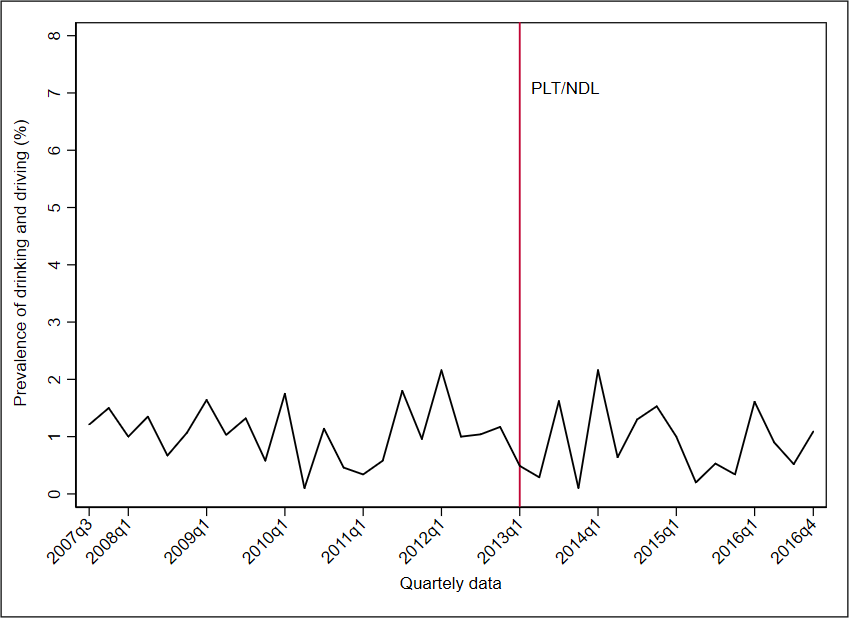


**Fig 2. Prevalence of drinking and driving in the city of São Paulo (State of São Paulo), 2007q3 to 2016q4.** Abbreviations: PLT: Program Life in Traffic; NDL: New Dry Law


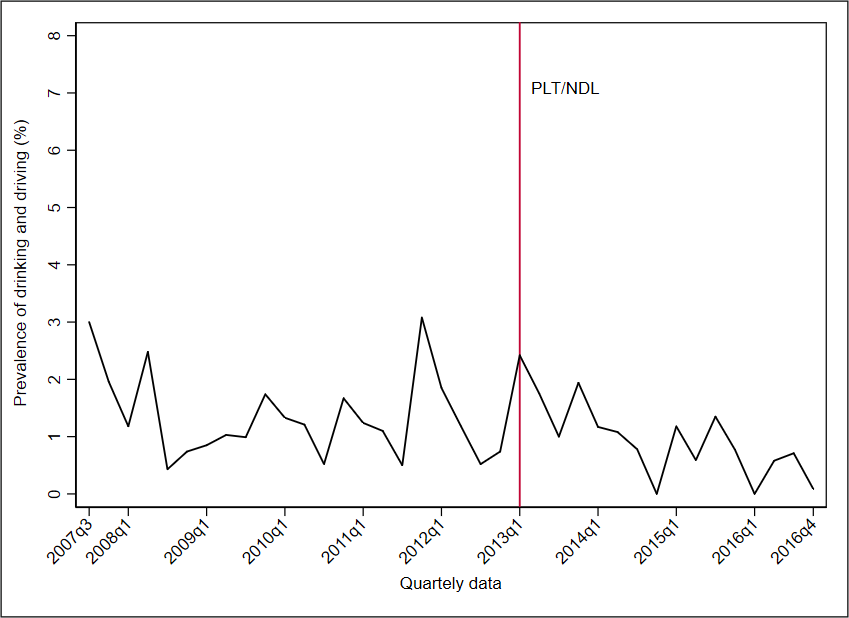


**Fig 3. Prevalence of drinking and driving in the city of Vitória (State of Espírito Santo), 2007q3 to 2016q4.** Abbreviations: PLT: Program Life in Traffic; NDL: New Dry Law

**South macro-region**

**
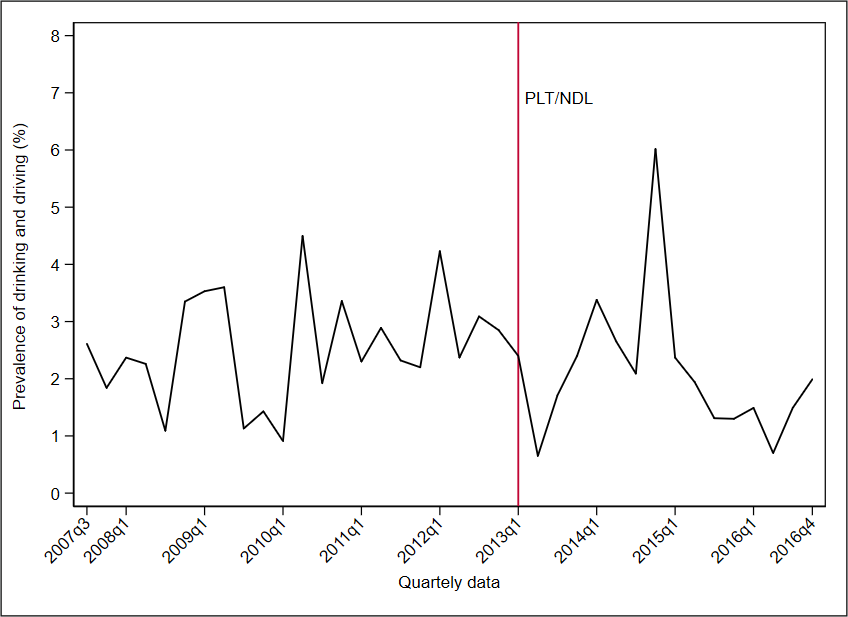
**

**Fig 4. Prevalence of drinking and driving in the city of Florianópolis (State of Santa Catarina), 2007q3 to 2016q4.** Abbreviations: PLT: Program Life in Traffic; NDL: New Dry Law


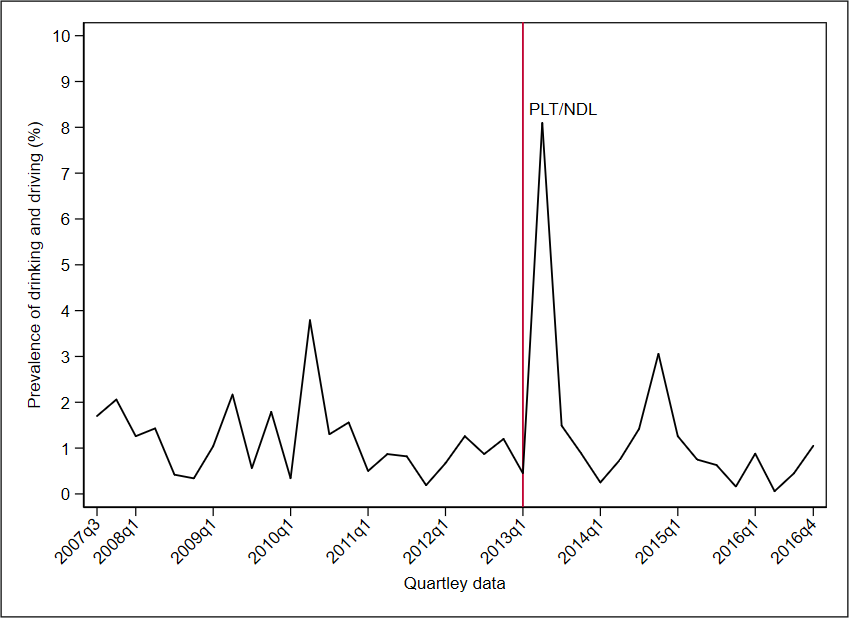


**Fig 5. Prevalence of drinking and driving in the city of Porto Alegre (State of Rio Grande do Sul), 2007q3 to 2016q4.** Abbreviations: PLT: Program Life in Traffic; NDL: New Dry Law

**Midwest macro-region**


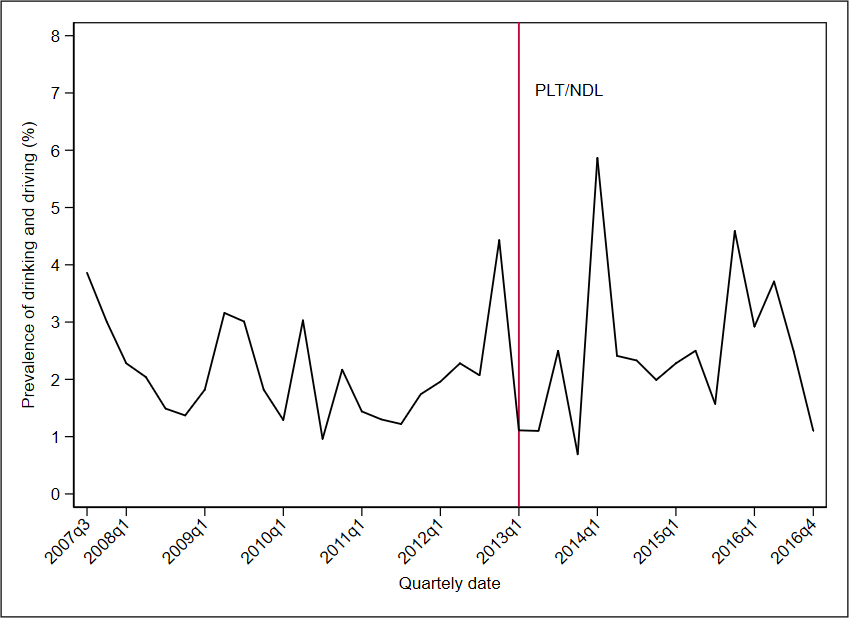


**Fig 6. Prevalence of drinking and driving in Brasília (Distrito Federal), 2007q3 to 2016q4.** Abbreviations: PLT: Program Life in Traffic; NDL: New Dry Law


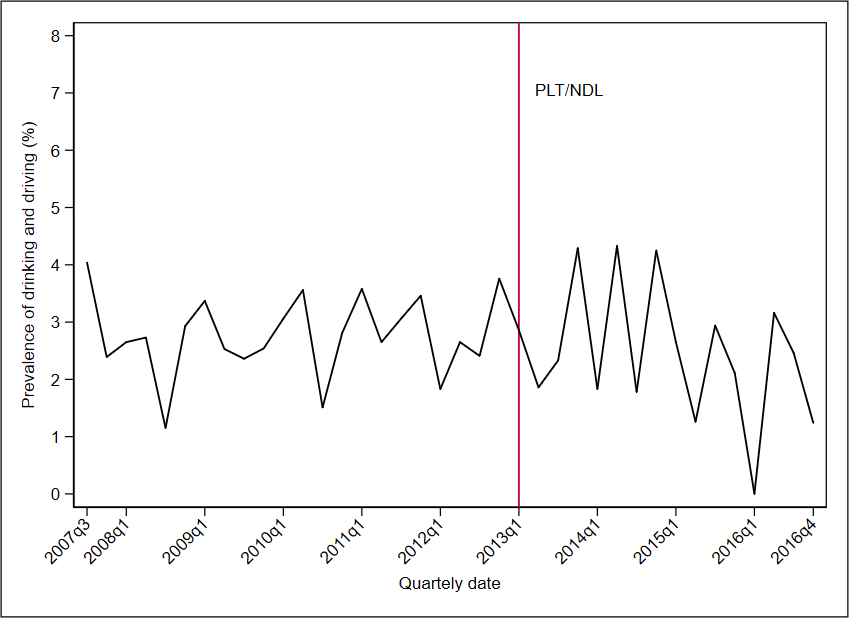


**Fig 7. Prevalence of drinking and driving in the city of Cuiabá (State of Mato Grosso), 2007q3 to 2016q4.** Abbreviations: PLT: Program Life in Traffic; NDL: New Dry Law


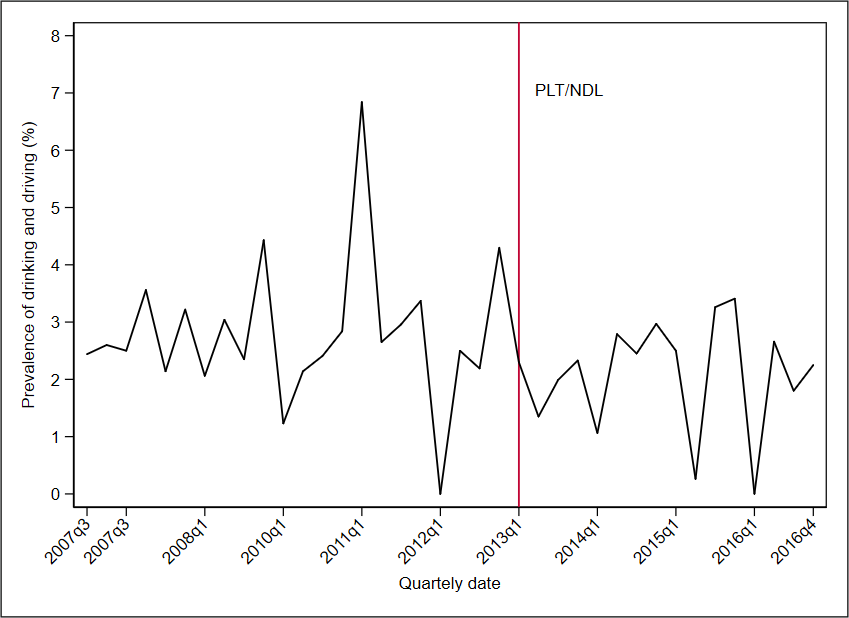


**Fig 8. Prevalence of drinking and driving in the city of Goiânia (State of Goiás), 2007q3 to 2016q4.** Abbreviations: PLT: Program Life in Traffic; NDL: New Dry Law

**North macro-region**


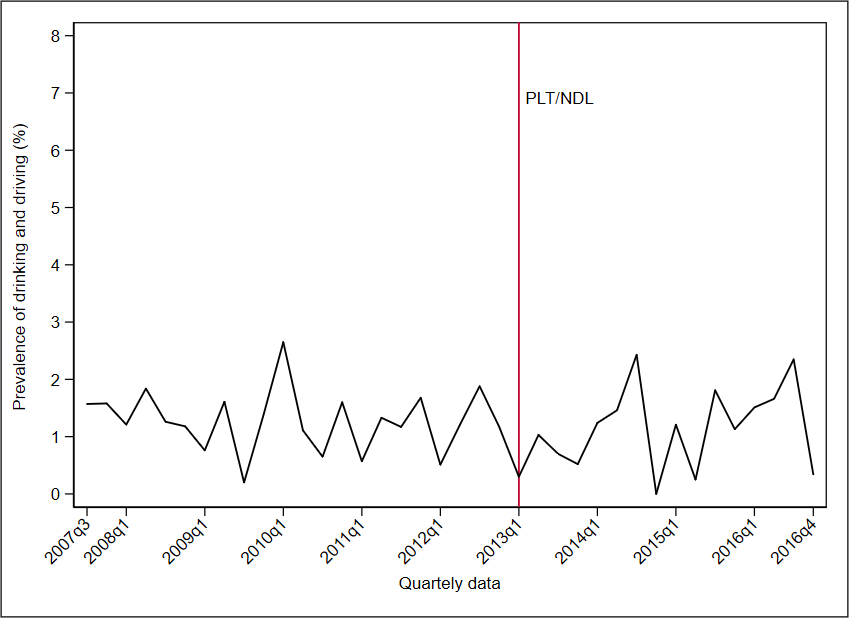


**Fig 9. Prevalence of drinking and driving in the city of Belém (State of Pará), 2007q3 to 2016q4.** Abbreviations: PLT: Program Life in Traffic; NDL: New Dry Law


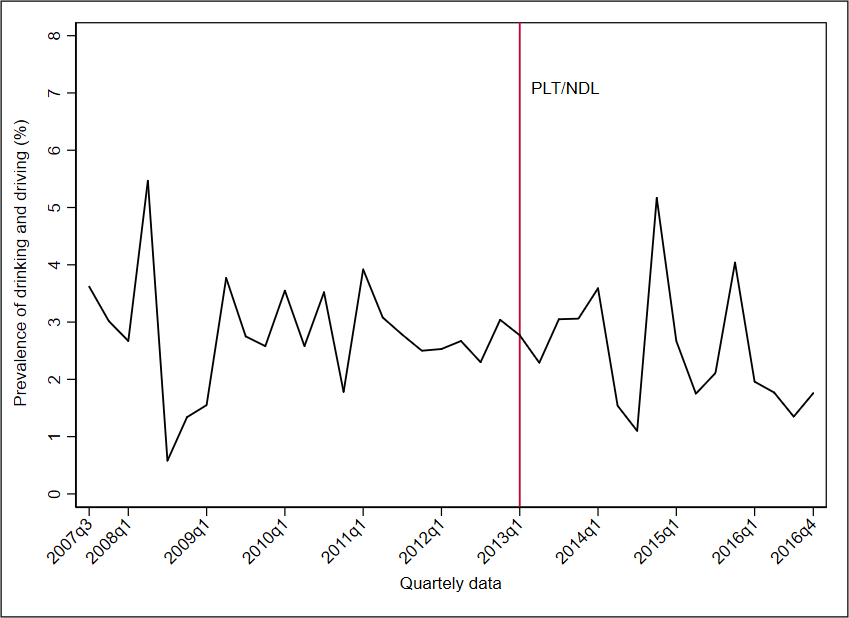


**Fig 10. Prevalence of drinking and driving in the city of Boa Vista (state of Roraima), 2007q3 to 2016q4.** Abbreviations: PLT: Program Life in Traffic; NDL: New Dry Law


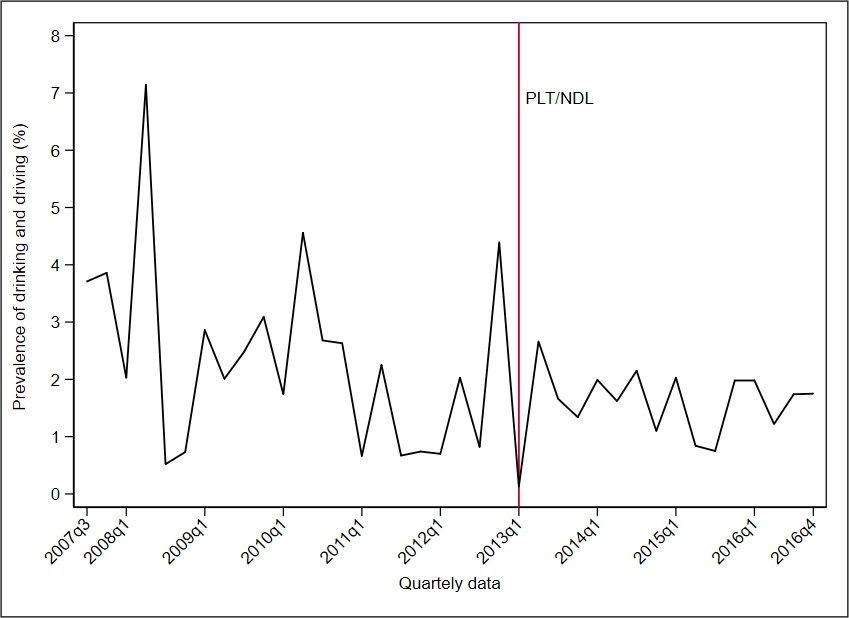


**Fig 11. Prevalence of drinking and driving in the city of Macapá (state of Amapá), 2007q3 to 2016q4.** Abbreviations: PLT: Program Life in Traffic; NDL: New Dry Law


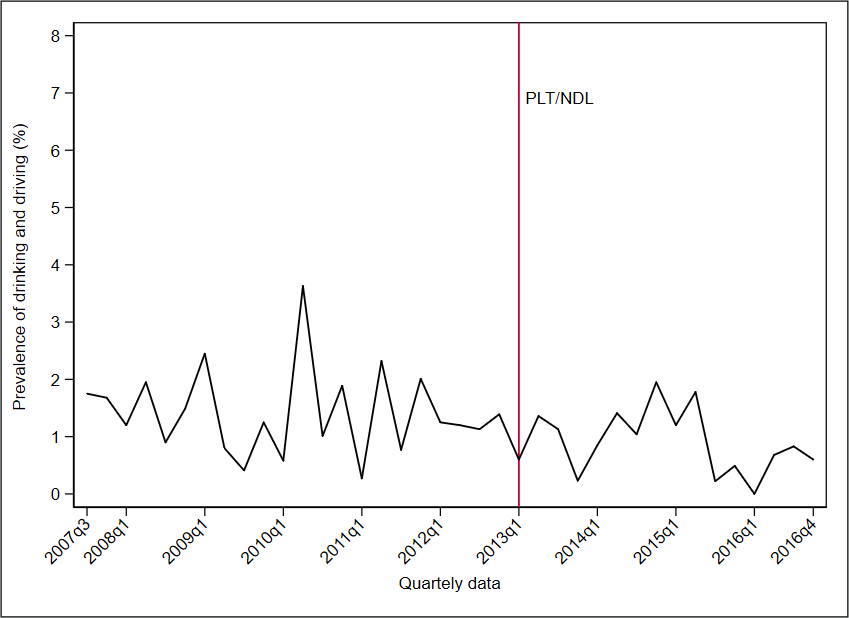


**Fig 12. Prevalence of drinking and driving in the city of Manaus (state of Amazonas), 2007q3 to 2016q4.** Abbreviations: PLT: Program Life in Traffic; NDL: New Dry Law


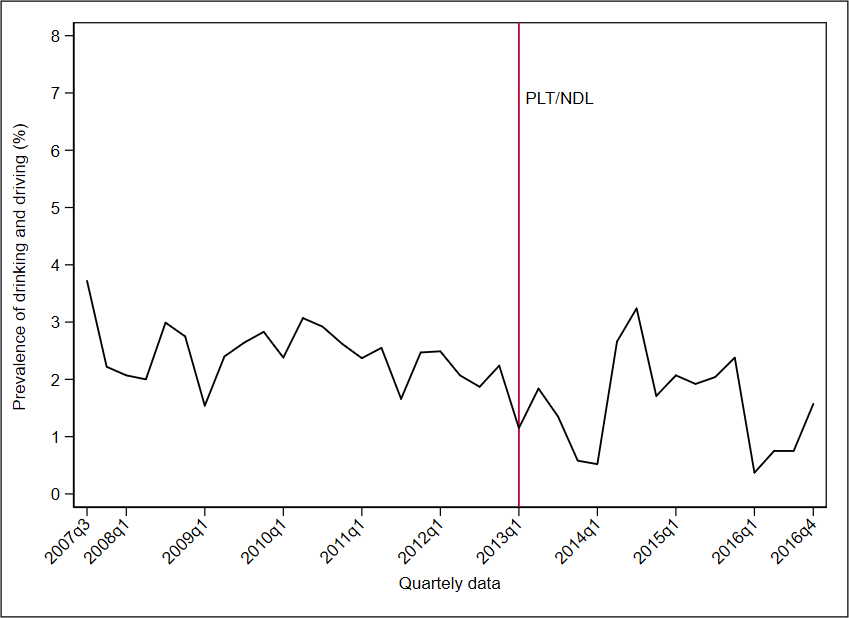


**Fig 13. Prevalence of drinking and driving in the city of Porto Velho (state of Rondônia), 2007q3 to 2016q4.** Abbreviations: PLT: Program Life in Traffic; NDL: New Dry Law


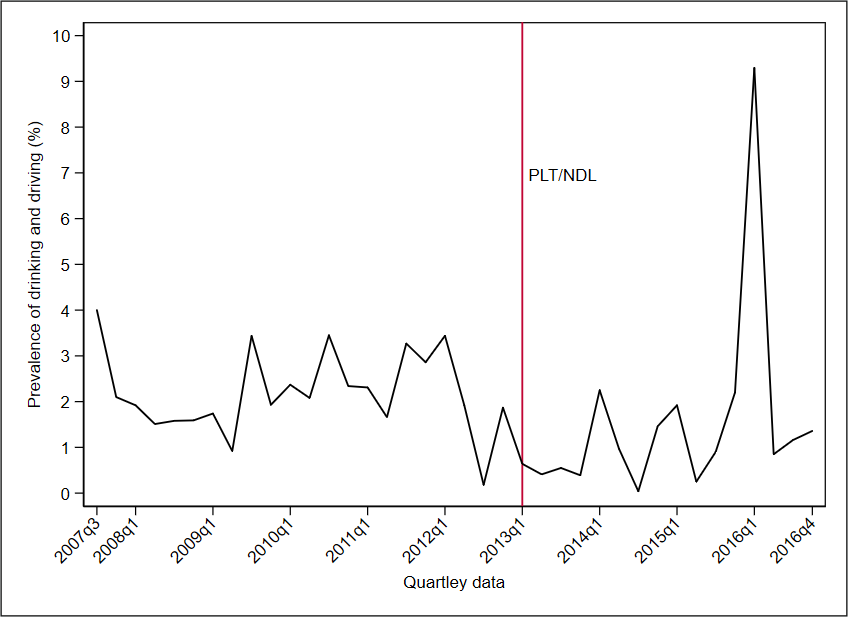


**Fig 14. Prevalence of drinking and driving in the city of Rio Branco (state of Acre), 2007q3 to 2016q4.** Abbreviations: PLT: Program Life in Traffic; NDL: New Dry Law

**Northeast macro-region**


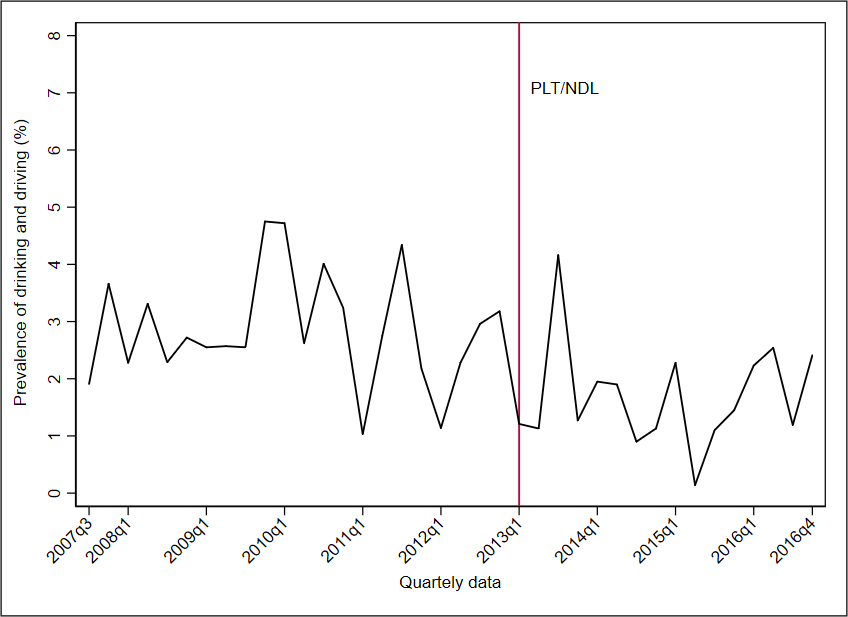


**Fig 15. Prevalence of drinking and driving in the city of Aracaju (State of Sergipe), 2007q3 to 2016q4.** Abbreviations: PLT: Program Life in Traffic; NDL: New Dry Law


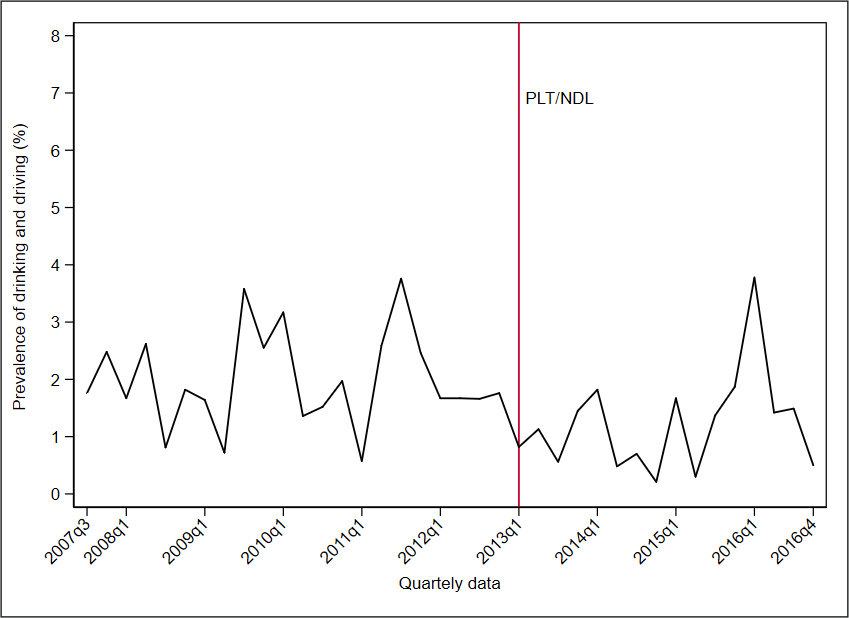


**Fig 16. Prevalence of drinking and driving in the city of Fortaleza (state of Ceará), 2007q3 to 2016q4.** Abbreviations: PLT: Program Life in Traffic; NDL: New Dry Law


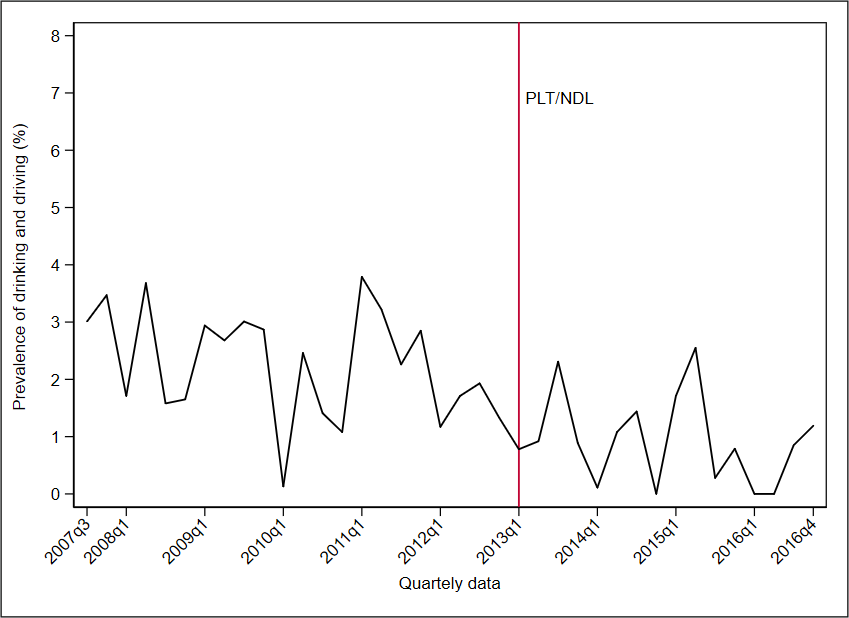


**Fig 17. Prevalence of drinking and driving in the city of João Pessoa (State of Paraíba), 2007q3 to 2016q4.** Abbreviations: PLT: Program Life in Traffic; NDL: New Dry Law


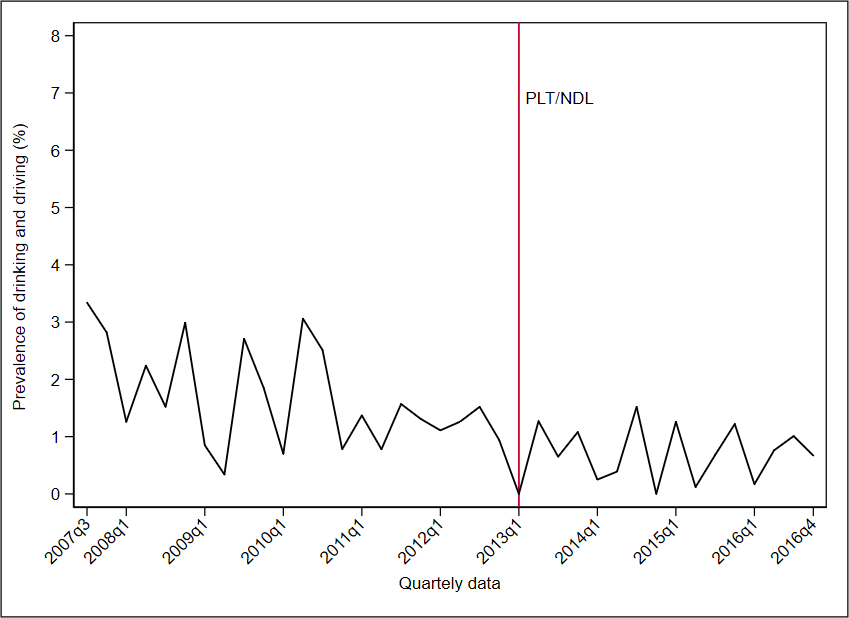


**Fig 18. Prevalence of drinking and driving in the city of Maceió (state of Alagoas), 2007q3 to 2016q4.** Abbreviations: PLT: Program Life in Traffic; NDL: New Dry Law


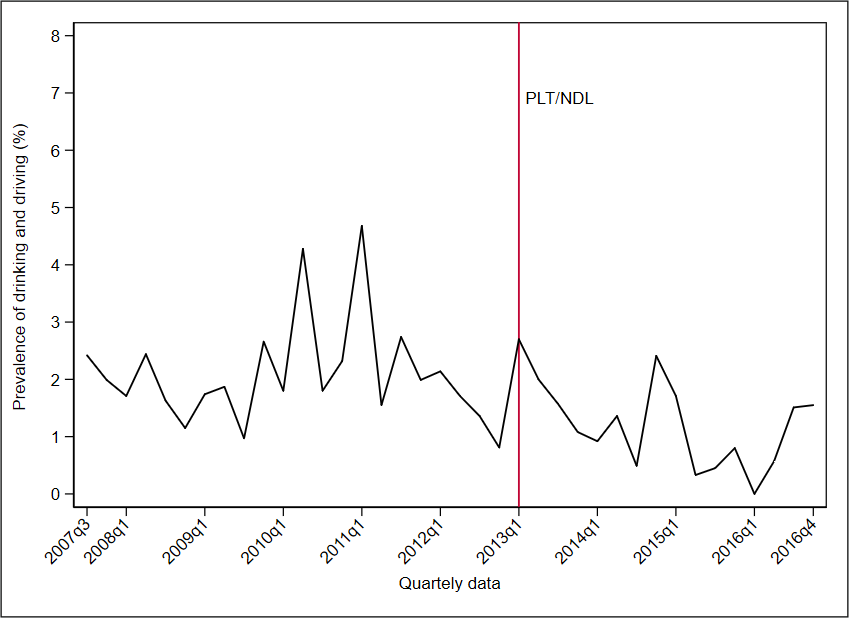


**Fig 19. Prevalence of drinking and driving in the city of Natal (state of Rio Grande do Norte), 2007q3 to 2016q4.** Abbreviations: PLT: Program Life in Traffic; NDL: New Dry Law


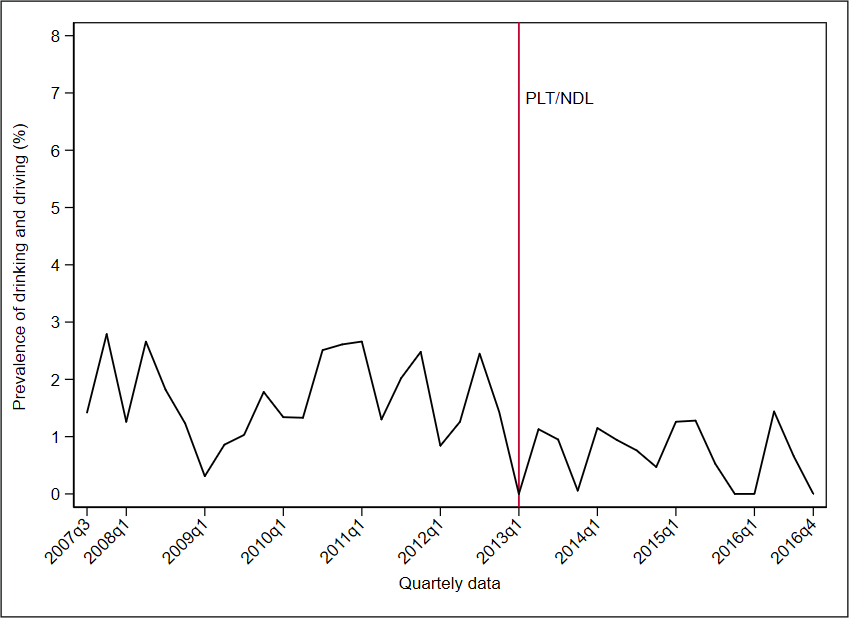


**Fig 20. Prevalence of drinking and driving in the city of Recife (state of Pernambuco), 2007q3 to 2016q4.** Abbreviations: PLT: Program Life in Traffic; NDL: New Dry Law


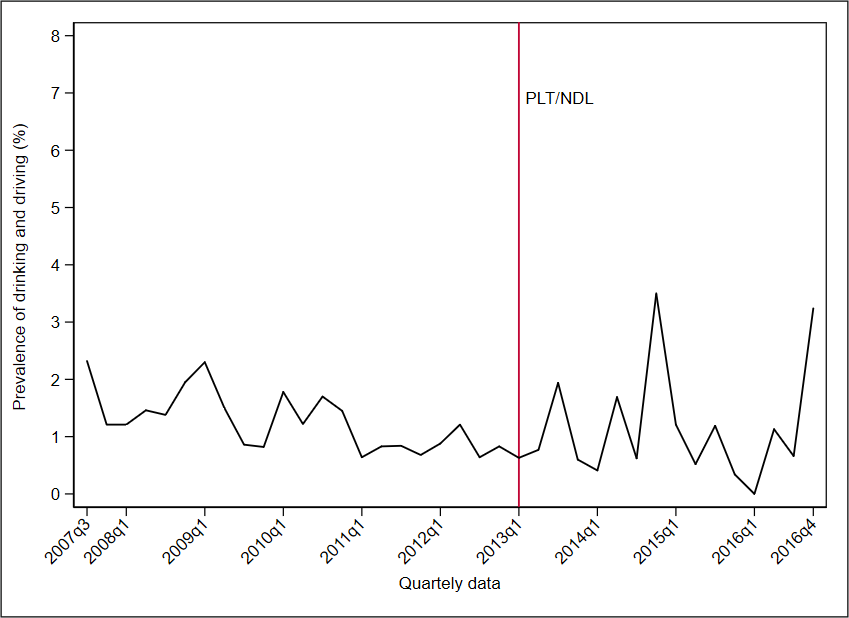


**Fig 21. Prevalence of drinking and driving in the city of Salvador (state of Bahia), 2007q3 to 2016q4.** Abbreviations: PLT: Program Life in Traffic; NDL: New Dry Law


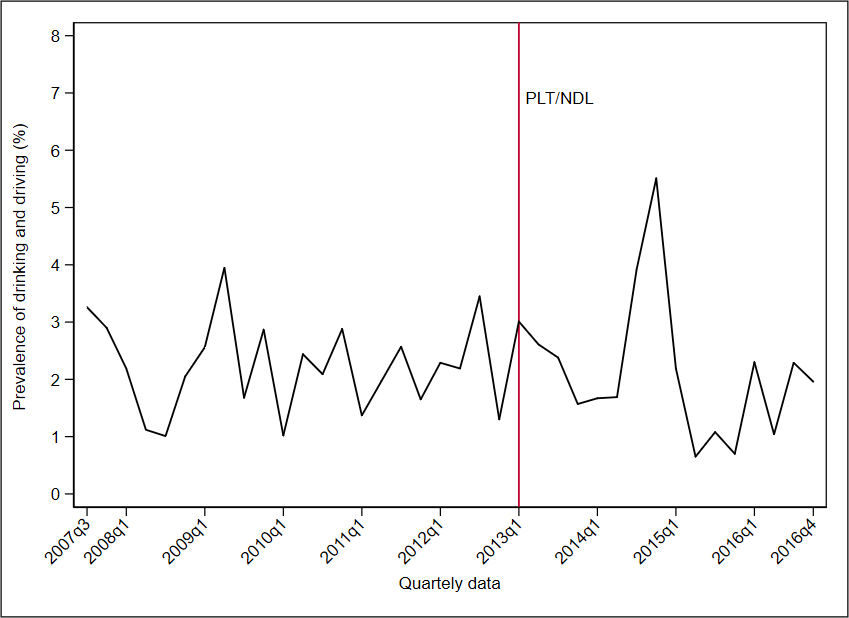


**Fig 21. Prevalence of drinking and driving in the city of São Luís (state of Maranhão), 2007q3 to 2016q4.** Abbreviations: PLT: Program Life in Traffic; NDL: New Dry Law
