# Supplementary material for: Impact of the program life in traffic and new zero-tolerance drinking and driving law on the prevalence of driving after alcohol abuse in Brazilian capitals: An interrupted time series analysis
Source: PLoS One. 2023 Oct 20;18(10):e0288288. doi: 10.1371/journal.pone.0288288 (PMC10588900; doi:10.1371/journal.pone.0288288)
Supplement: S2 File — (DOCX) [file pone.0288288.s005.docx]

**File S2.** Decomposition of temporal series

Figs 1 to 27 show temporal series decomposition of each capital analyzed.

**Southeast macro-region**


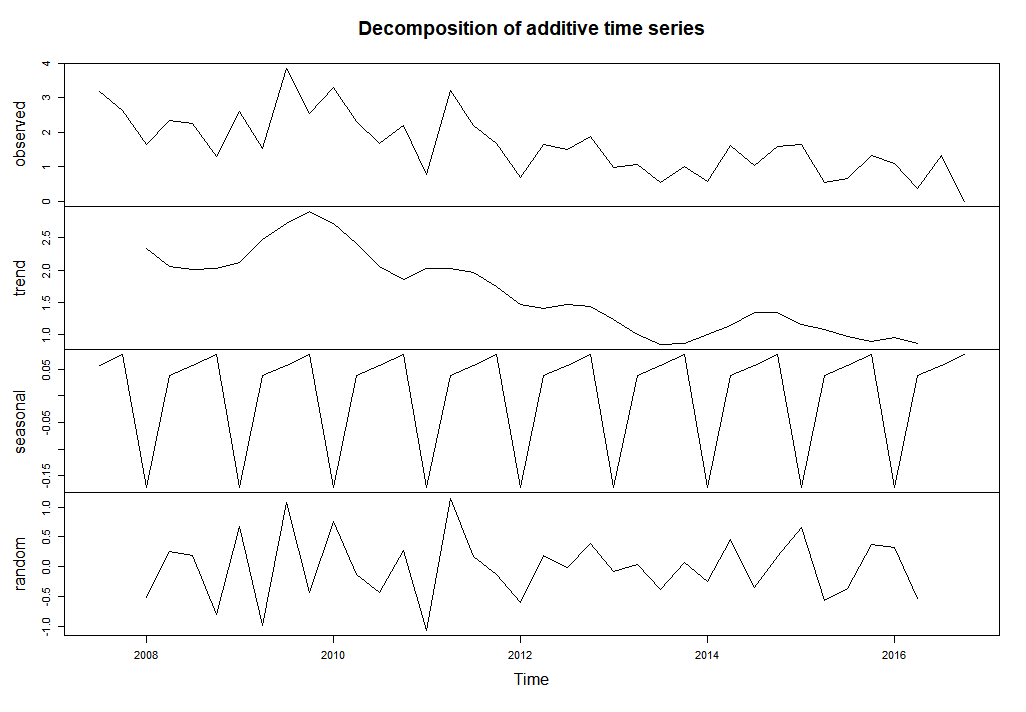


**Fig 1**. Decomposition of the temporal series in trend, seasonality, and irregularity of the city of Belo Horizonte (state of Minas Gerais).


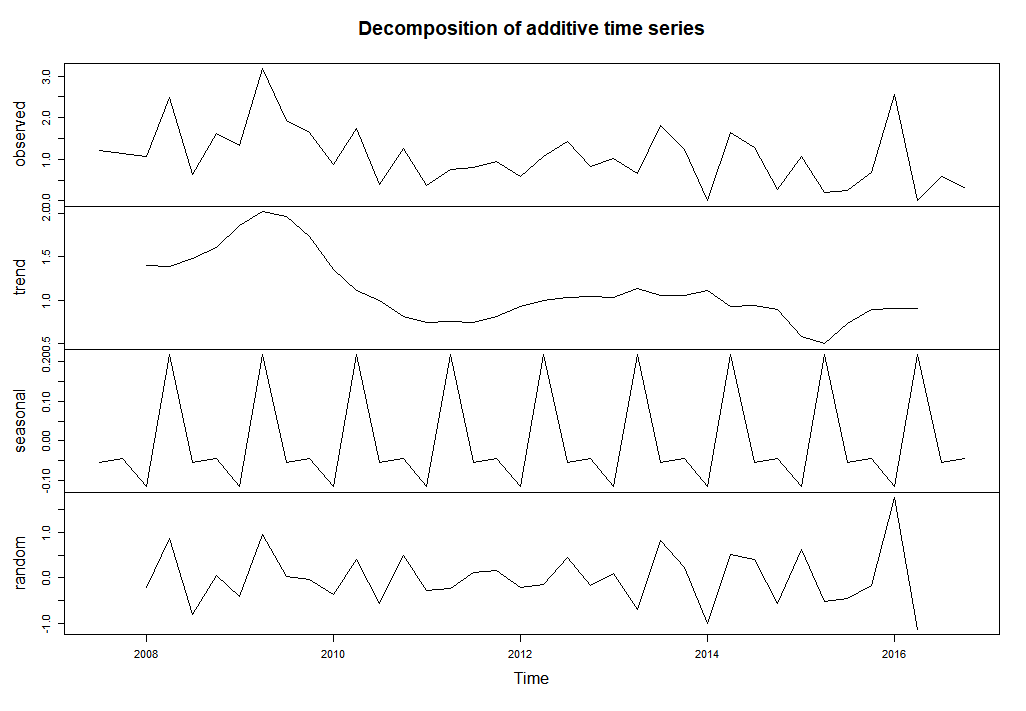


**Fig 2**. Decomposition of the temporal series in trend, seasonality, and irregularity of the city of Rio de Janeiro (state of Rio de Janeiro)


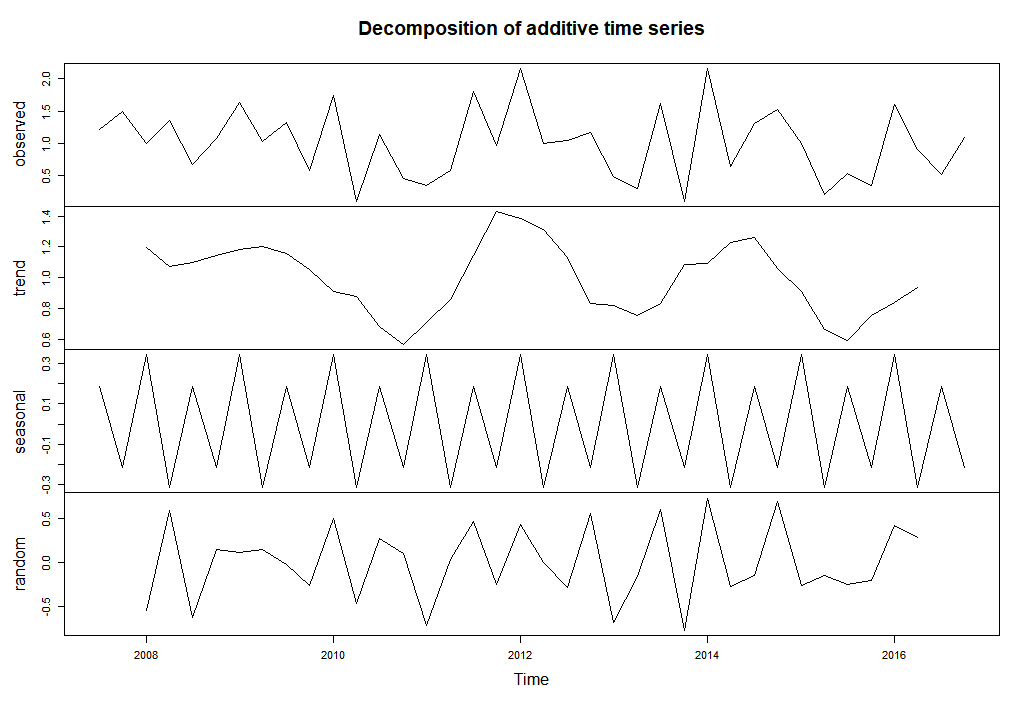


**Figura 3**. Decomposition of the temporal series in trend, seasonality, and irregularity of the city of São Paulo (state of São Paulo)


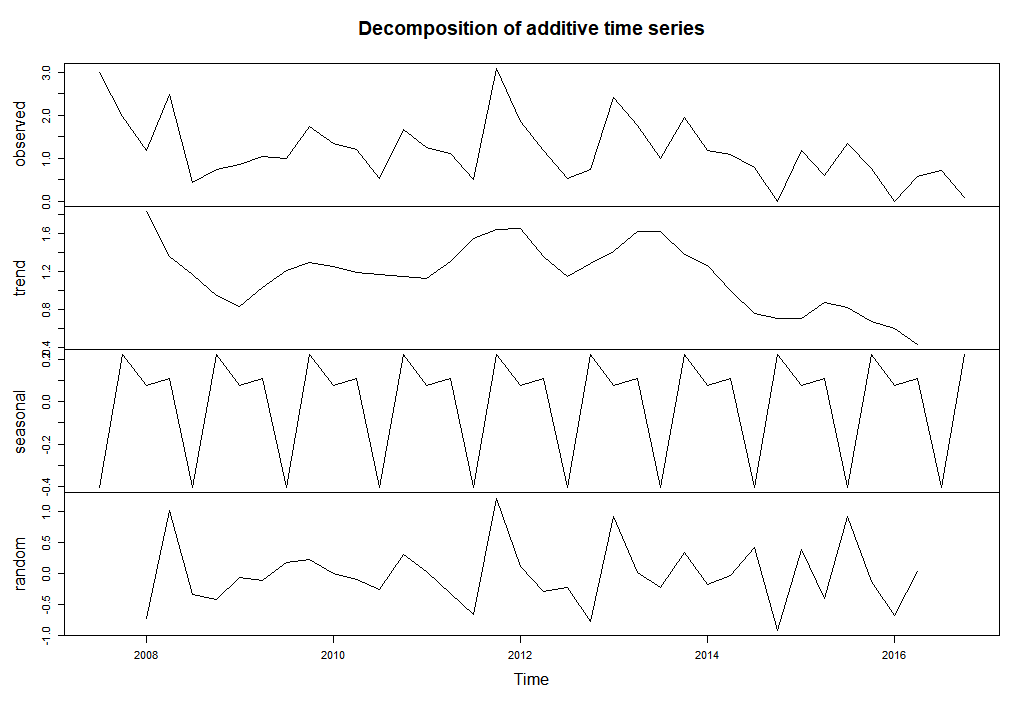


**Fig 4**. Decomposition of the temporal series in trend, seasonality, and irregularity of the city of Vitória (State of Espírito Santo)

**South macro-region**

**
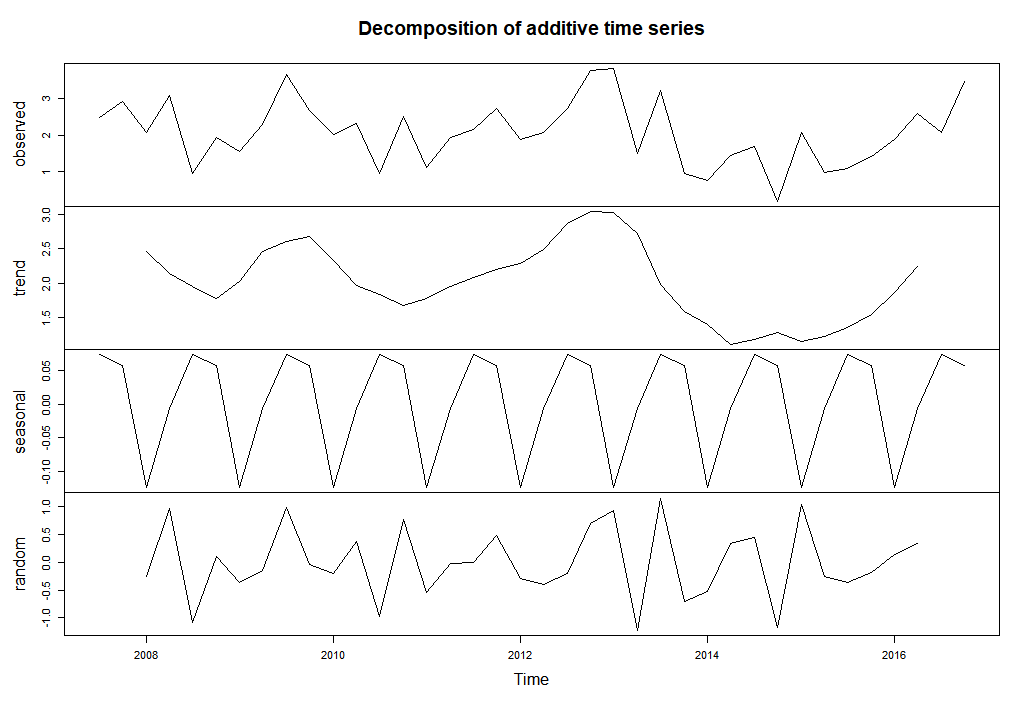
**

**Fig 5**. Decomposition of the temporal series in trend, seasonality, and irregularity of the city of Curitiba (State of Paraná)

**
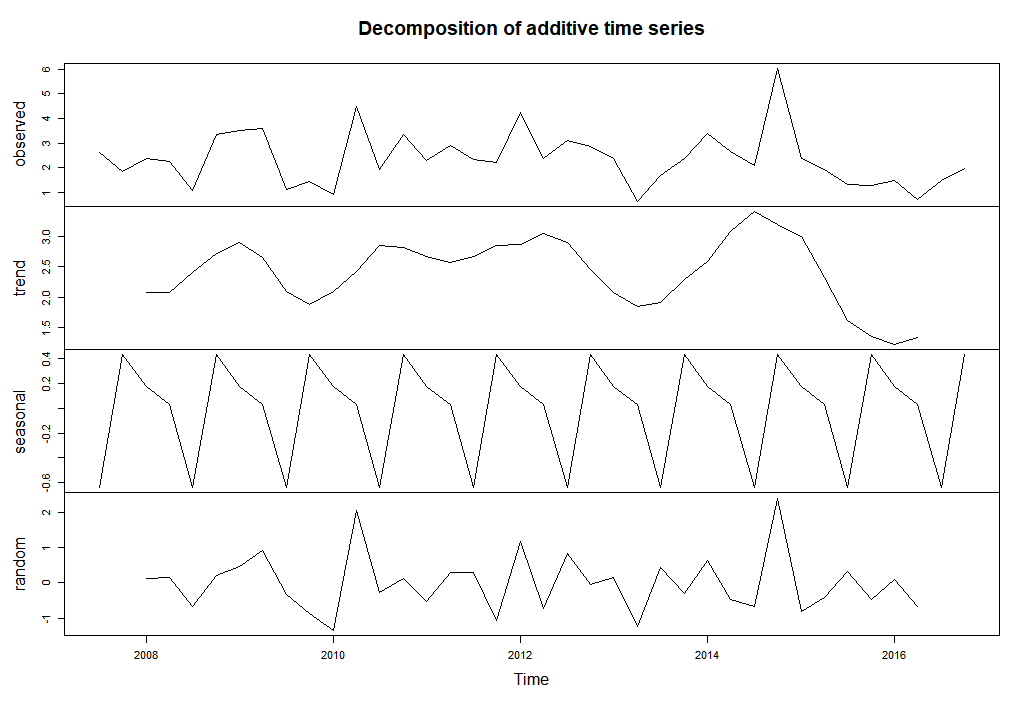
**

**Fig 6**. Decomposition of the temporal series in trend, seasonality, and irregularity of the city of Florianópolis (state of Santa Catarina)


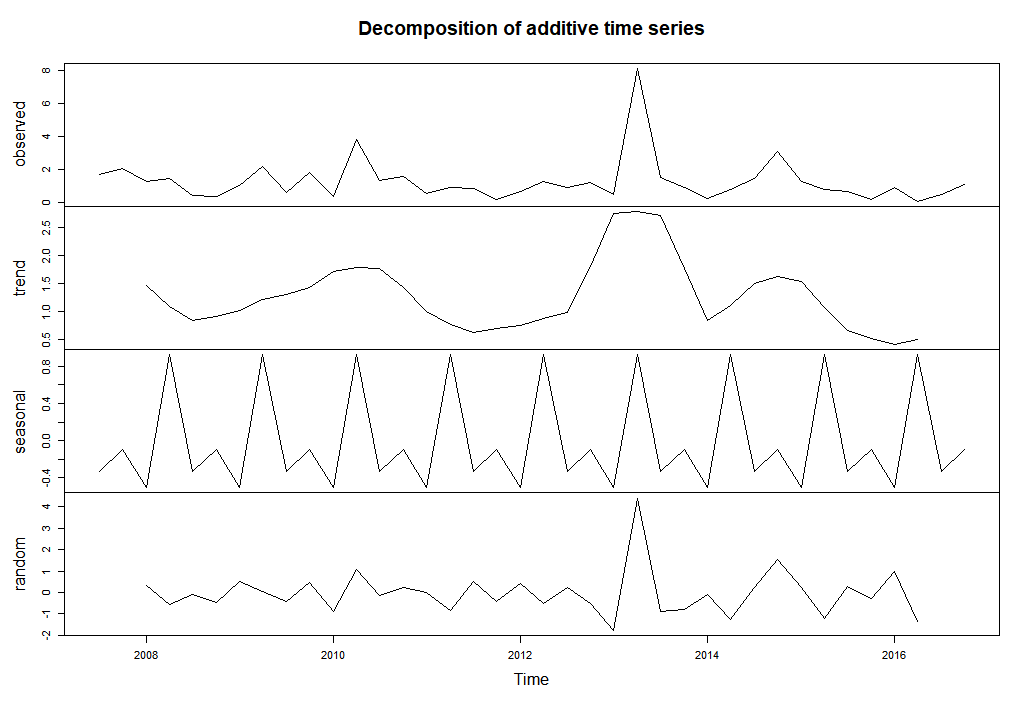


**Fig 7** Decomposition of the temporal series in trend, seasonality, and irregularity of the city of Porto Alegre (state of Rio Grande do Sul).

**Midwest macro-region**


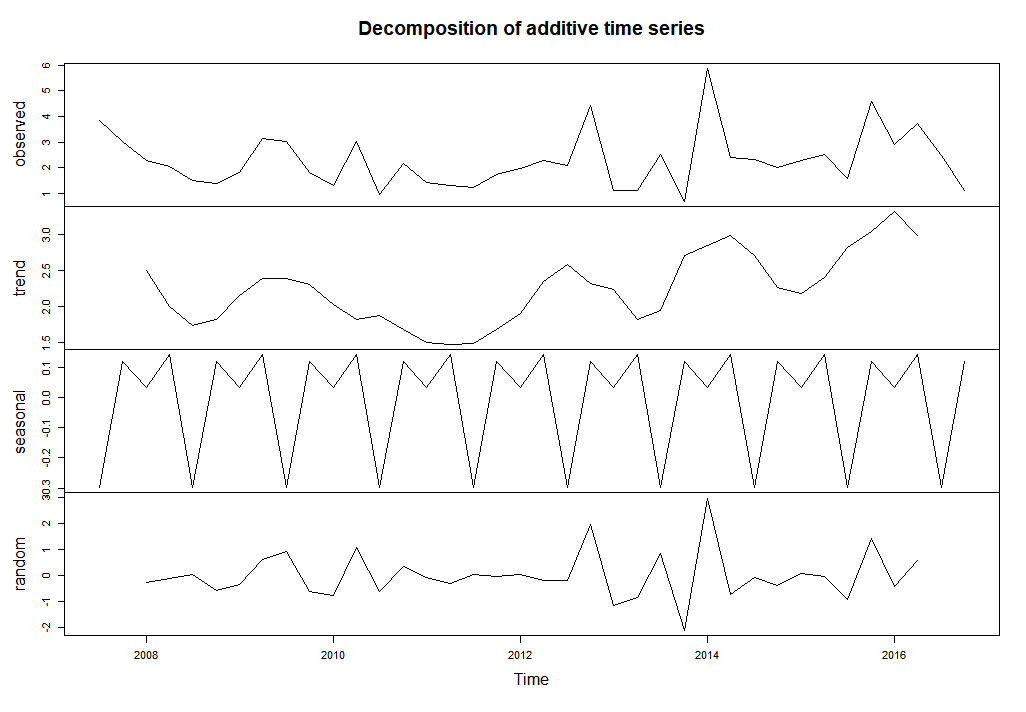


**Fig 8**. Decomposition of the temporal series in trend, seasonality, and irregularity of the city of Brasília (Distrito Federal).


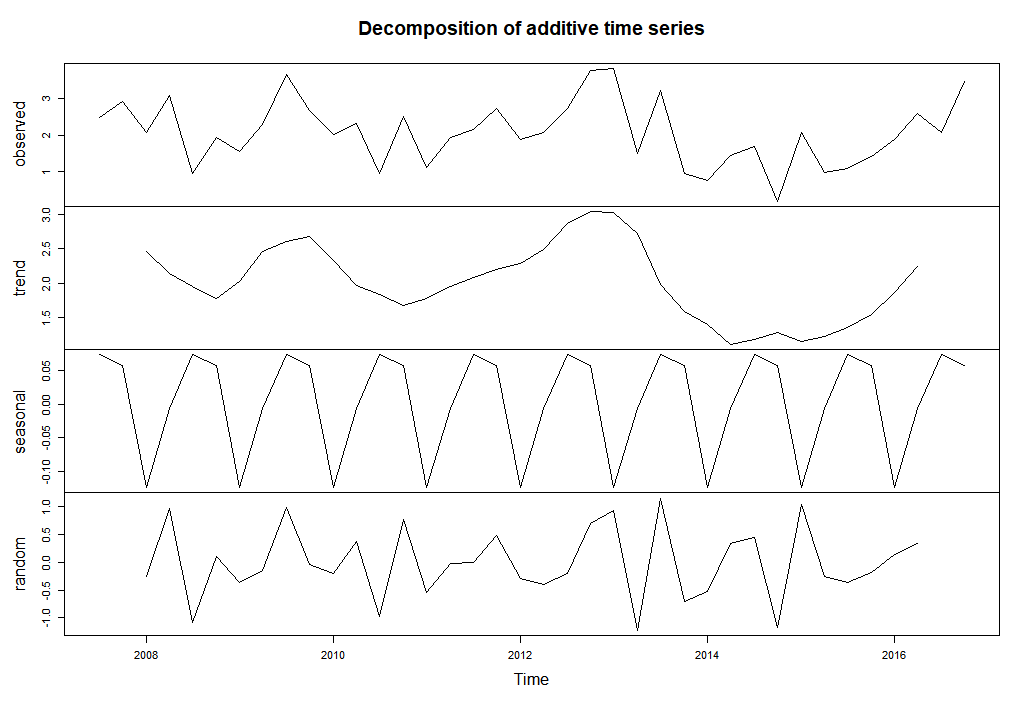


**Fig 9**. Decomposition of the temporal series in trend, seasonality, and irregularity of the city of Campo Grande (state of Mato Grosso do Sul)


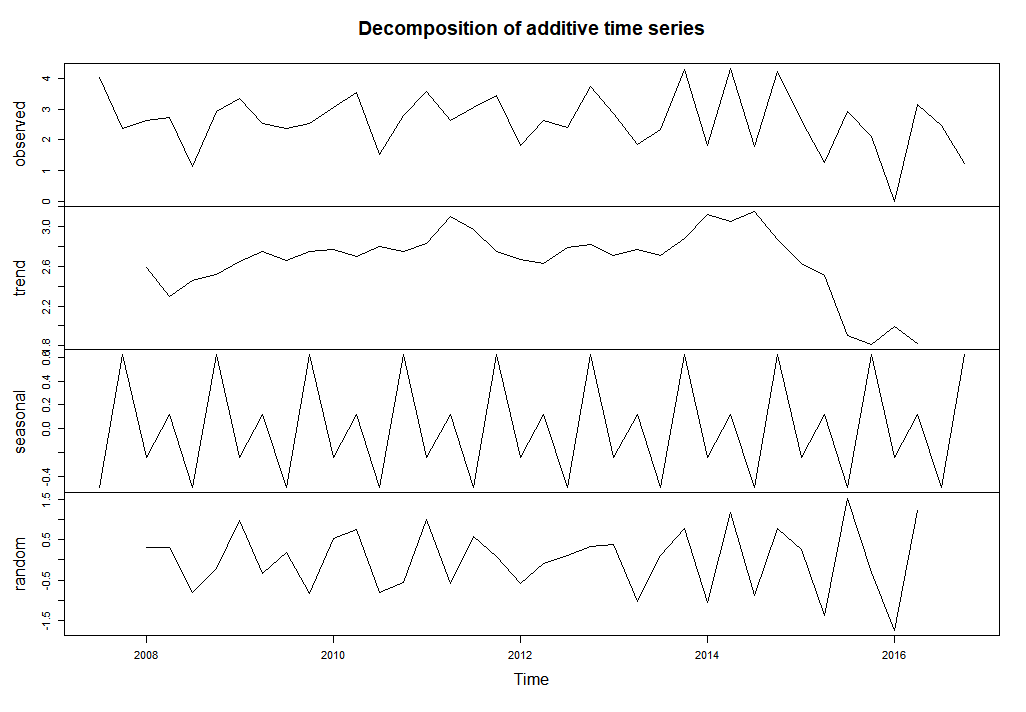


**Fig 10**. Decomposition of the temporal series in trend, seasonality, and irregularity of the city of Cuiabá (state of Mato Grosso State)


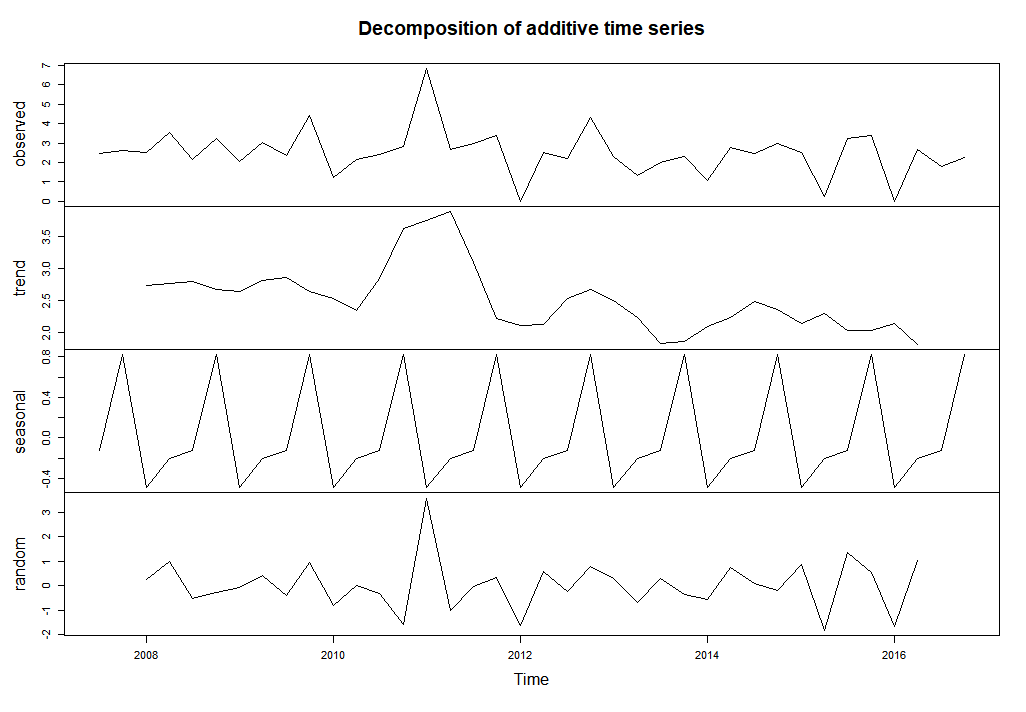


**Fig 11**. Decomposition of the temporal series in trend, seasonality, and irregularity of the city of Goiânia (state of Goiás).

**North macro-region**


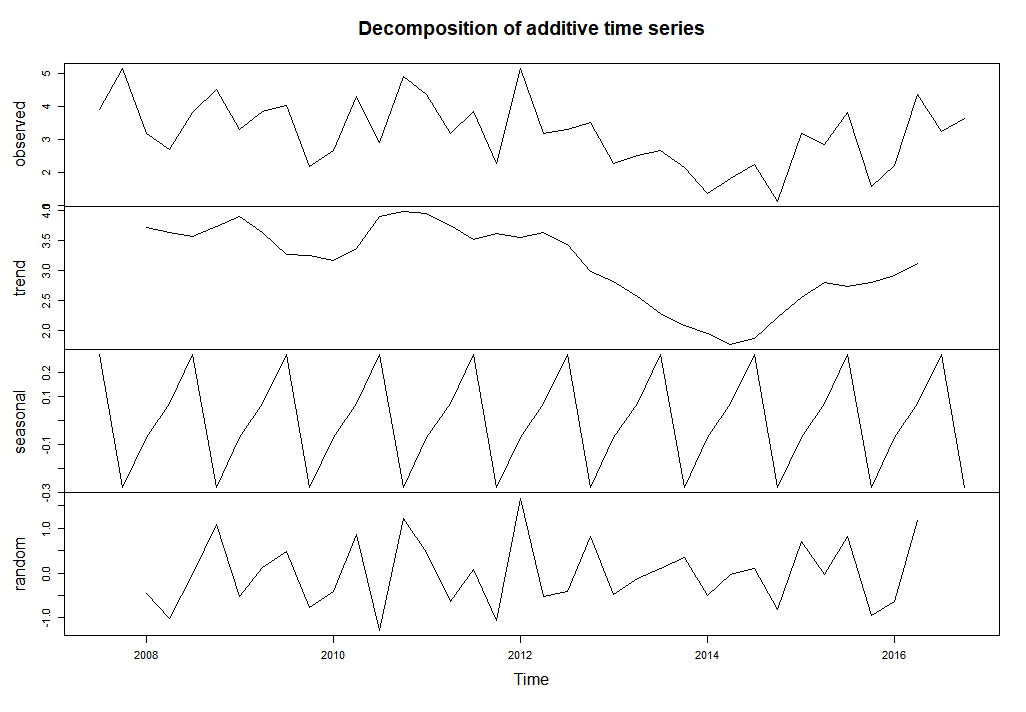


**Fig 12**. Decomposition of the temporal series in trend, seasonality, and irregularity of the city of Palmas (state of Tocantins)


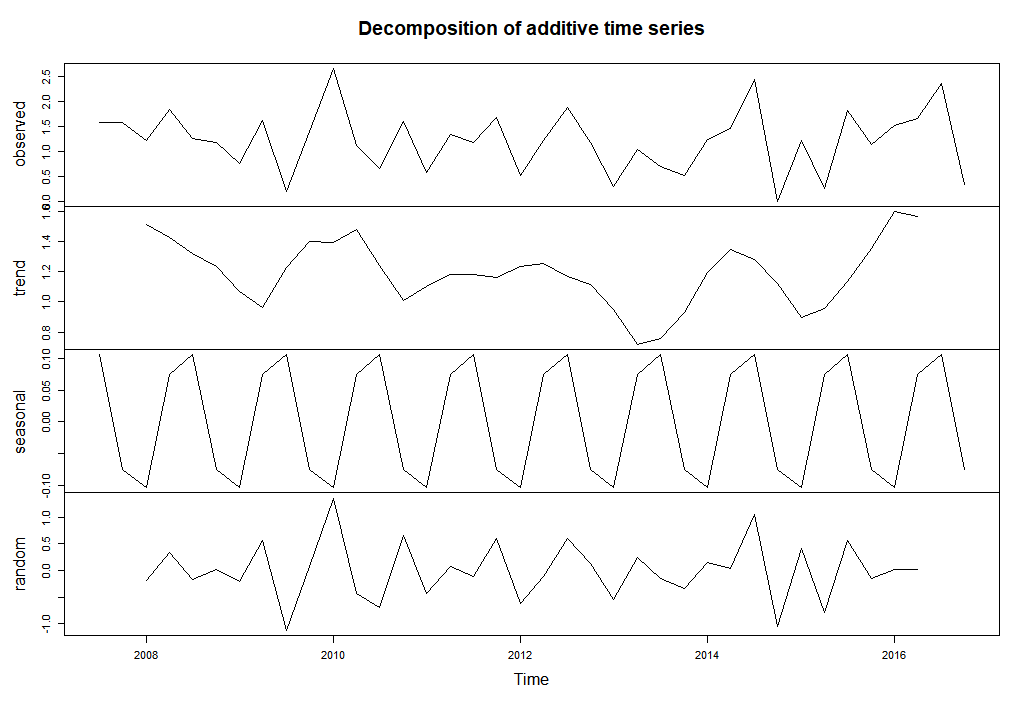


**Fig 13**. Decomposition of the temporal series in trend, seasonality, and irregularity of the city of Belém (state of Pará)


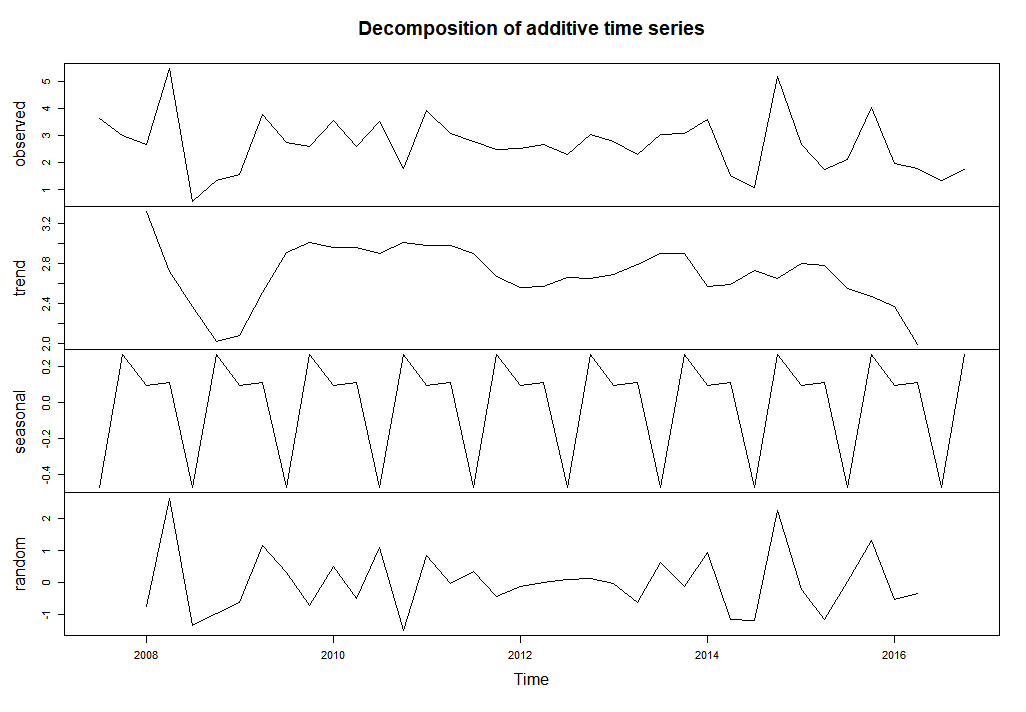


**Fig 14**. Decomposition of the temporal series in trend, seasonality, and irregularity of the city of Boa Vista (state of Roraima).


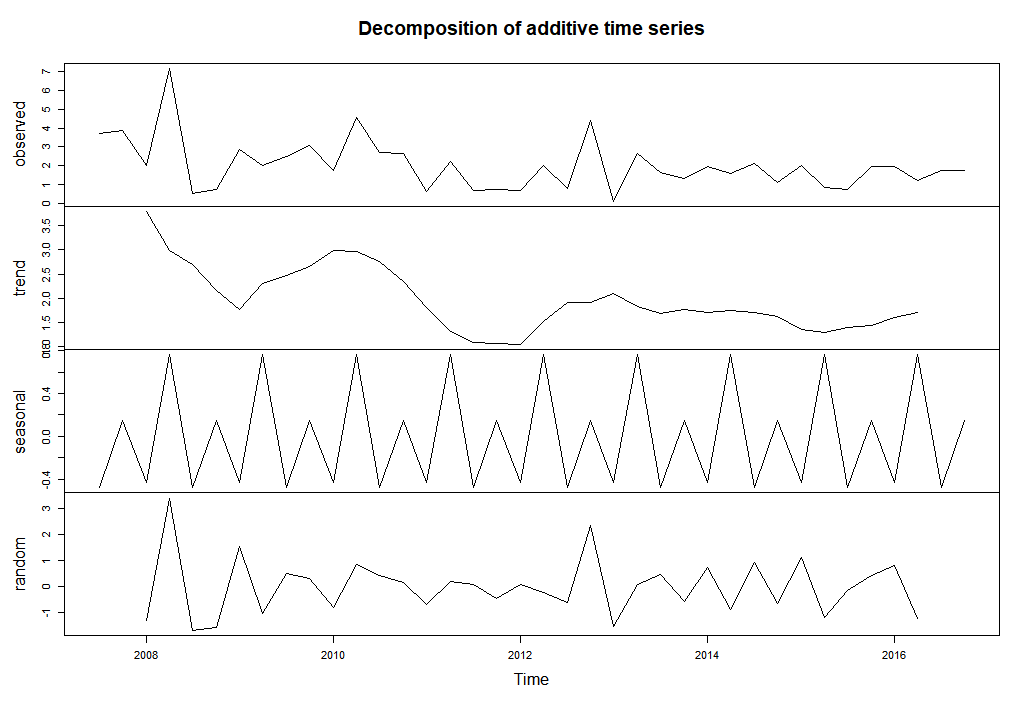


**Fig 15**. Decomposition of the temporal series in trend, seasonality, and irregularity of the city of Macapá (state of Amapá).


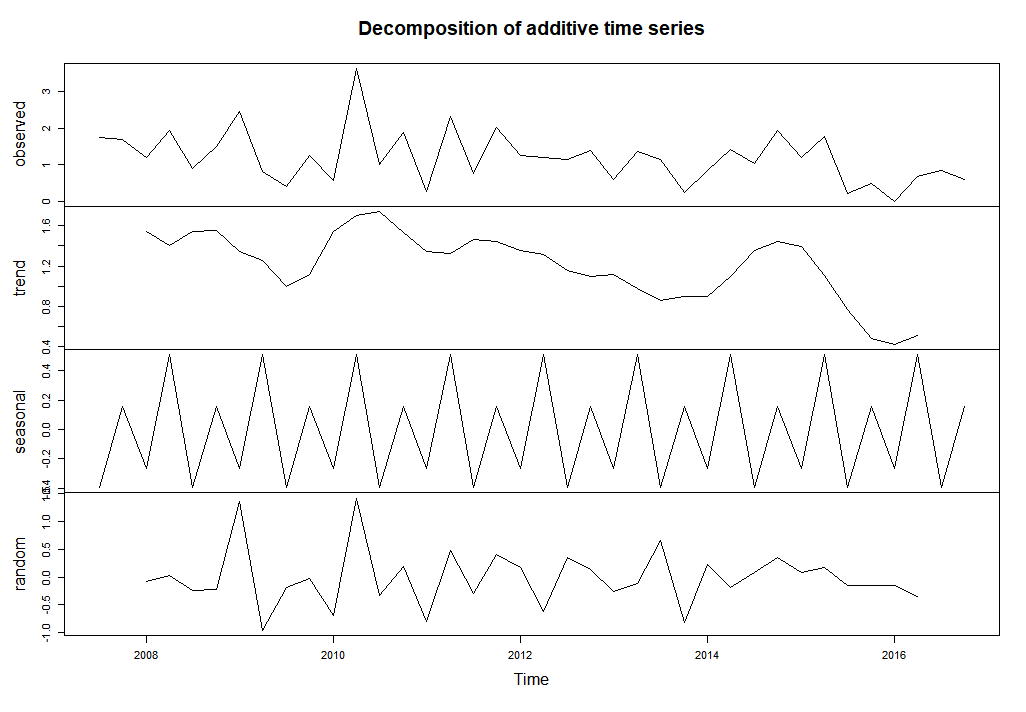


**Fig 16**. Decomposition of the temporal series in trend, seasonality, and irregularity of the city of Manaus (state of Amazonas)


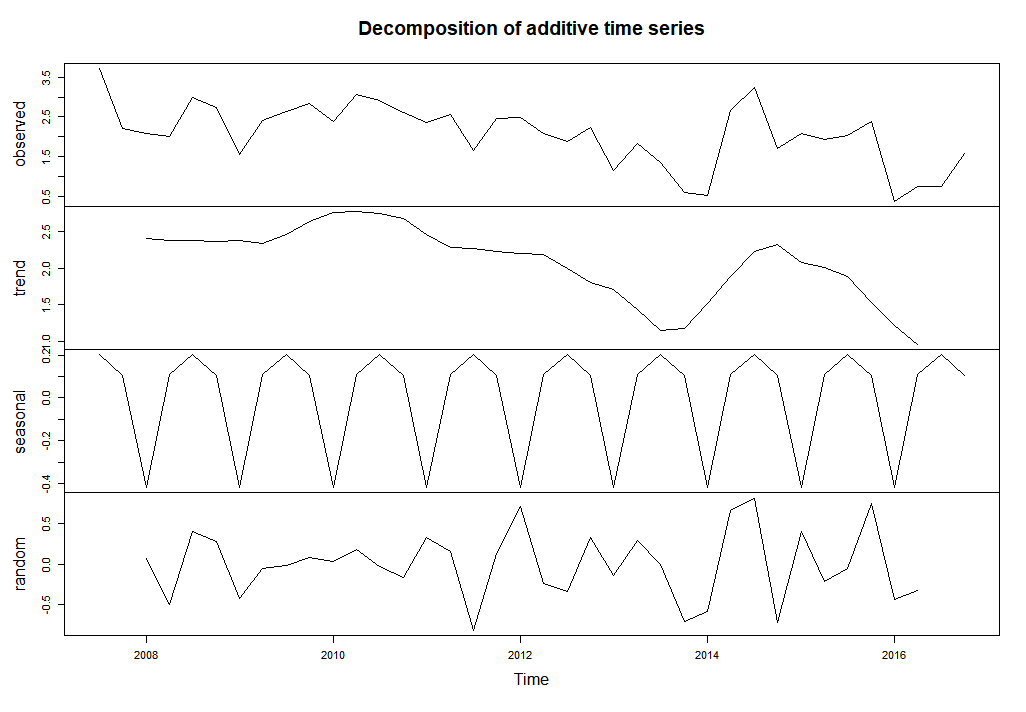


**Fig 17**. Decomposition of the temporal series in trend, seasonality, and irregularity of the city of Porto Velho (state of Rondônia)


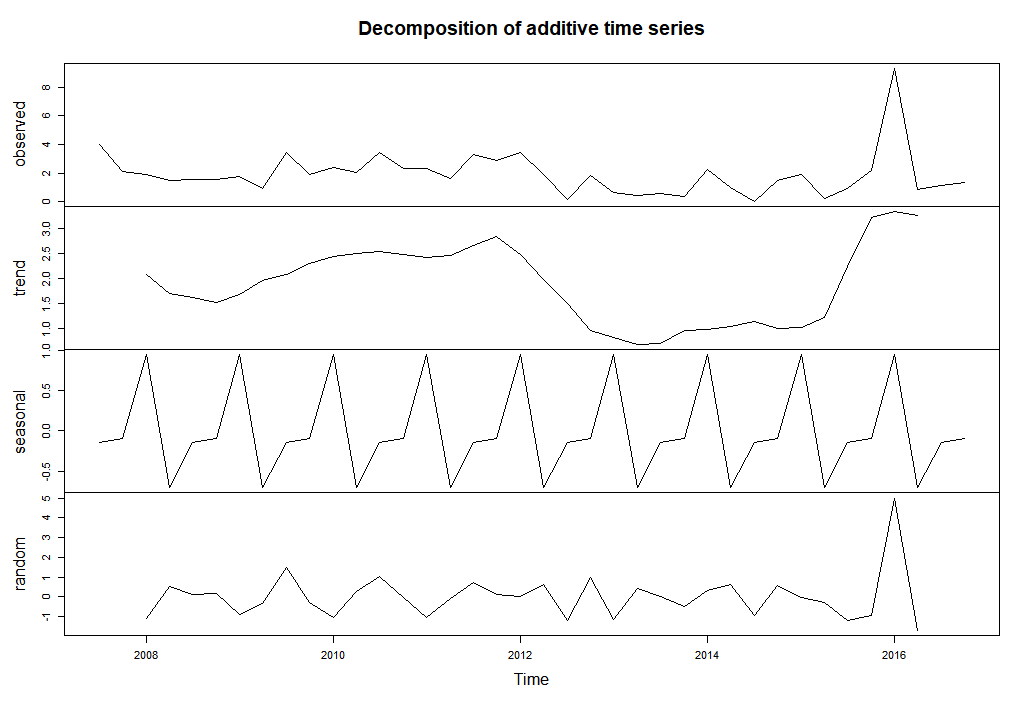


**Fig 18**. Decomposition of the temporal series in trend, seasonality, and irregularity of the city of Rio Branco (State of Acre).

**Northeast macro-region**


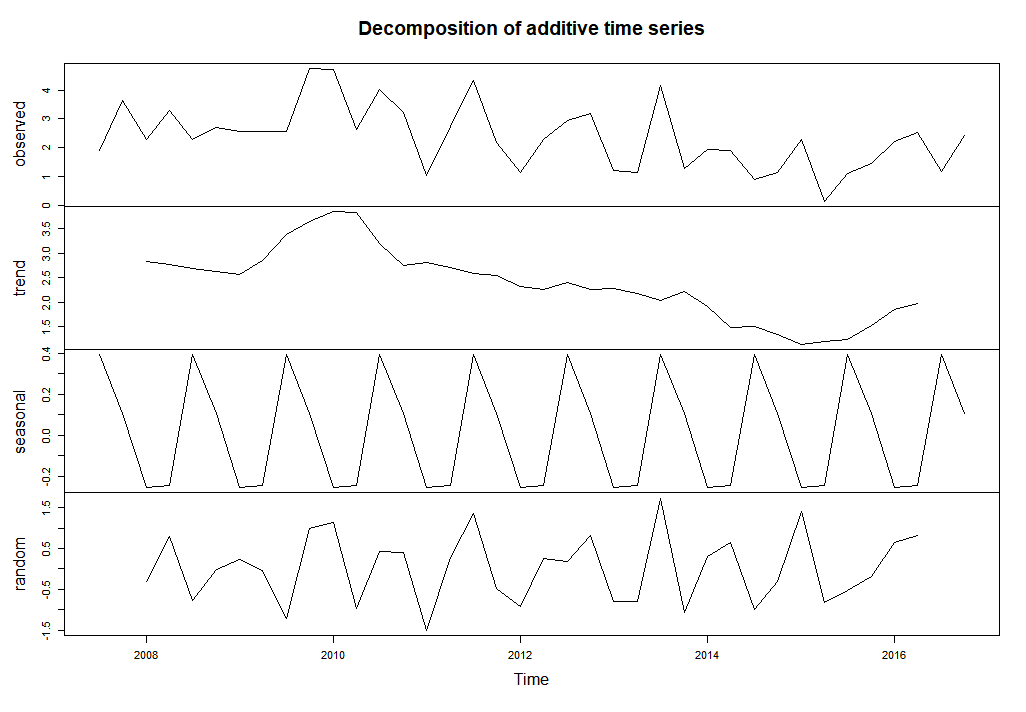


**Fig 19**. Decomposition of the temporal series in trend, seasonality, and irregularity of the city of Aracaju (state of Sergipe)


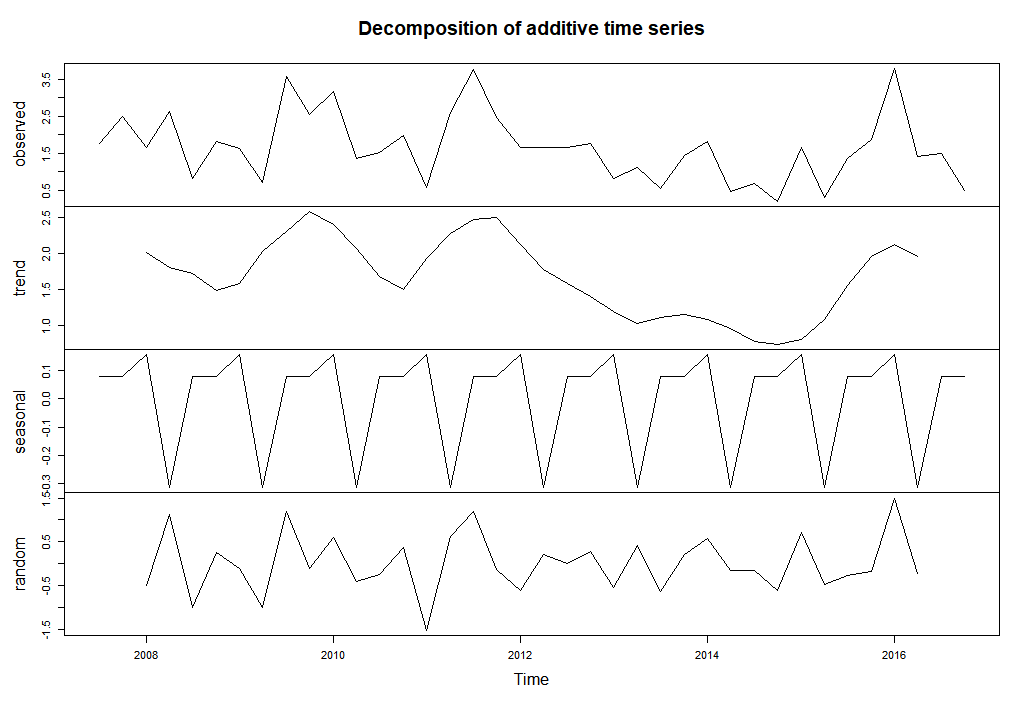


**Fig 20**. Decomposition of the temporal series in trend, seasonality, and irregularity of the city of Fortaleza (state of Ceará)


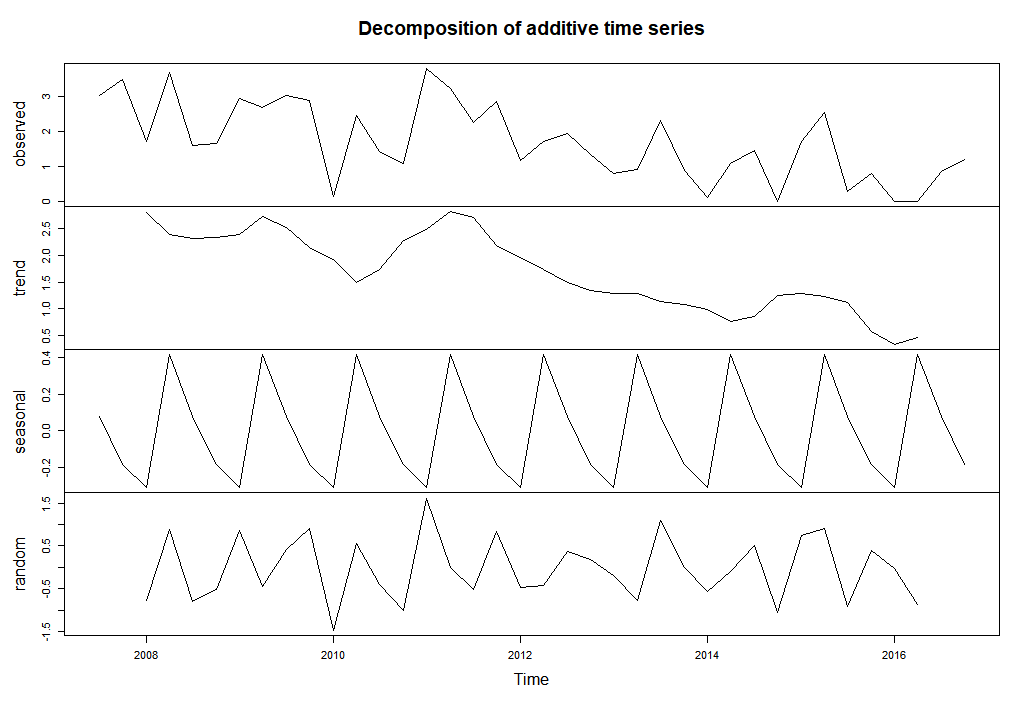


**Fig 21**. Decomposition of the temporal series in trend, seasonality, and irregularity of the city of João Pessoa (state of Paraíba)


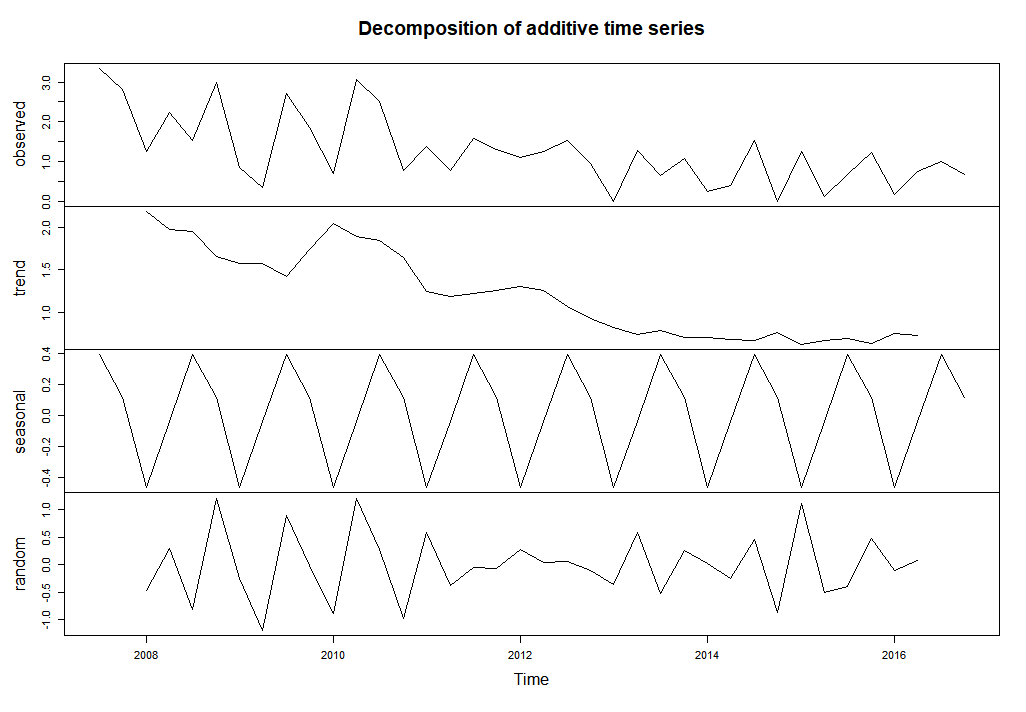


**Fig 22**. Decomposition of the temporal series in trend, seasonality, and irregularity of the city of Maceió (state of Alagoas)


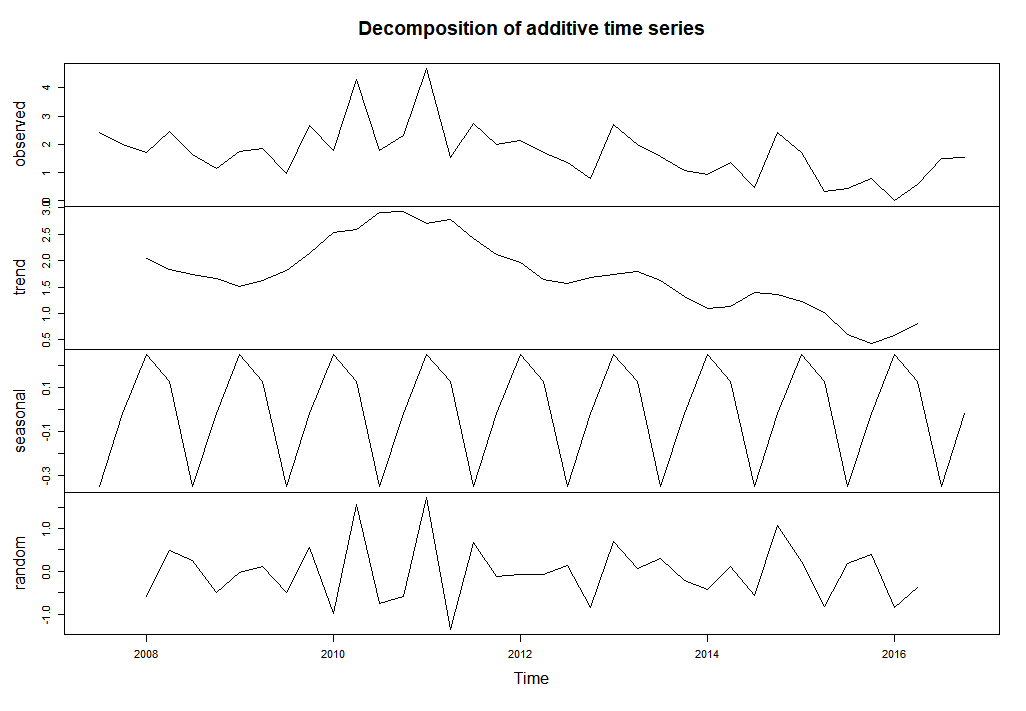


**Fig 23**. Decomposition of the temporal series in trend, seasonality, and irregularity of the city of Natal (state of Rio Grande do Norte)


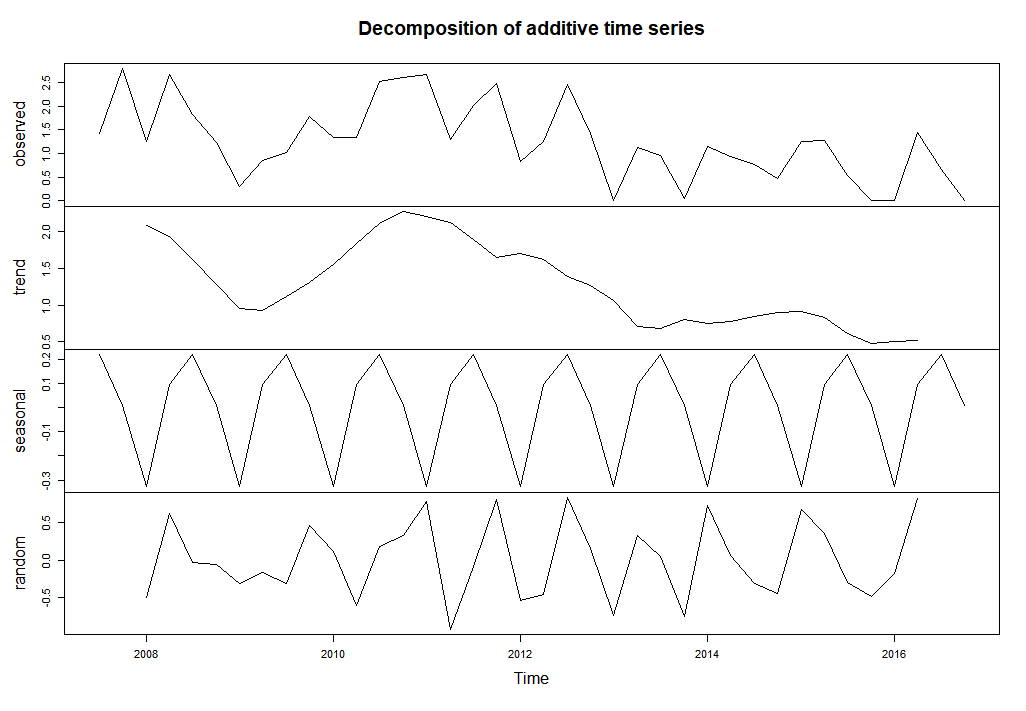


**Fig 24**. Decomposition of the temporal series in trend, seasonality, and irregularity of the city of Recife (state of Pernambuco)


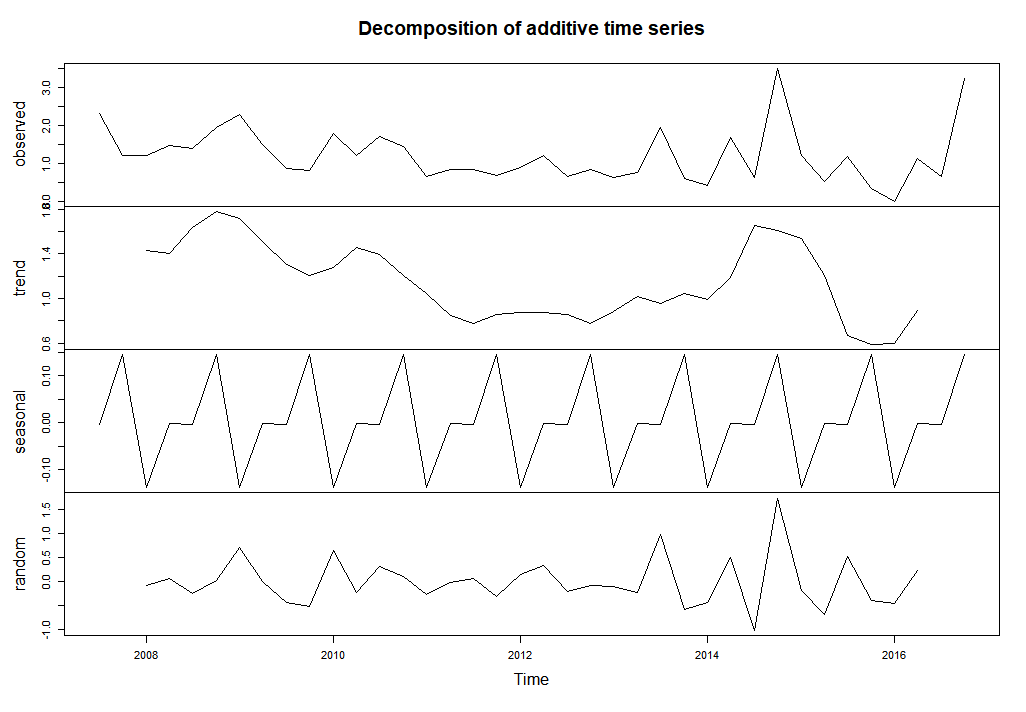


**Fig 25** Decomposition of the temporal series in trend, seasonality, and irregularity of the city of Salvador (state of Bahia)


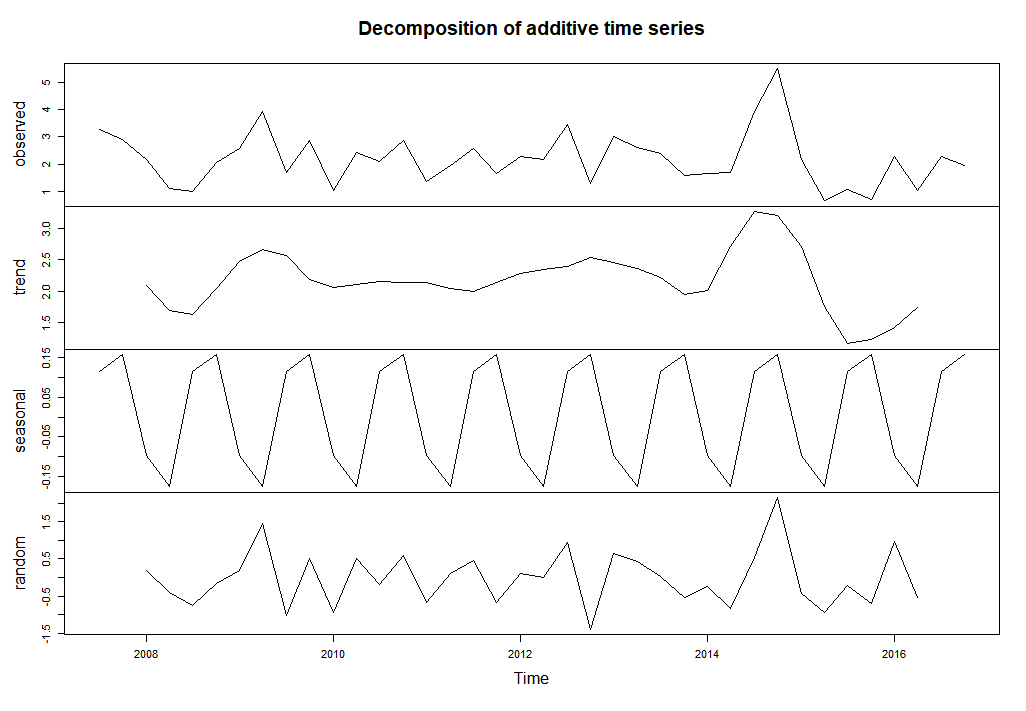


**Fig 26**. Decomposition of the temporal series in trend, seasonality, and irregularity of the city of São Luís (state of Maranhão)


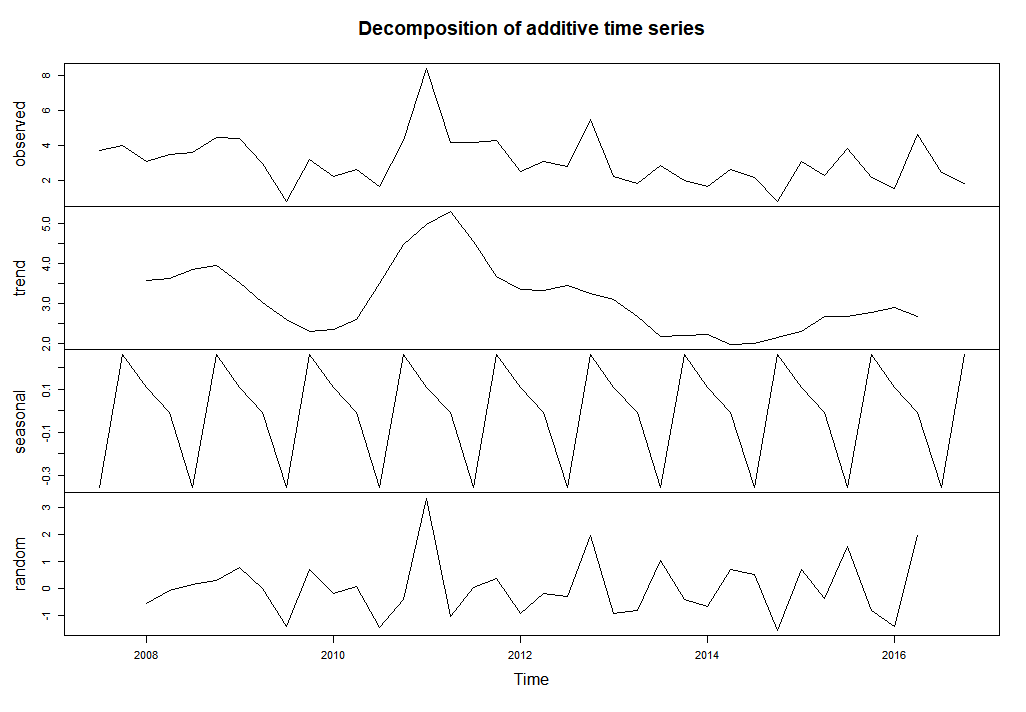


**Fig 27**. Decomposition of the temporal series in trend, seasonality, and irregularity of the city of Teresina (state of Piauí);
