# Supplementary material for: Impact of the program life in traffic and new zero-tolerance drinking and driving law on the prevalence of driving after alcohol abuse in Brazilian capitals: An interrupted time series analysis
Source: PLoS One. 2023 Oct 20;18(10):e0288288. doi: 10.1371/journal.pone.0288288 (PMC10588900; doi:10.1371/journal.pone.0288288)
Supplement: S3 File — (DOCX) [file pone.0288288.s006.docx]

**File S3.** Temporal series ACF and PACF

Figs 1 to 27 show the ACF and PACF of the temporal series of each capital analyzed. The graphs were used in the selection of the temporal series models orders.

**Southeast macro-region**


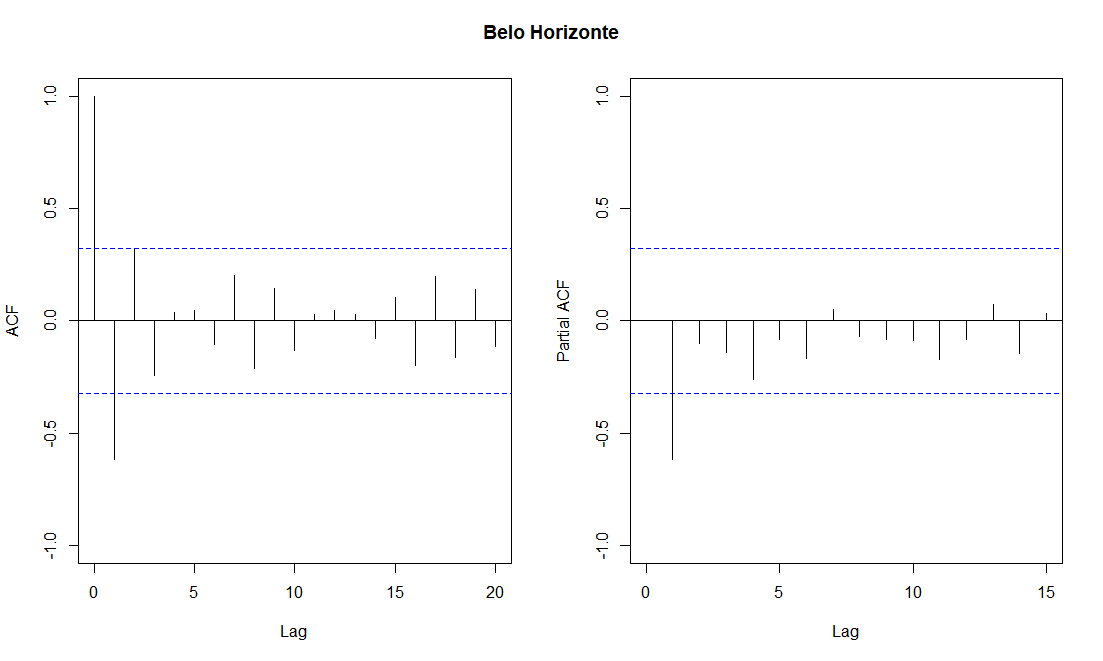


**Fig 1**. ACF and PACF for the city of Belo Horizonte (state of Minas Gerais) [following differentiation of order 1]


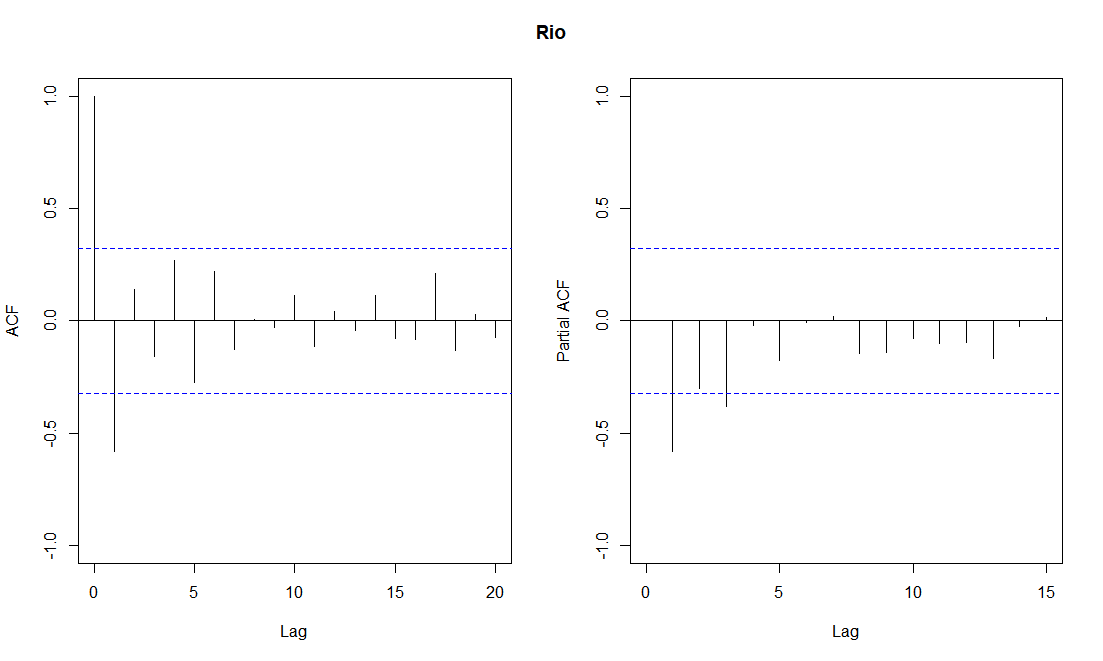


**Fig 2**. ACF and PACF for the city of Rio de Janeiro (state of Rio de Janeiro) [following order differentiation of order 1]


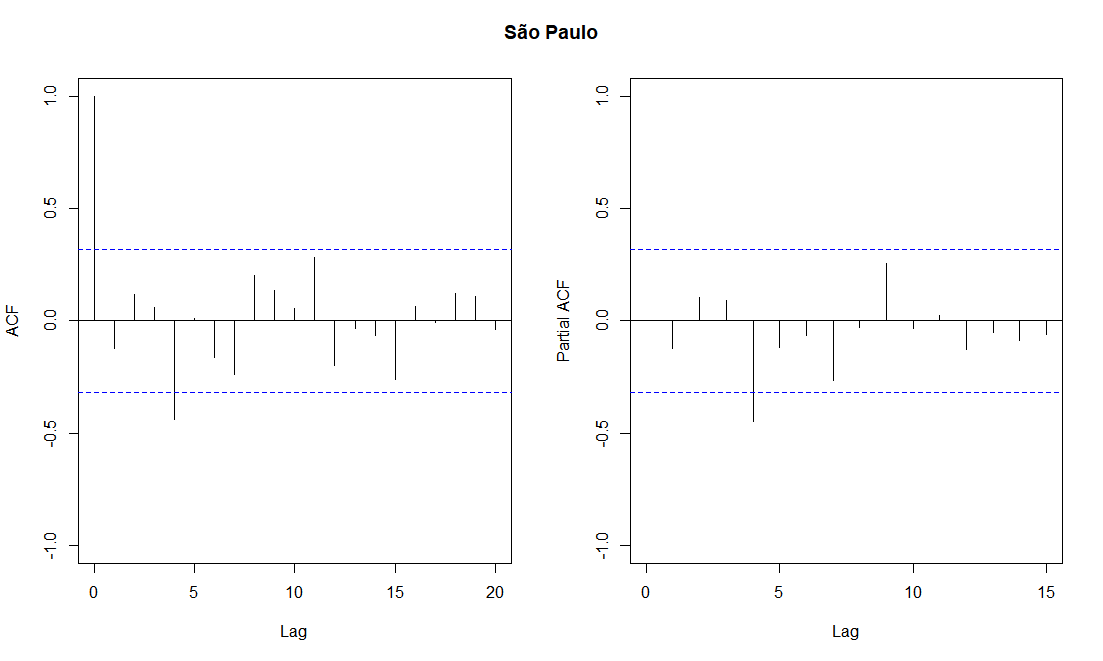


**Fig 3**. ACF and PACF for the city of São Paulo (state of São Paulo) [following seasonal adjustment and differentiation of order 1]


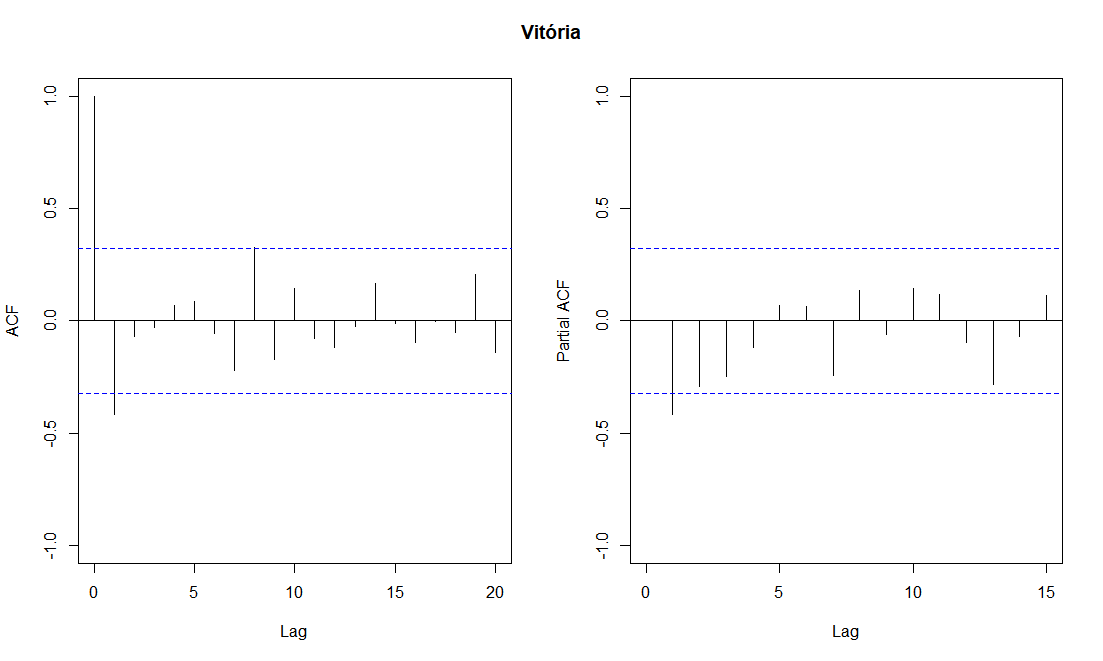


**Fig 4**. ACF and PACF for the city of Vitória (state of Espírito Santo)

**South macro-region**

**
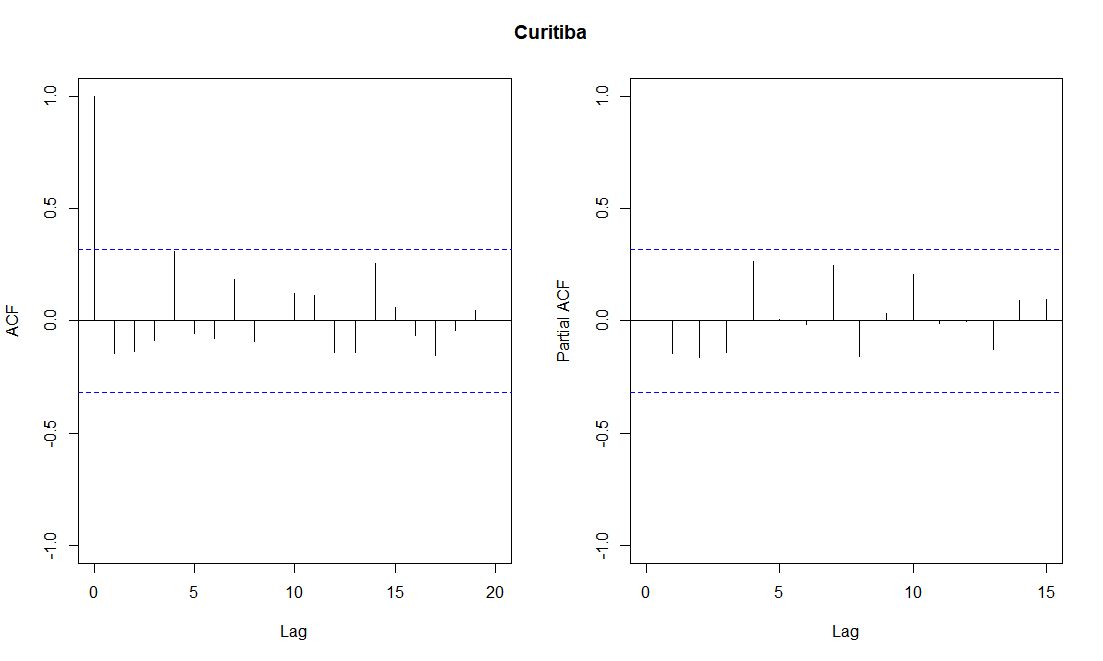
**

**Fig 5**. ACF and PACF for the city of Curitiba (state of Paraná)

**
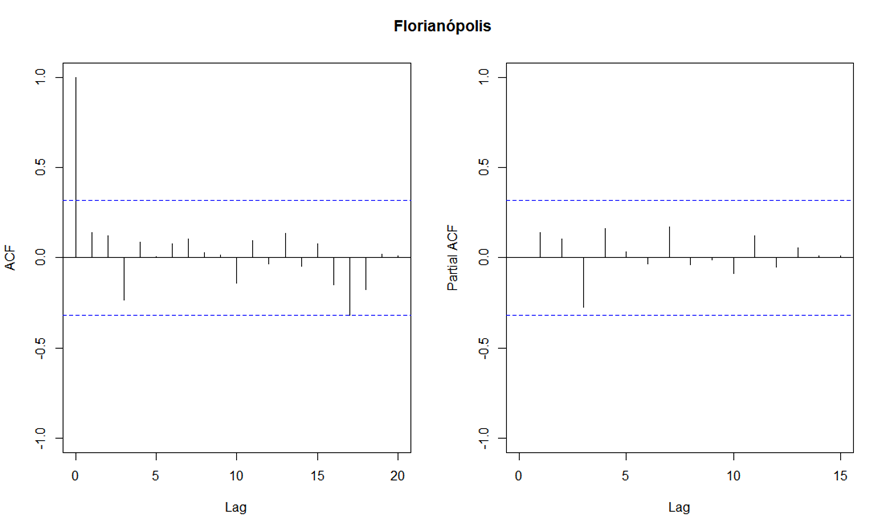
**

**Fig 6**. ACF and PACF for the city of Florianópolis (state of Santa Catarina) [following outlier replacement]


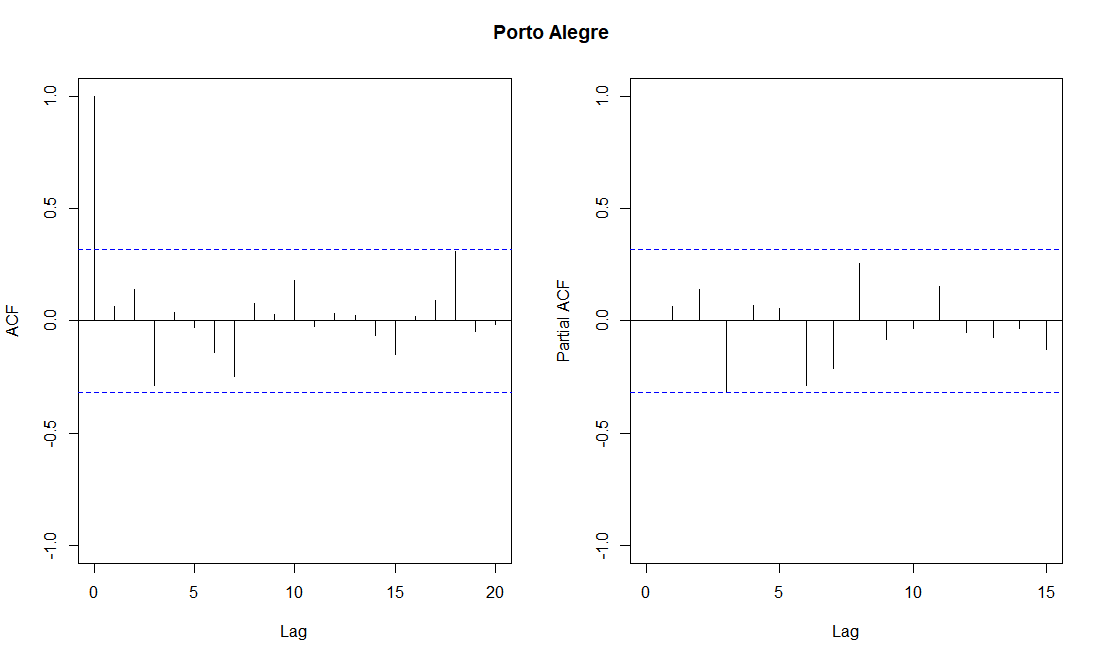


**Fig 7**. ACF and PACF for the city of Porto Alegre (state of Rio Grande do Sul). *ACF and PACF plotted after an outlier removal.

**Midwest macro-region**


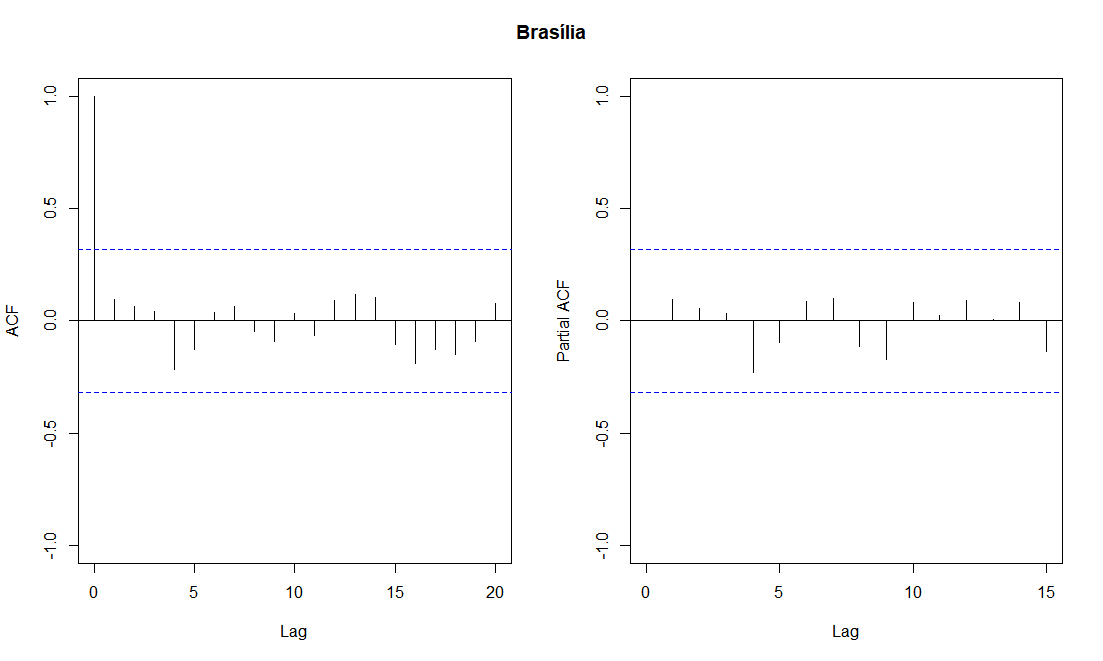


**Fig 8**. ACF and PACF for Brasília (Distrito Federal). *ACF and PACF plotted after an outlier removal.


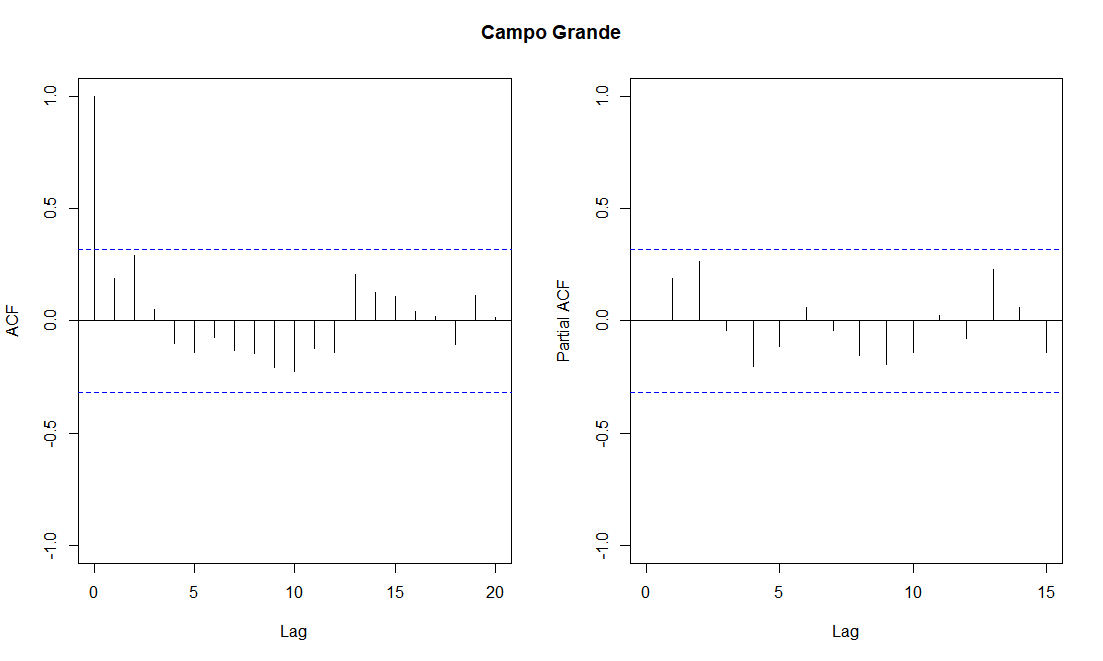


**Fig 9**. ACF and PACF for the city of Campo Grande (state of Mato Grosso do Sul)


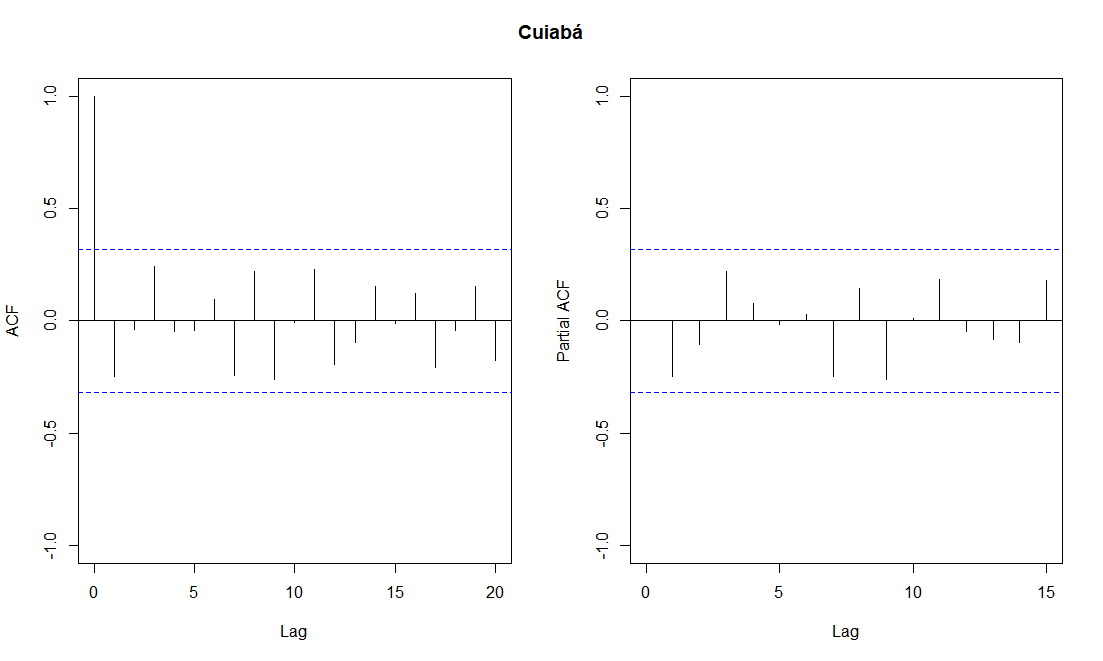


**Fig 10**. ACF and PACF for the city of Cuiabá (state of Mato Grosso)


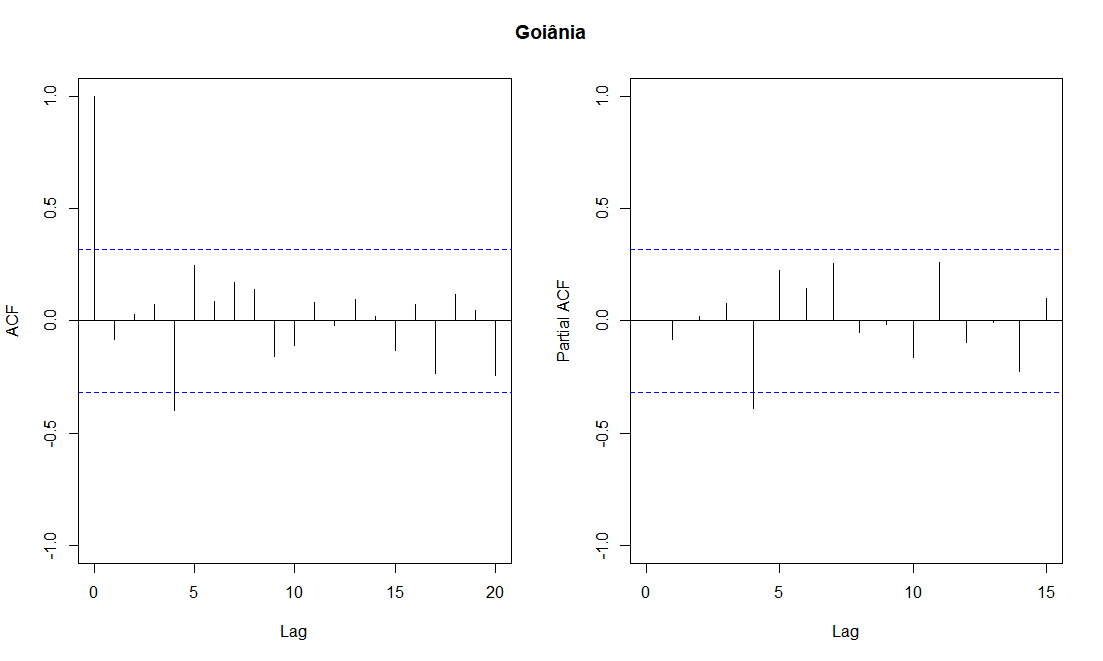


**Fig 11**. ACF and PACF for the city of Goiânia (state of Goiás). *ACF and PACF plotted after an outlier removal.

**North macro-region**


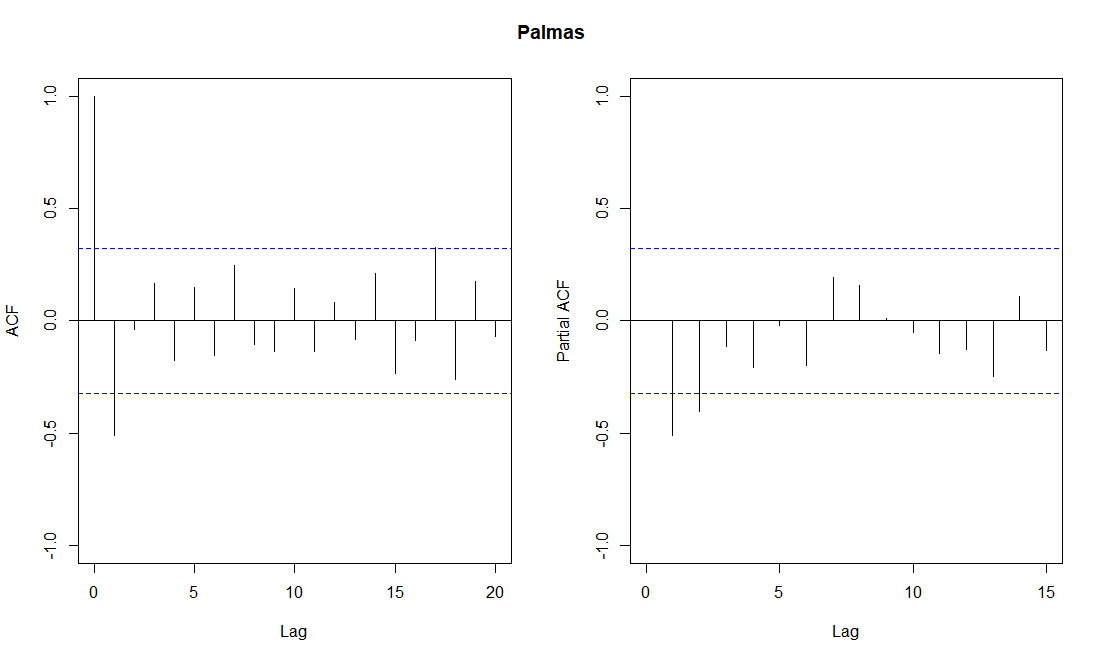


**Fig 12**. ACF and PACF for the city of Palmas (state of Tocantins)


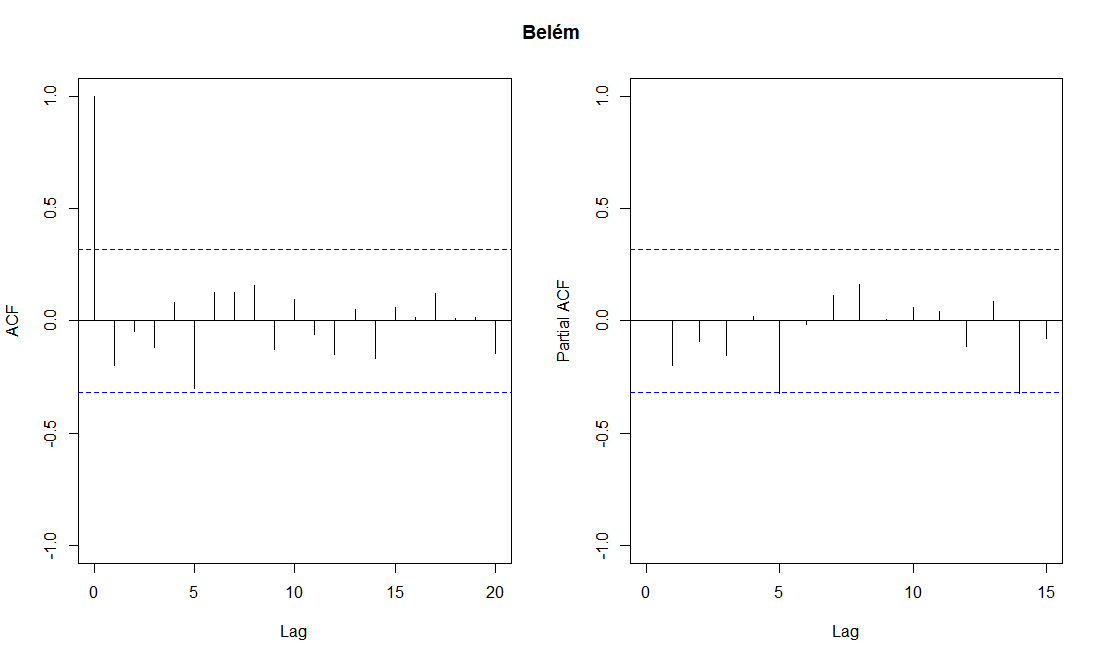


**Fig 13**. ACF and PACF for the city of Belém (State of Pará)


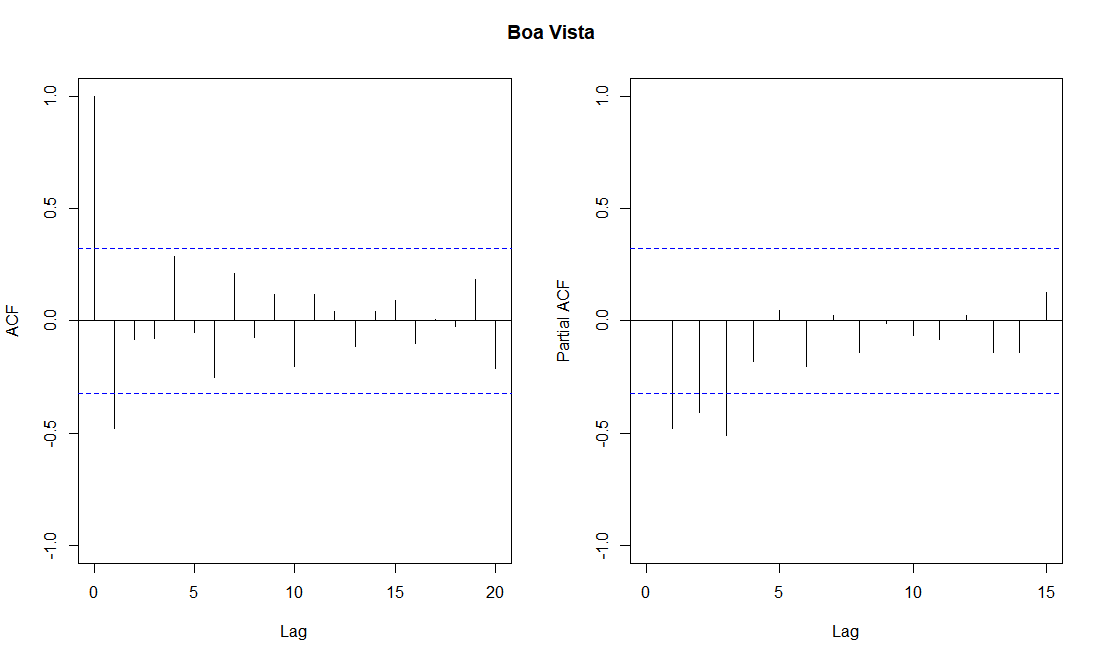


**Fig 14**. ACF and PACF for the city of Boa Vista (state of Roraima). *ACF and PACF plotted after an outlier removal.


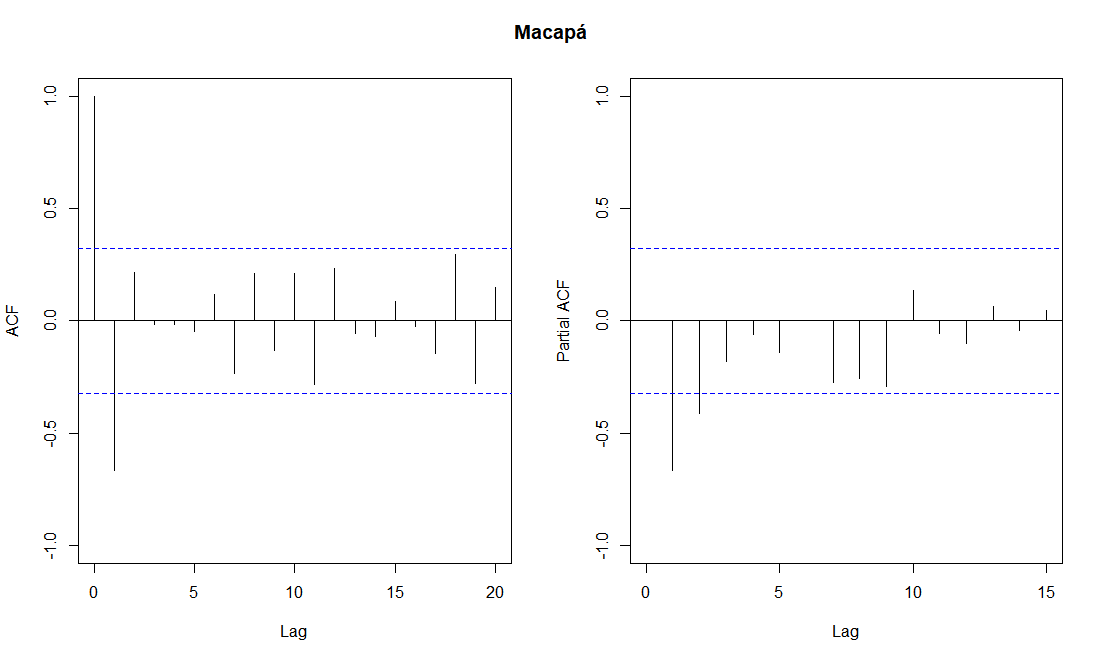


**Fig 15**. ACF and PACF for the city of Macapá (state of Amapá). *ACF and PACF plotted after an outlier removal.


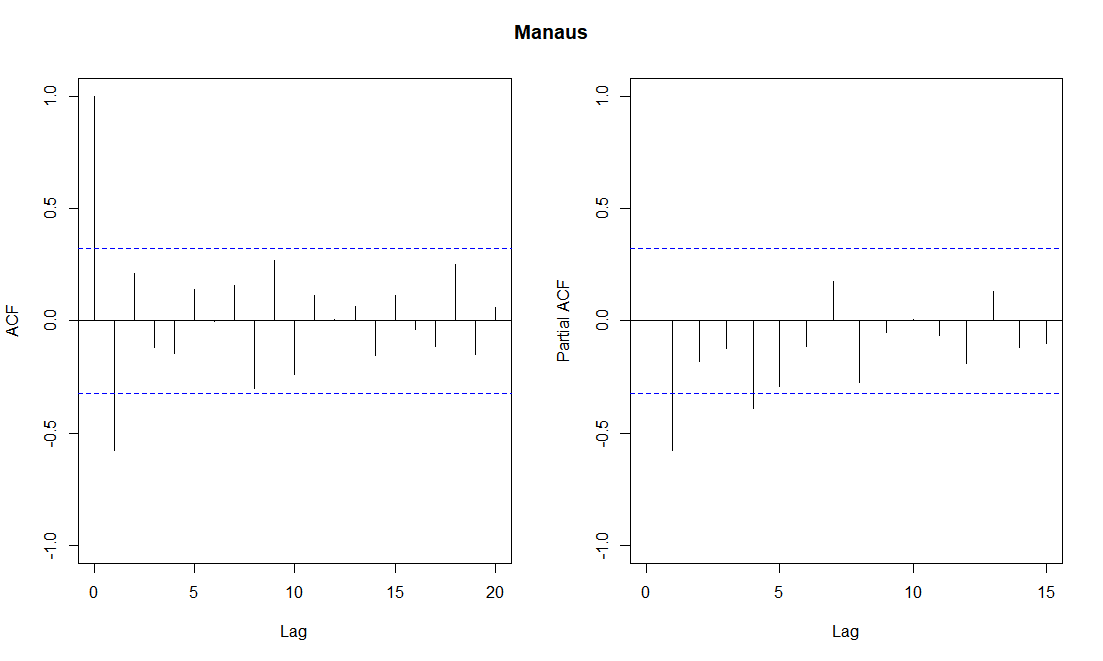


**Fig 16**. ACF and PACF for the city of Manaus (state of Amazonas)


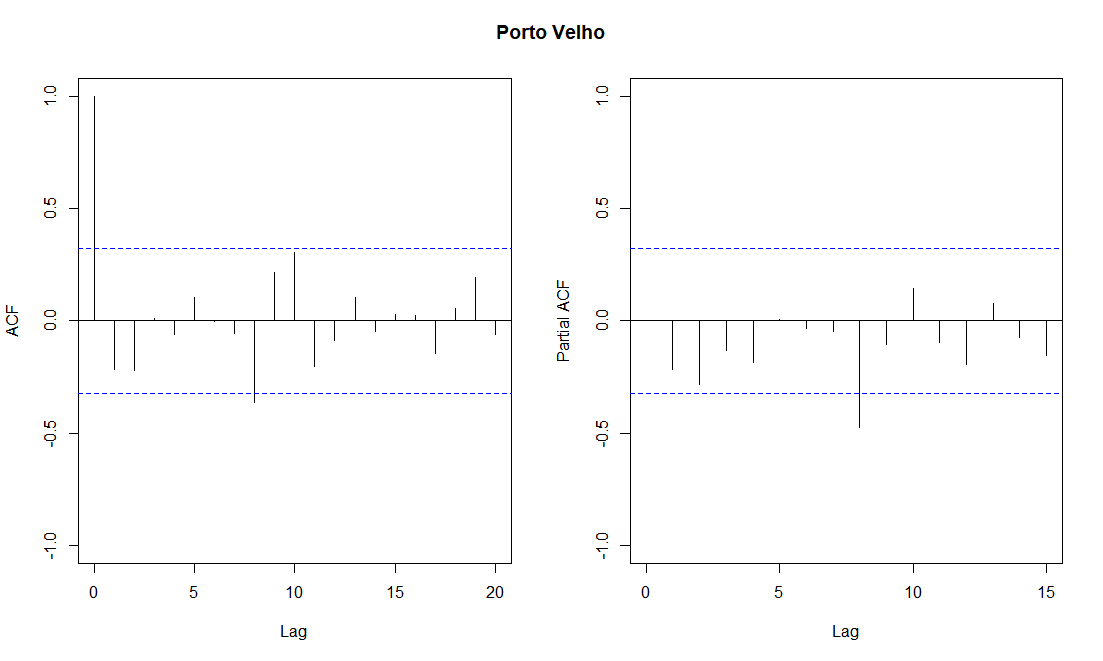


**Fig 17**. ACF and PACF for the city of Porto Velho (state of Rondônia)


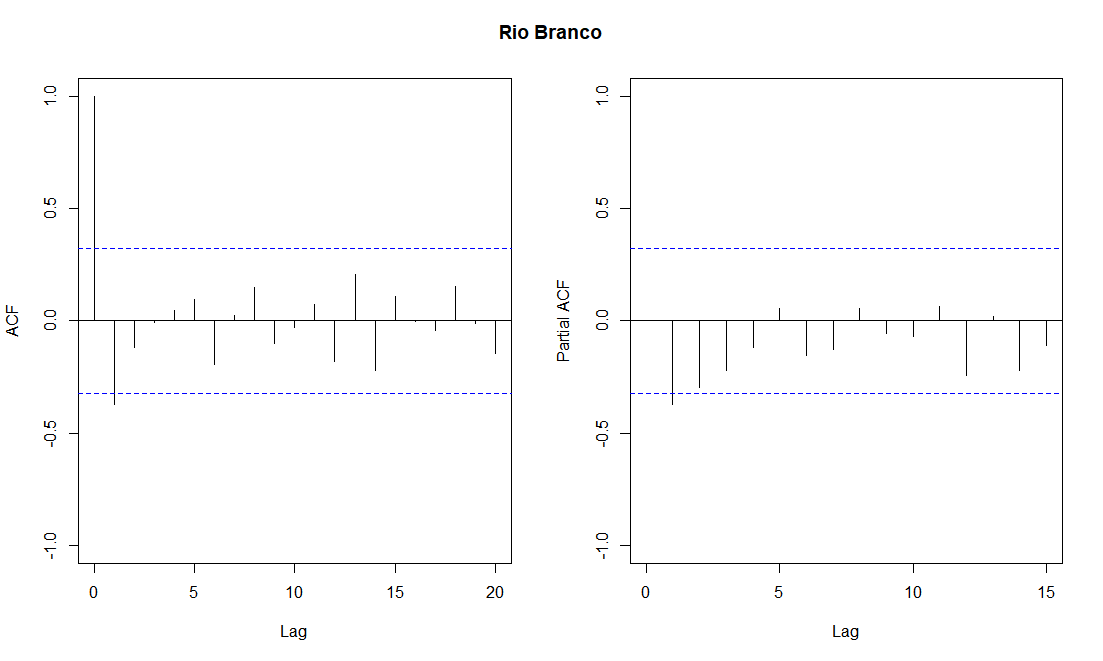


**Fig 18**. ACF and PACF for the city of Rio Branco (state of Acre). *ACF and PACF plotted after an outlier removal.

**Northeast macro-region**


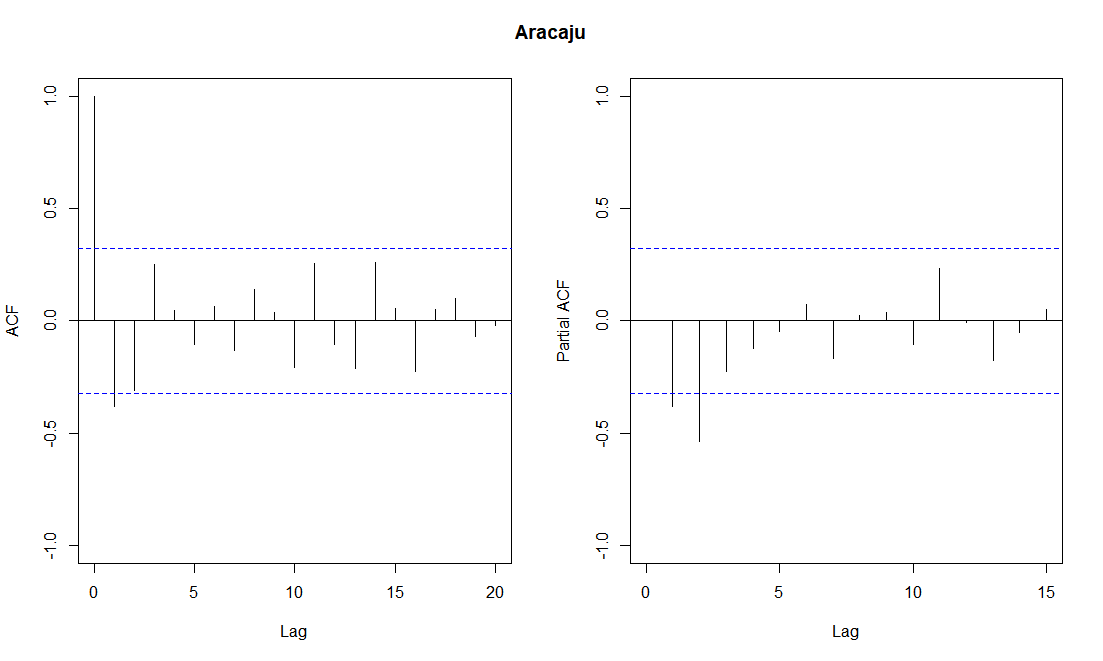


**Fig 19**. ACF and PACF for the city of Aracaju (state of Sergipe)


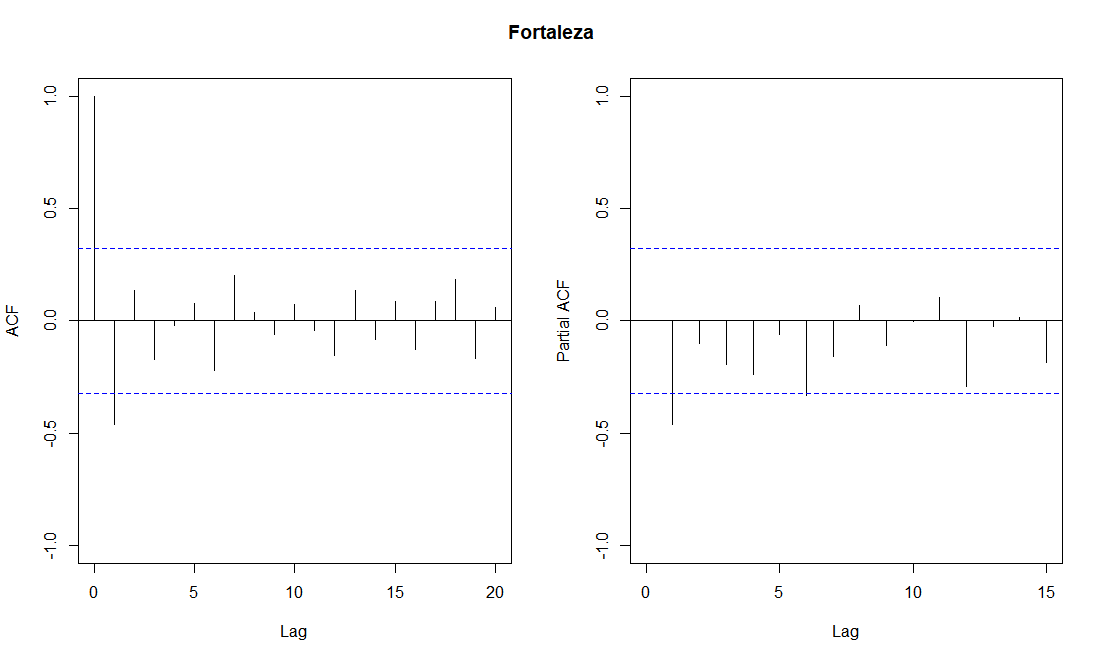


**Fig 20**. ACF and PACF for the city of Fortaleza (state of Ceará)


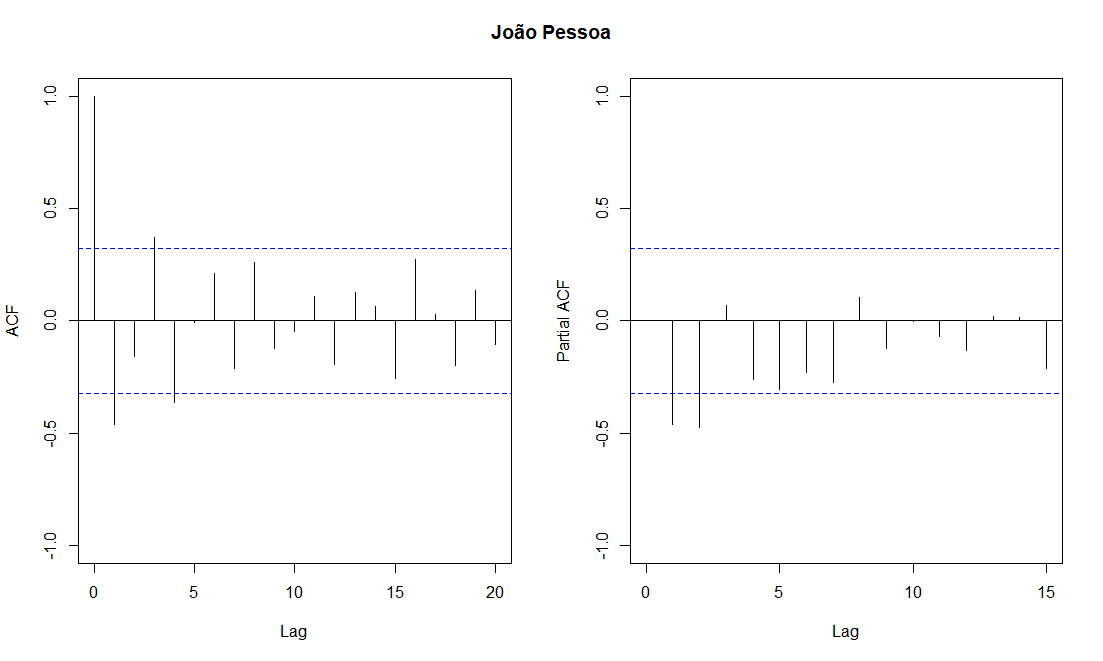


**Fig 21**. ACF and PACF for the city of João Pessoa (state of Paraíba)


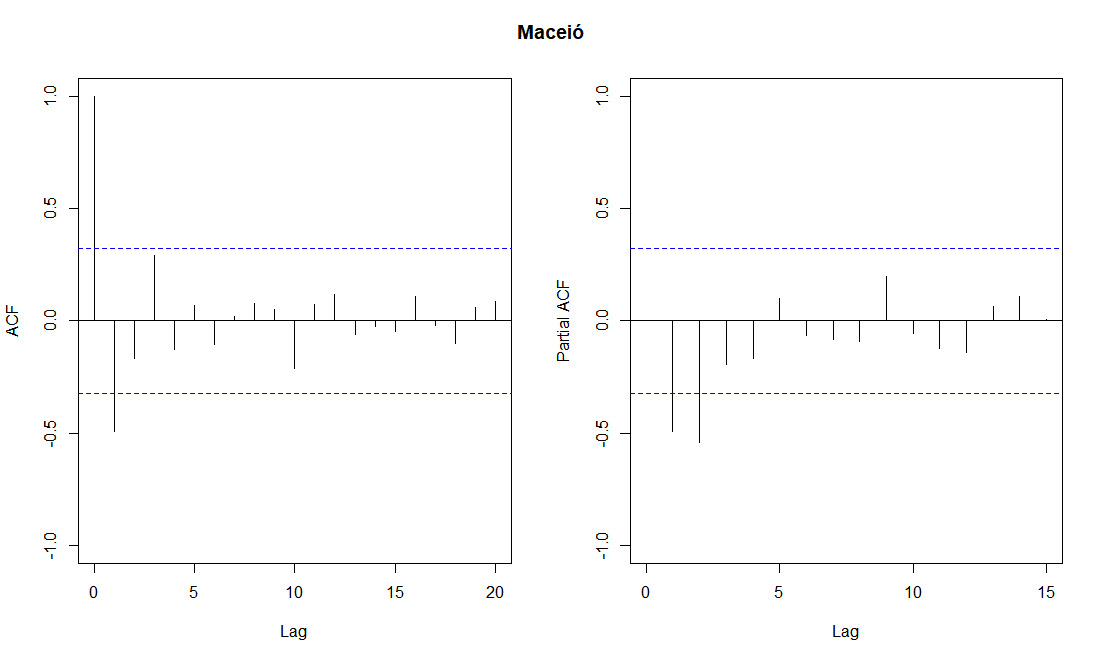


**Fig 22**. ACF and PACF for the city of Maceió (state of Alagoas)


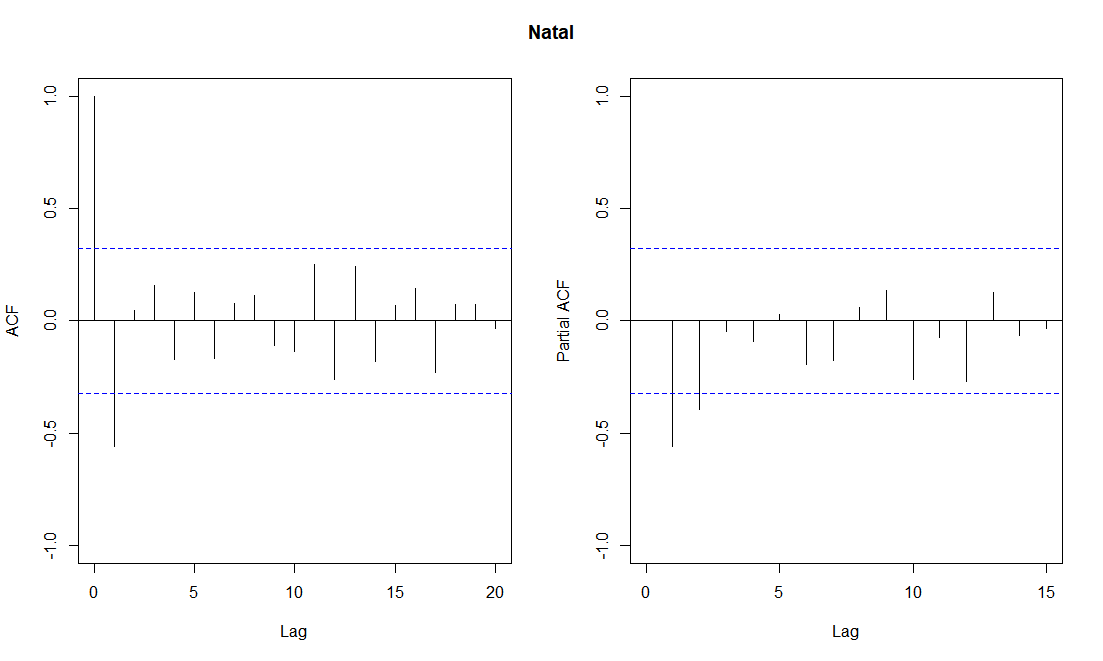


**Fig 23**. ACF and PACF for the city of Natal (state of Rio Grande do Norte)


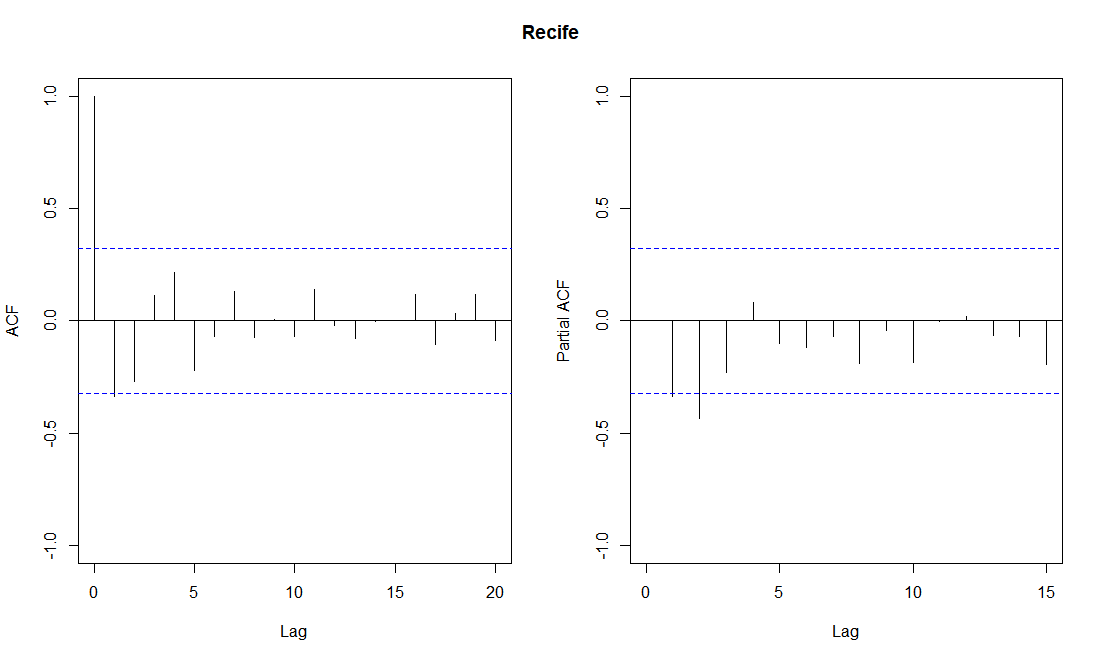


**Fig 24**. ACF and PACF for the city of Recife (state of Pernambuco)


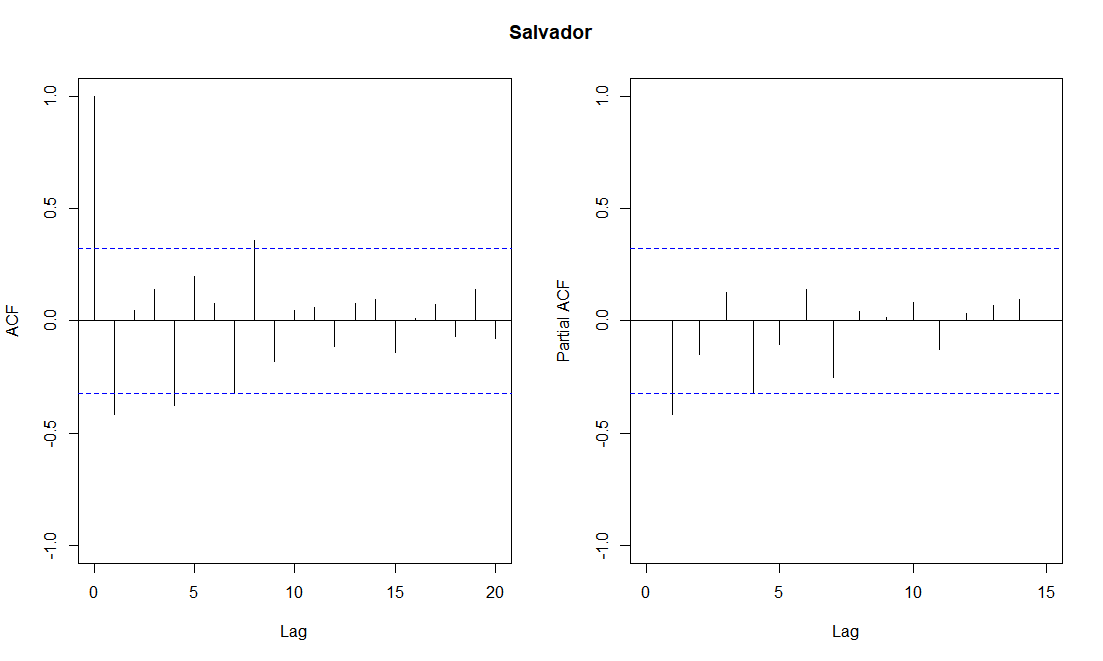


**Fig 25**. ACF and PACF for the city of Salvador (state of Bahia)


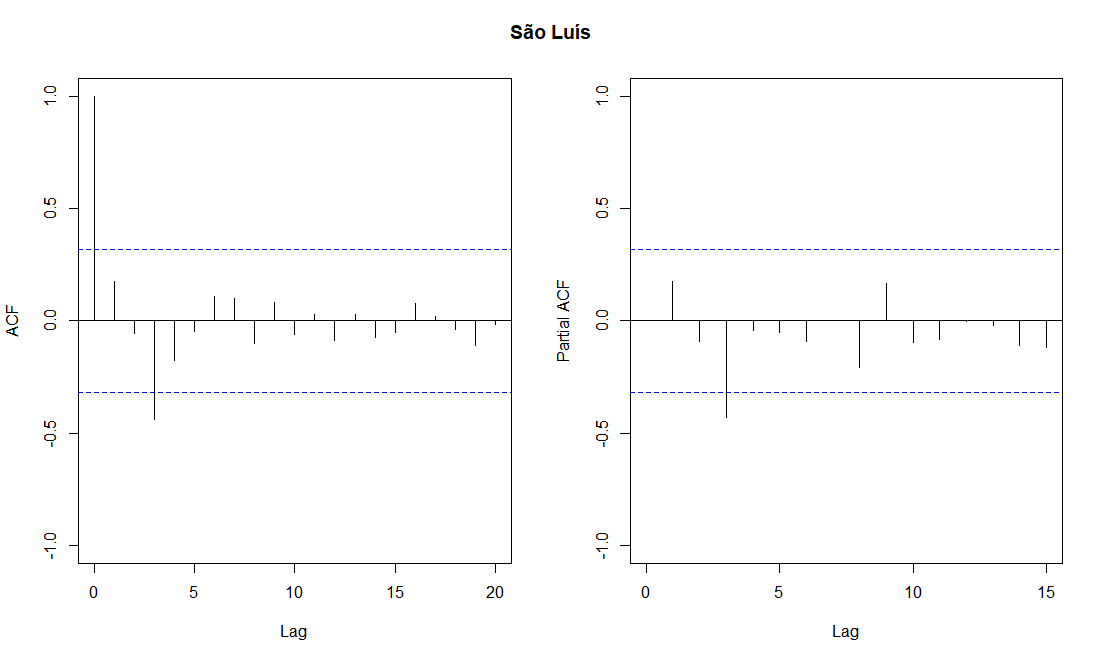


**Fig 26**. ACF and PACF for the city of São Luís (state of Maranhão)


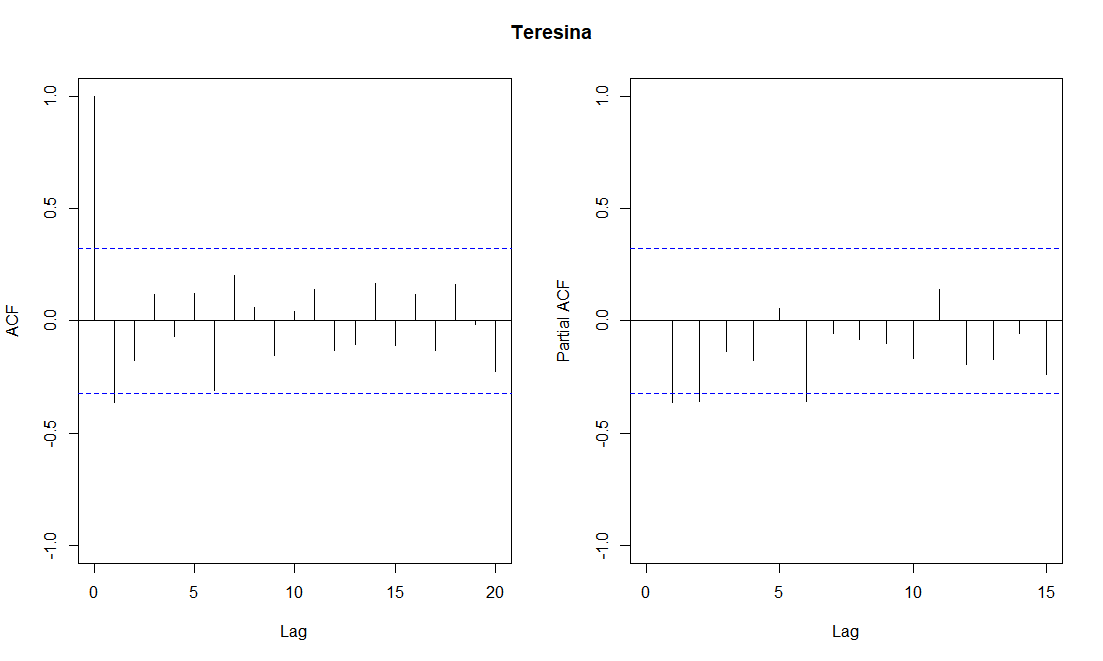


**Fig 27**. ACF and PACF for the city of Teresina (state of Piauí); *ACF and PACF plotted after an outlier removal.
