# Supplementary material for: Impact of the program life in traffic and new zero-tolerance drinking and driving law on the prevalence of driving after alcohol abuse in Brazilian capitals: An interrupted time series analysis
Source: PLoS One. 2023 Oct 20;18(10):e0288288. doi: 10.1371/journal.pone.0288288 (PMC10588900; doi:10.1371/journal.pone.0288288)
Supplement: S4 File — (DOCX) [file pone.0288288.s007.docx]

**File S4**. Residuals analysis

**Southeast macro-region**


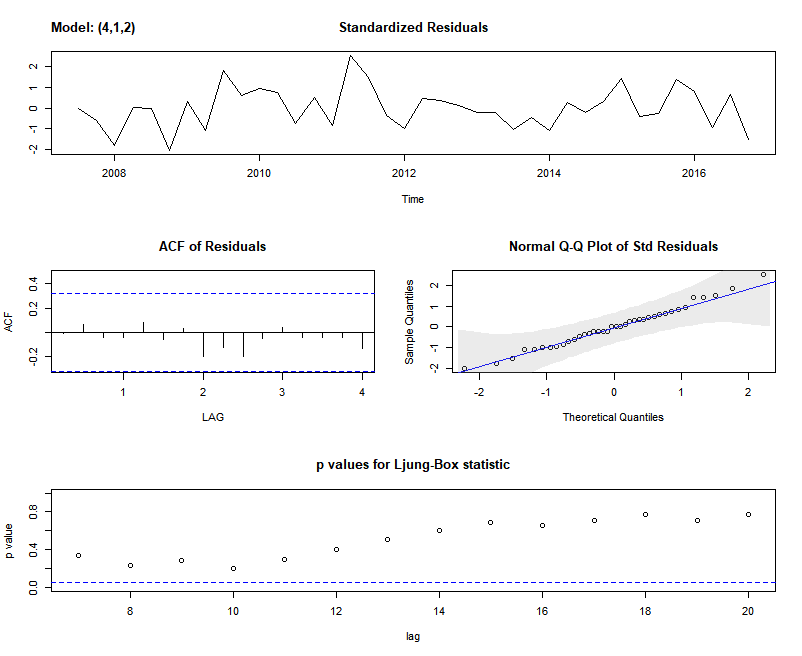


**Fig S3A**. ACF and PACF for the city of Belo Horizonte (state of Minas Gerais)


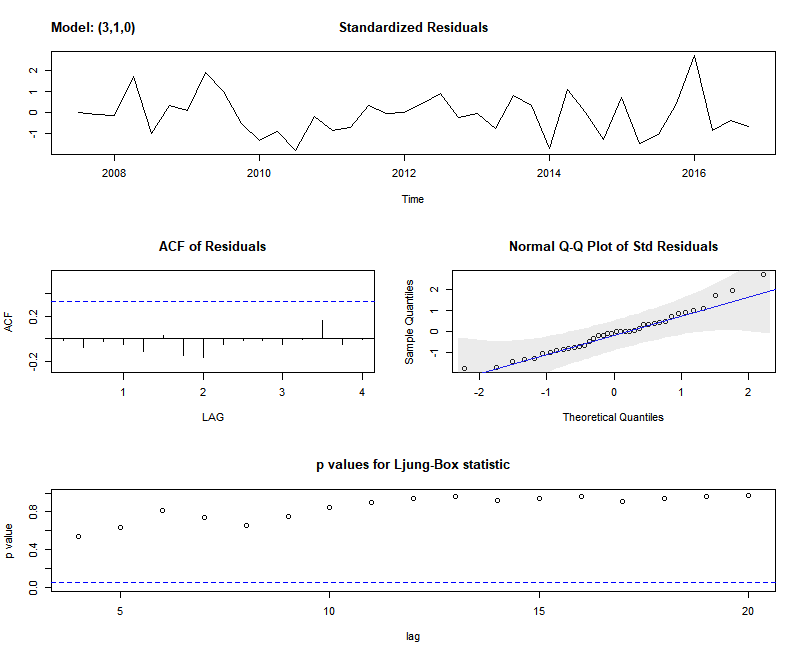


**Fig S3B**. ACF and PACF for the city of Rio de Janeiro (state of Rio de Janeiro)


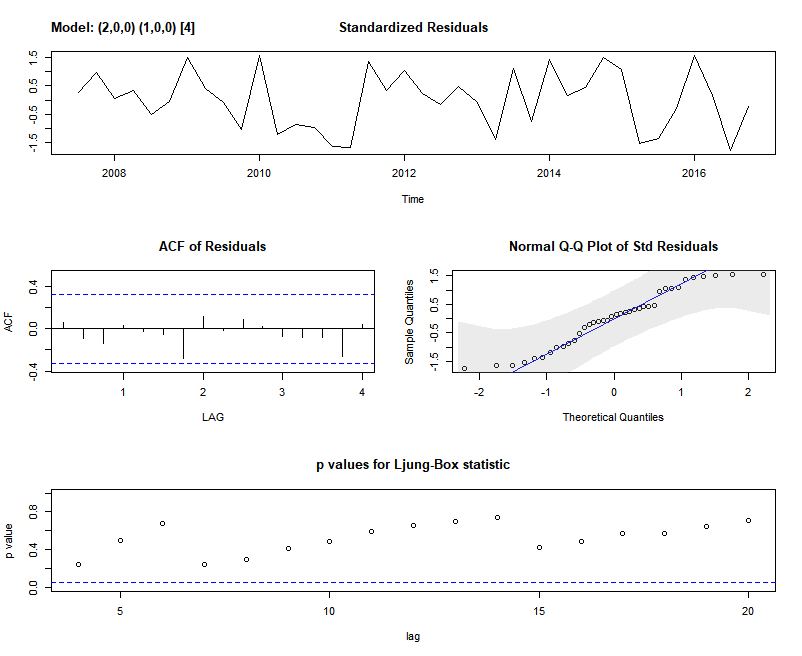


**Fig S3C**. ACF and PACF for the city of São Paulo (state of São Paulo)


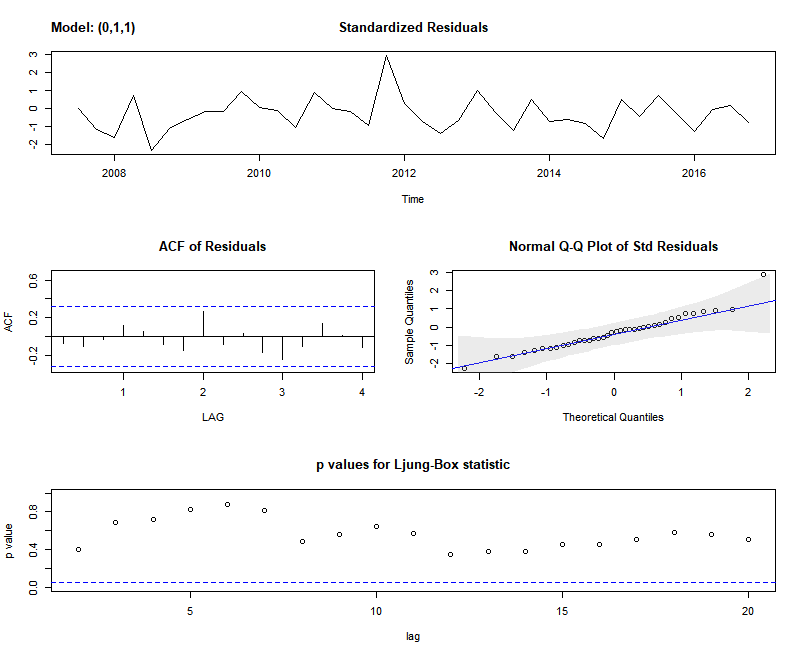


**Fig S3D**. ACF and PACF for Vitória City (state of Espírito Santo)

**South macro-region**

**
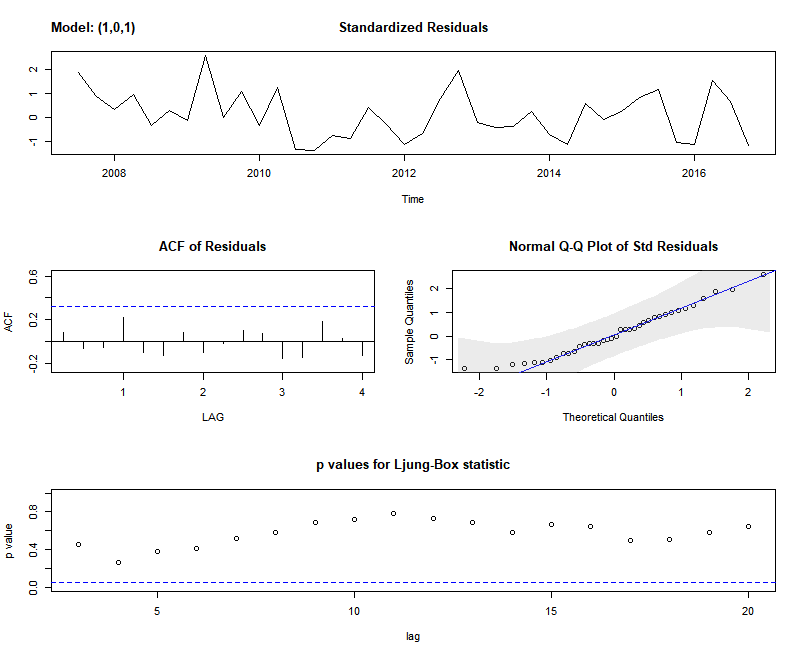
**

**Fig S4A**. ACF and PACF for the city of Curitiba (state of Paraná)

**
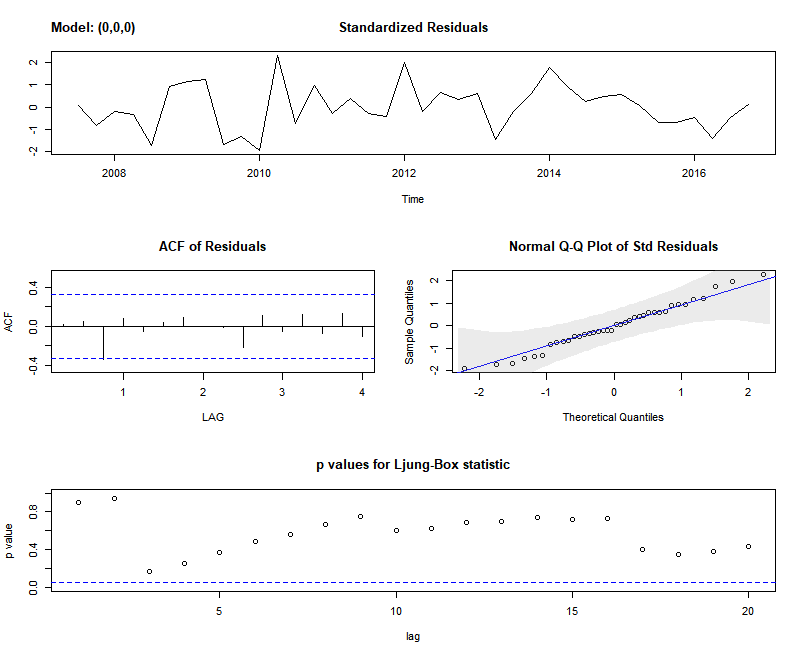
**

**Fig S4B**. ACF and PACF for the city of Florianópolis (state of Santa Catarina)


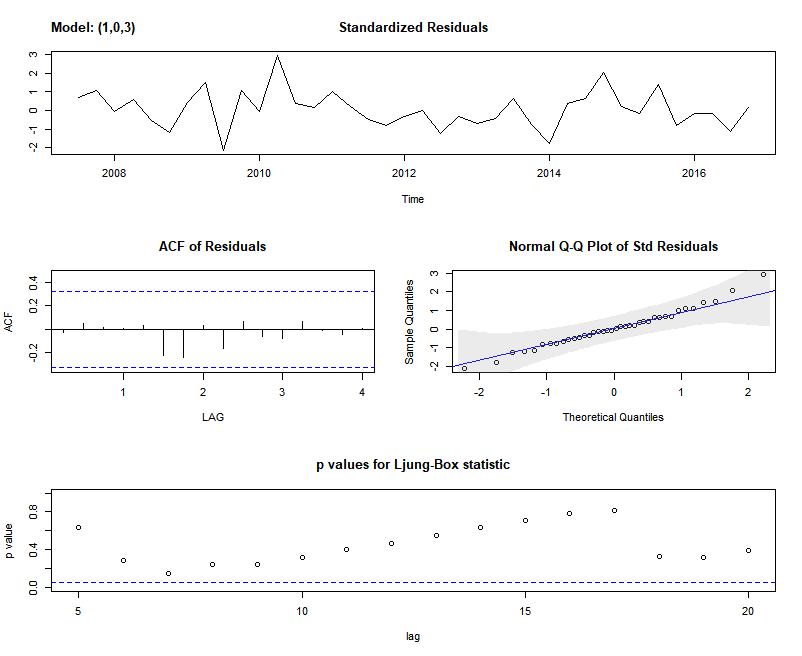


**Fig S4C**. ACF and PACF for the city of Porto Alegre (state of Rio Grande do Sul)

**Midwest macro-region**


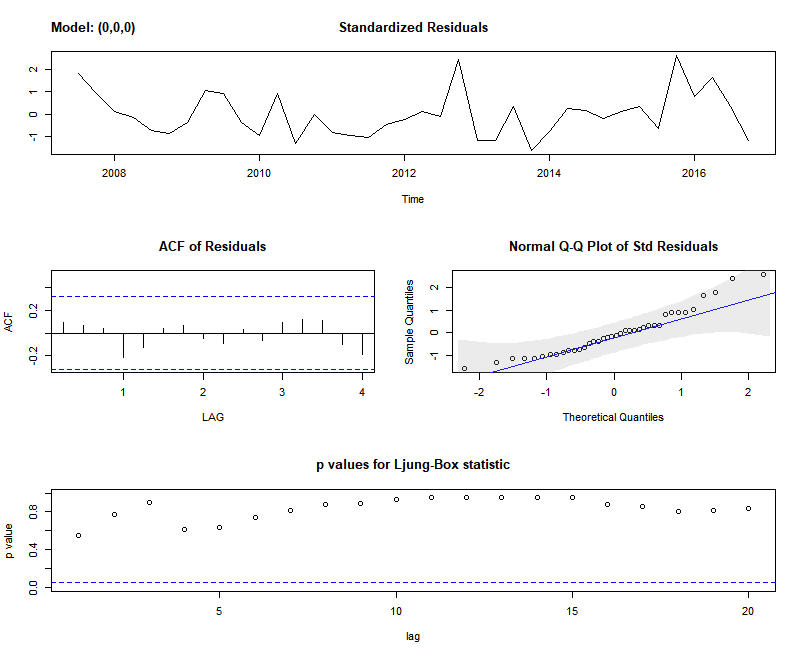


**Fig S5A**. ACF and PACF for Brasília (Distrito Federal)


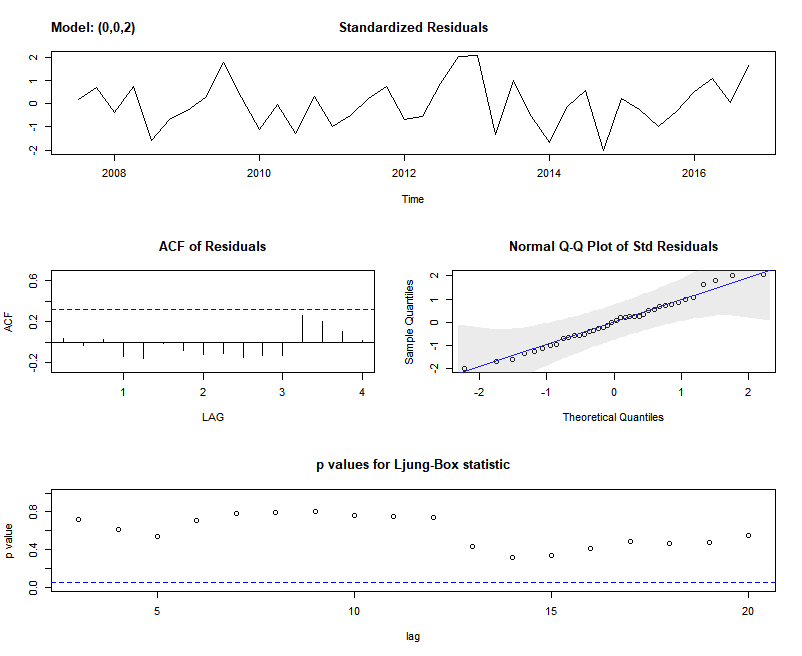


**Fig S5B**. ACF and PACF for the city of Campo Grande (state Mato Grosso do Sul)


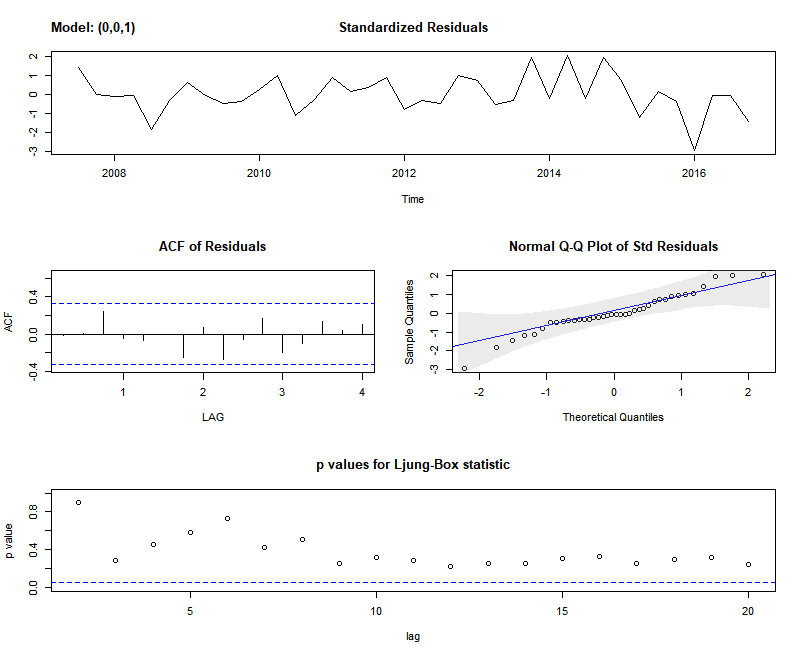


**Fig S5C**. ACF and PACF for the city of Cuiabá (state of Mato Grosso)


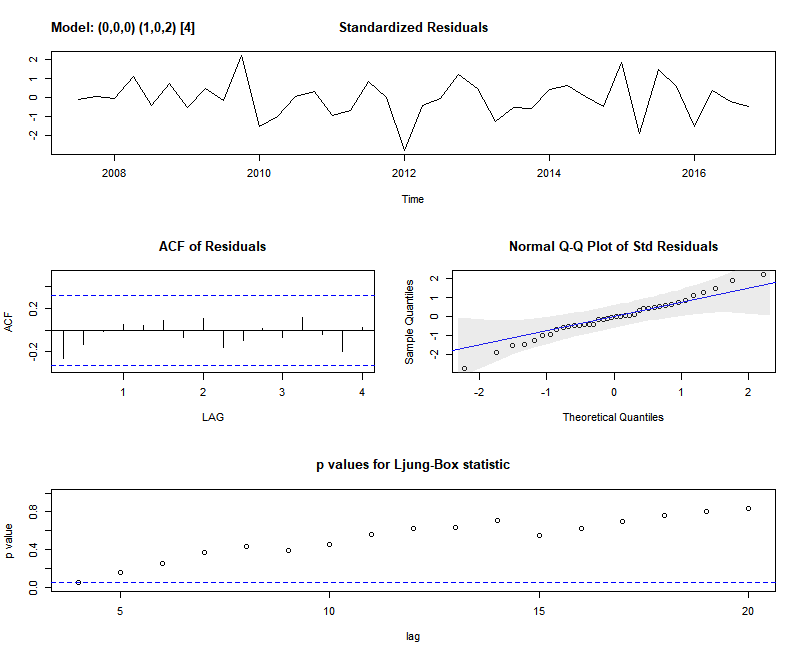


**Fig S5D**. ACF and PACF for the city of Goiânia city (state of Goiás)

**North macro-region**


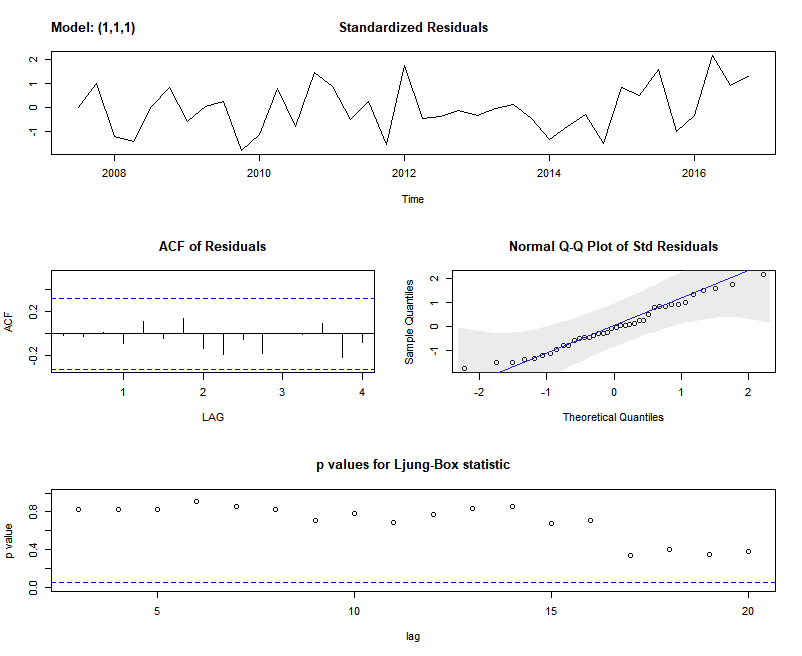


**Fig S6A**. ACF and PACF for the city of Palmas (state of Tocantins)


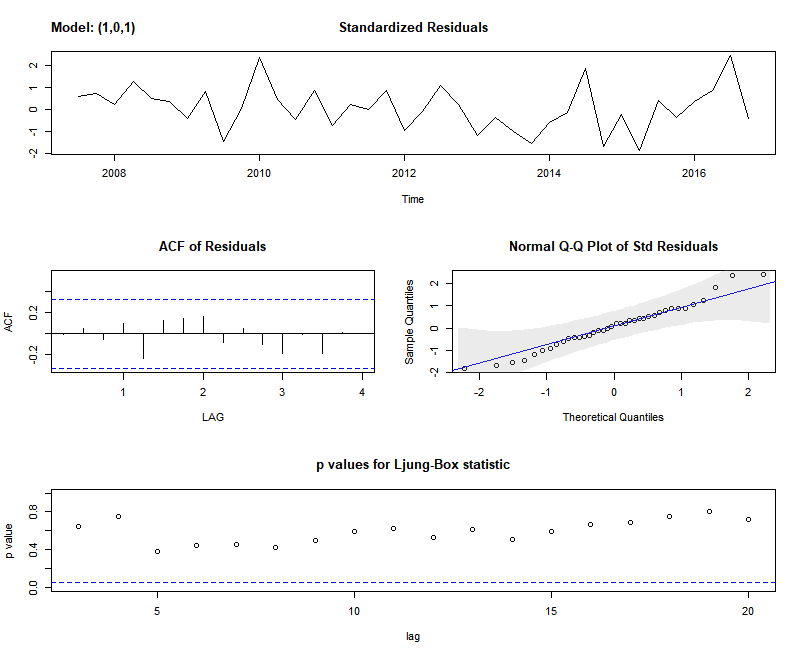


**Fig S6B**. ACF and PACF for the city of Belém (state of Pará)


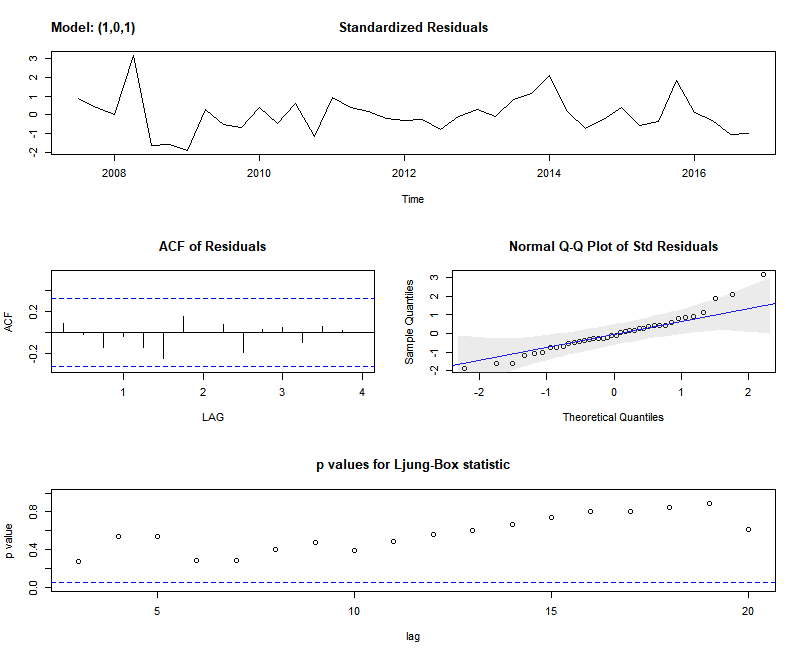


**Fig S6C**. ACF and PACF for the city of Boa Vista (state of Roraima)


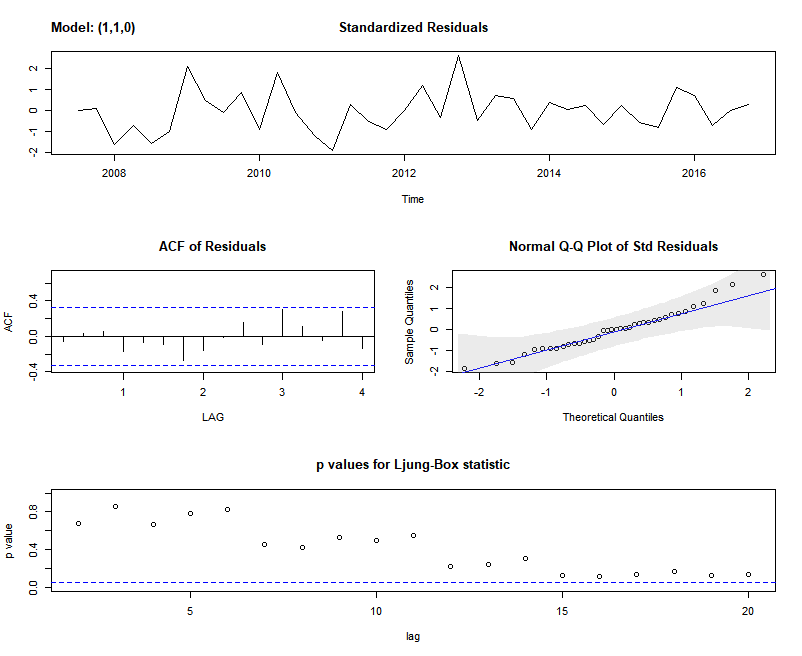


**Fig S6D**. ACF and PACF for the city of Macapá (state of Amapá)


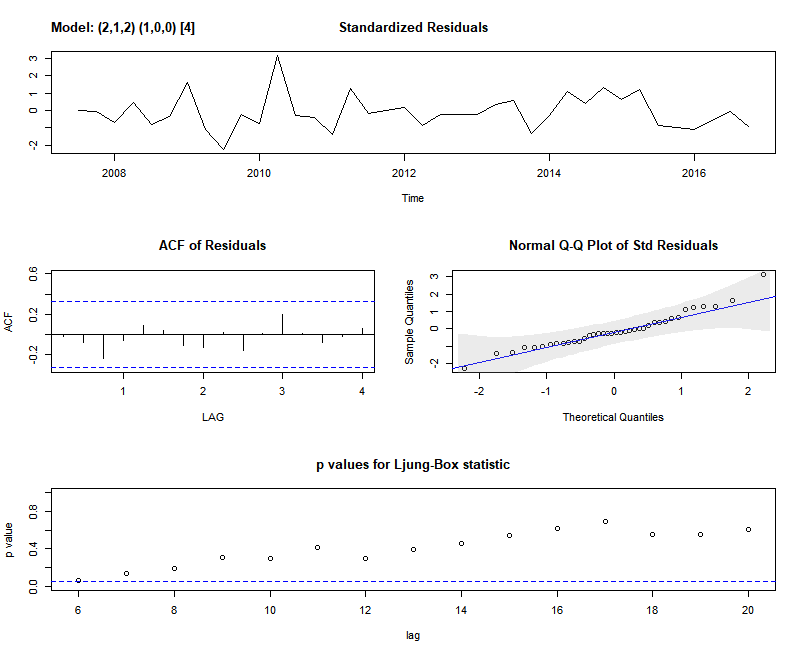


**Fig S6E**. ACF and PACF for the city of Manaus (state of Amazonas)


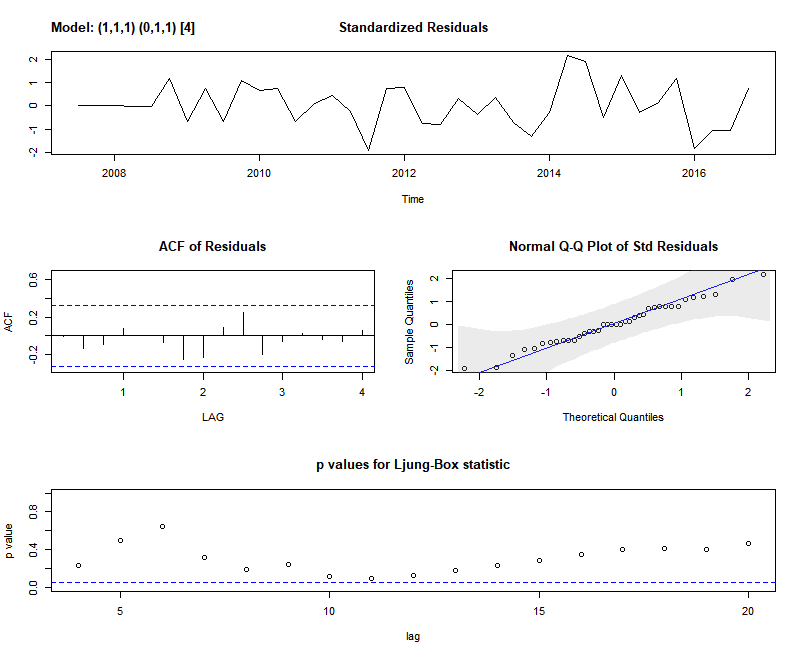


**Fig S6F**. ACF and PACF for the city of Porto Velho (state of Rondônia)


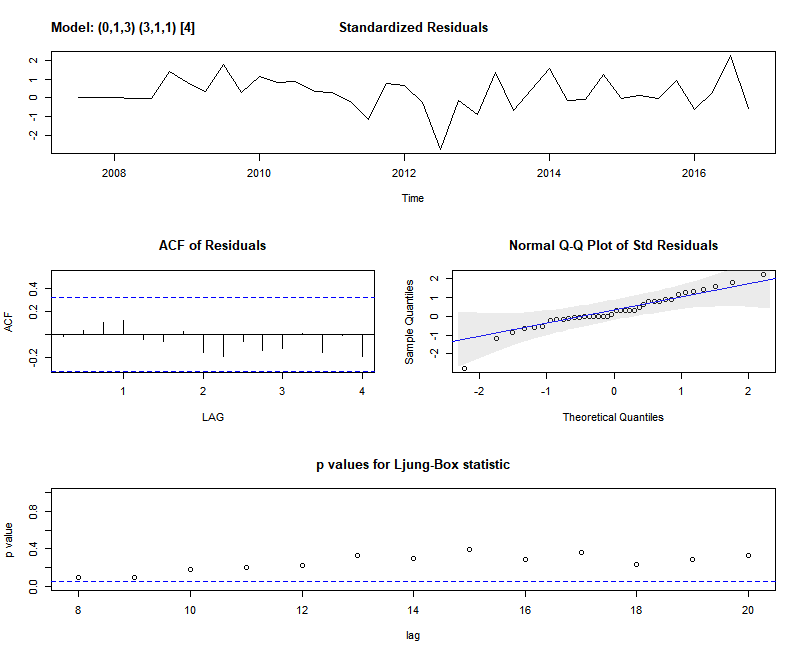


**Fig S6G**. ACF and PACF for the city of Rio Branco (state of Acre)

**Northeast macro-region**


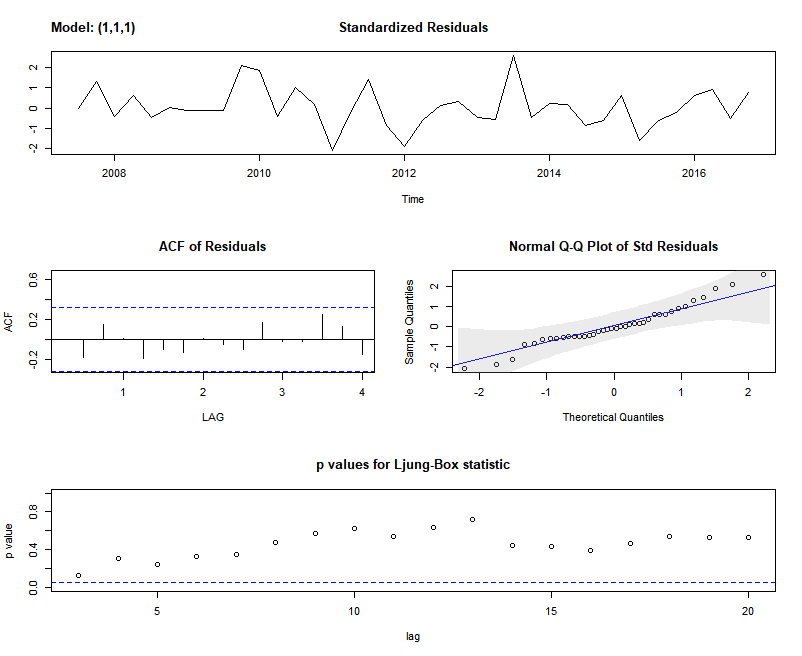


**Fig S7A**. ACF and PACF for the city of Aracaju (state of Sergipe)


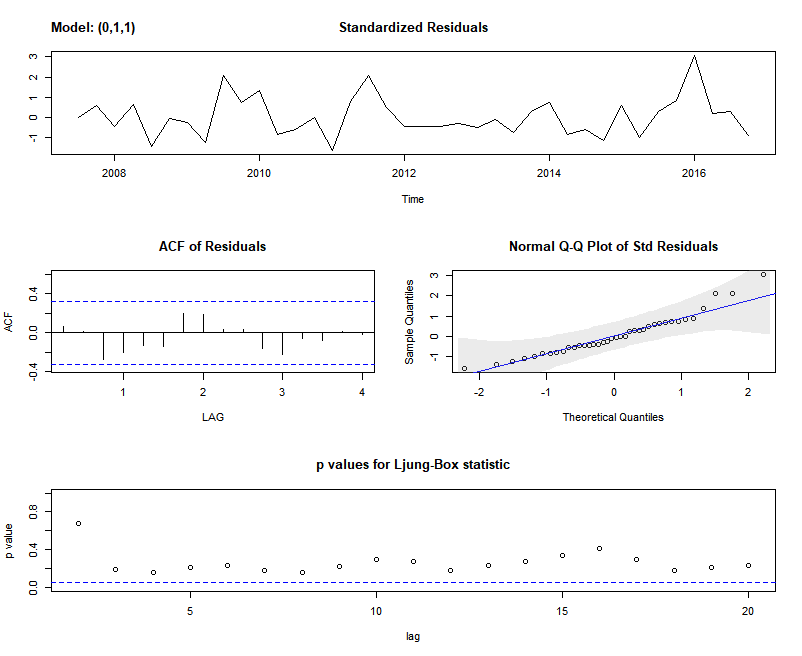


**Fig S7B**. ACF and PACF for the city of Fortaleza (state of Ceará)


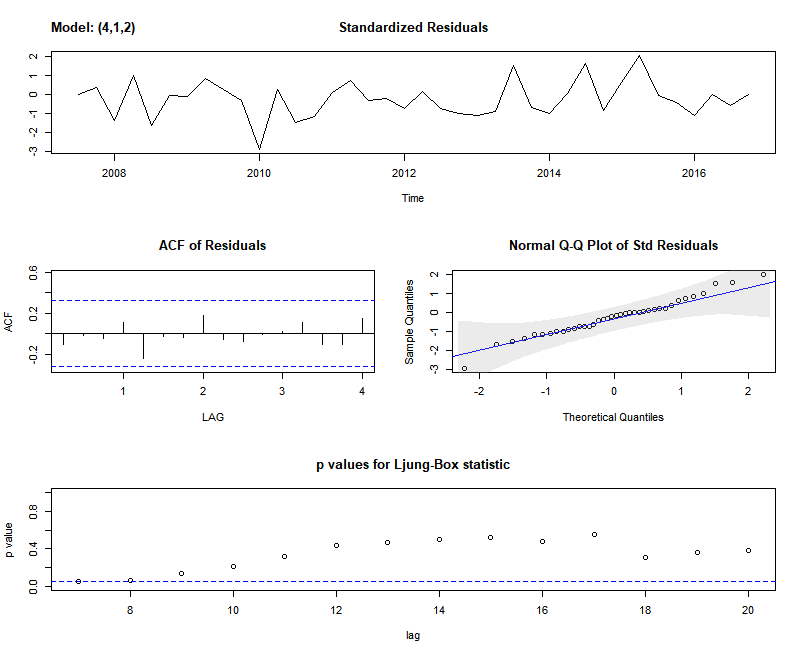


**Fig S7C**. ACF and PACF for the city of João Pessoa (state of Paraíba)


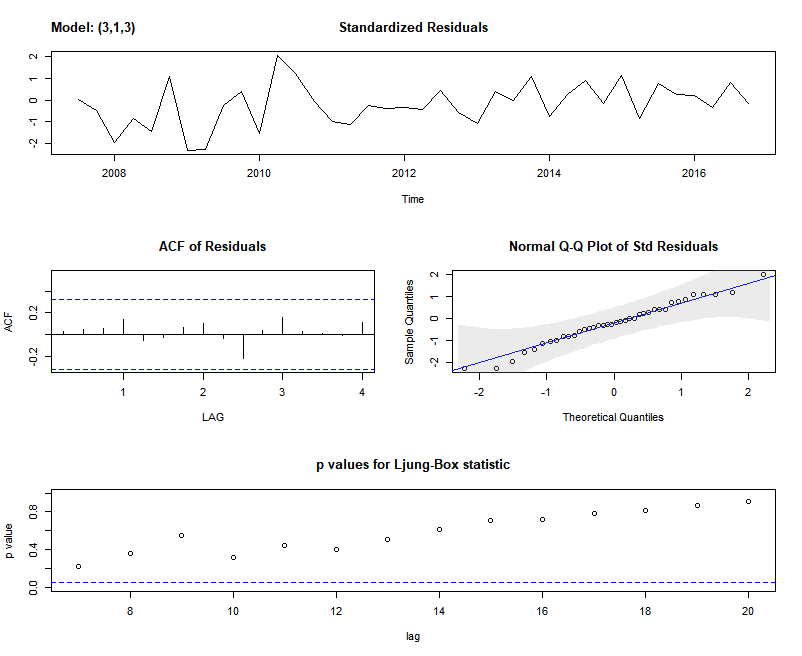


**Fig S7D**. ACF and PACF for the city of Maceió (state of Alagoas)


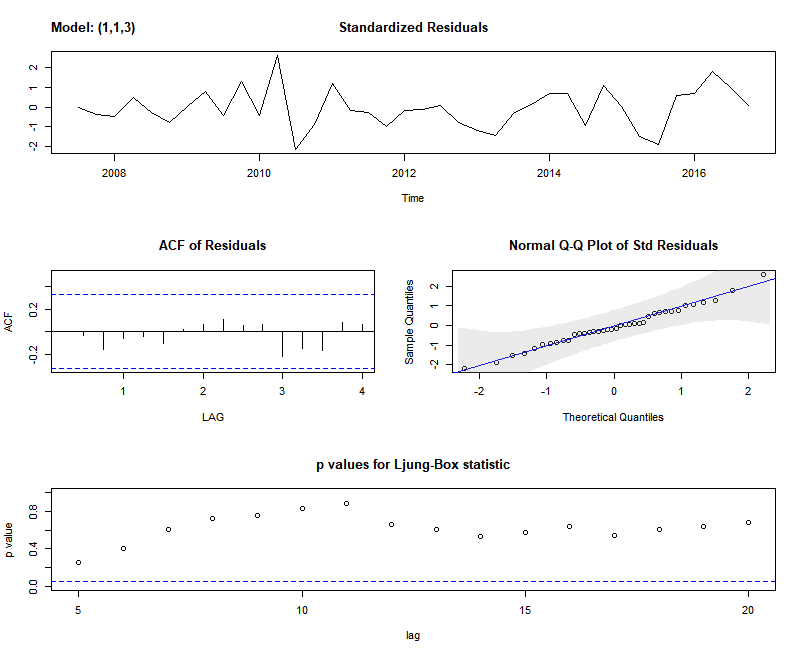


**Fig S7E**. ACF and PACF for the city of Natal (state of Rio Grande do Norte)


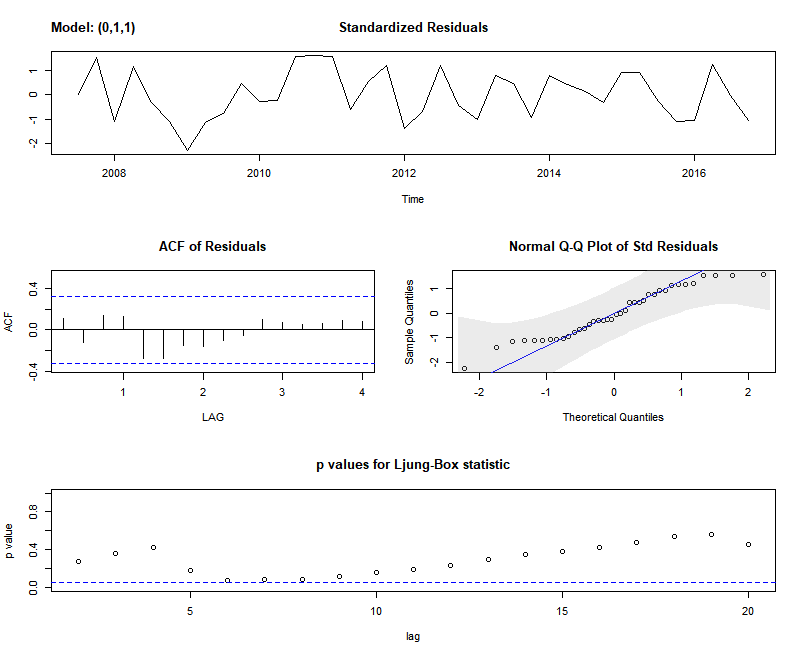


**Fig S7F**. ACF and PACF for the city of Recife (state of Pernambuco)


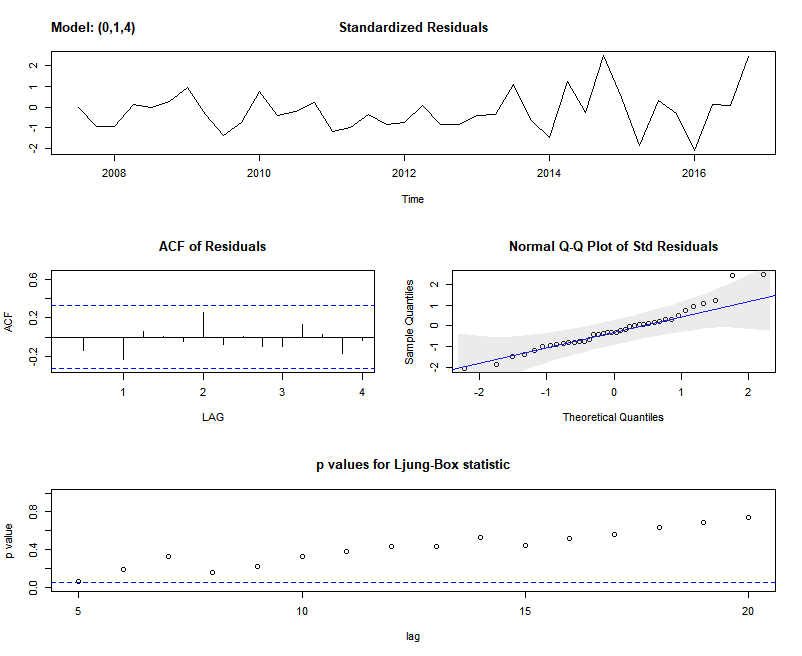


**Fig S7G**. ACF and PACF for the city of Salvador (state of Bahia)


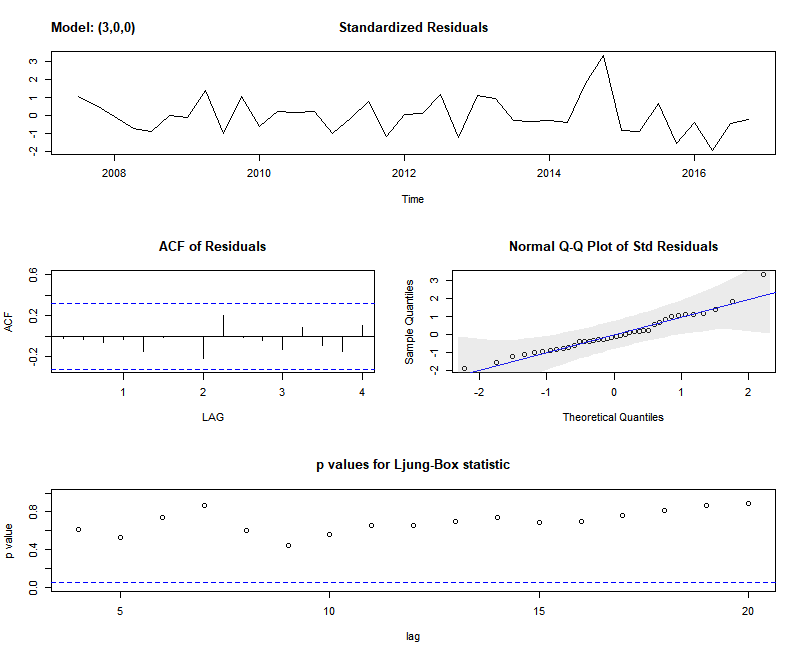


**Fig S7H**. ACF and PACF for the city of São Luís (state of Maranhão)


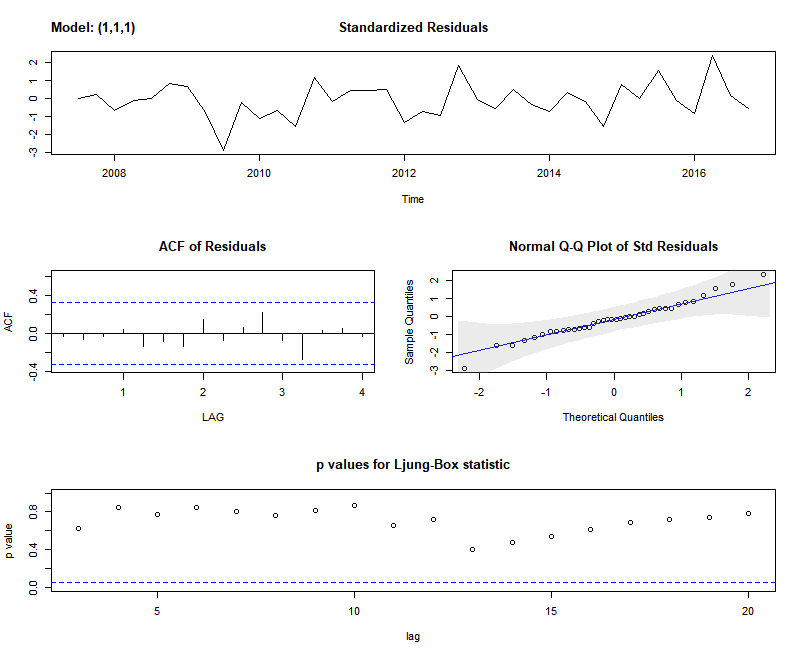


**Fig S7I**. ACF and PACF for the city of Teresina (state of Piauí)
